# Sandacrabins – Structurally Unique Antiviral RNA Polymerase Inhibitors from a Rare Myxobacterium**

**DOI:** 10.1002/chem.202104484

**Published:** 2022-01-22

**Authors:** Chantal D. Bader, Fabian Panter, Ronald Garcia, Egor P. Tchesnokov, Sibylle Haid, Christine Walt, Cathrin Spröer, Alexander F. Kiefer, Matthias Götte, Jörg Overmann, Thomas Pietschmann, Rolf Müller

**Affiliations:** ^1^ Helmholtz Institute for Pharmaceutical Research Saarland (HIPS) Helmholtz Centre for Infection Research (HZI) and Department of Pharmacy Saarland University Campus E8 1 66123 Saarbrücken Germany; ^2^ German Center for Infection Research (DZIF) Inhoffenstraße 7 38124 Braunschweig Germany; ^3^ Helmholtz International Lab for anti-infectives Campus E8 1 66123 Saarbrücken Germany; ^4^ Institute of Experimental Virology, TWINCORE Centre for Experimental and Clinical Infection Research a joint venture between the Medical School Hannover (MHH) and The Helmholtz Centre for Infection Research (HZI) Feodor-Lynen-Str. 7 30625 Hannover Germany; ^5^ Leibniz-Institut DSMZ - Deutsche Sammlung von Mikroorganismen und Zellkulturen Inhoffenstraße 7 and German Centre of Infection Research (DZIF) Partner Site Hannover-Braunschweig 38124 Braunschweig Germany; ^6^ Microbiology Technical University of Braunschweig 38106 Braunschweig Germany; ^7^ Department of Medical Microbiology and Immunology University of Alberta Edmonton Alberta Canada

**Keywords:** antiviral agents, natural products, structure elucidation, supercritical fluids, terpenoids

## Abstract

Structure elucidation and total synthesis of five unprecedented terpenoid‐alkaloids, the sandacrabins, are reported, alongside with the first description of their producing organism *Sandaracinus defensii* MSr10575, which expands the *Sandaracineae* family by only its second member. The genome sequence of *S. defensii* as presented in this study was utilized to identify enzymes responsible for sandacrabin formation, whereby dimethylbenzimidazol, deriving from cobalamin biosynthesis, was identified as key intermediate. Biological activity profiling revealed that all sandacrabins except congener A exhibit potent antiviral activity against the human pathogenic coronavirus HCoV229E in the three digit nanomolar range. Investigation of the underlying mode of action discloses that the sandacrabins inhibit the SARS‐CoV‐2 RNA‐dependent RNA polymerase complex, highlighting them as structurally distinct non‐nucleoside RNA synthesis inhibitors. The observed segregation between cell toxicity at higher concentrations and viral inhibition opens the possibility for their medicinal chemistry optimization towards selective inhibitors.

## Graphical abstract

**Non‐nucleoside RdRp inhibitors**. A family of unprecedented farnesylated dimethylbenzimidazoles called the sandacrabins have been discovered from a new myxobacterial *Sandaracinus* species. They exhibit antiviral activities that could be correlated to inhibition of the RNA synthesis as their potential antiviral mode of action. This study describes their structure elucidation, biosynthetic origin, total synthesis and biological evaluation.

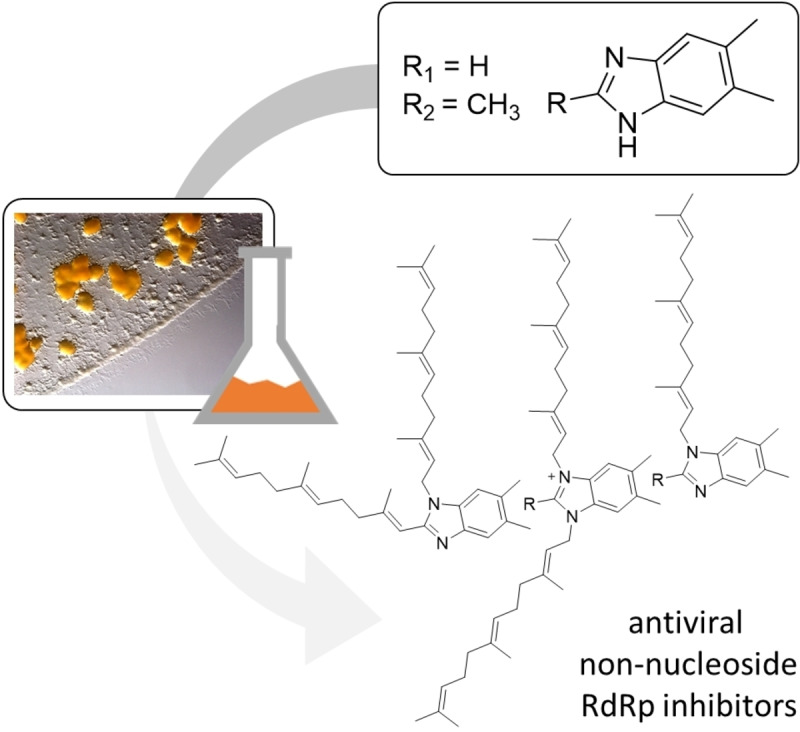

## Introduction

Natural products (NPs) often serve as a rational starting point for drug development as they commonly show intriguing biological activities based on their complex chemical scaffolds optimized during evolutionary processes.^[2]^ The evolving viral pandemics such as the COVID‐19 disease or swine flu and the progressive spread of antibacterial resistances‐for example the propagation of multidrug resistant tuberculosis‐led to an increased recurrence in natural product research to find promising starting points for the drug discovery pipelines. Besides the evaluation of already described NPs, identification and characterization of novel NPs is of high importance to develop new potential medicines.^[3]^ Among the NPs, bacterial secondary metabolites have already for a century greatly contributed to the stream of natural product based drug leads, especially in anti‐infective research and oncology.^[4]^

Myxobacteria, a phylum of ecologically diverse Deltaproteobacteria, are an especially prolific source of such structurally new NPs.^[5]^ They are known to exhibit a broad range of potent antimicrobial, cytotoxic and anti‐parasitic activities.^[6]^ Previously uncultured myxobacterial strains, especially those that show significant phylogenetic distance from well‐described genera, have proven to be a fruitful source of novel NPs featuring intriguing chemistry and biological activities.^[7]^ As part of our continuous screening efforts of novel myxobacterial strains for yet undescribed NPs, *Sandaracinus defensii* MSr10575 gained our attention. The only described member of the *Sandaracinaceae* family so far is *S. amylolyticus* NOSO‐4^T^, which was investigated on the genomic level for its starch degrading properties with emphasis on α‐amylases.^[8]^ Secondary metabolome screening of *S. amylolyticus* NOSO‐4 ^T^ furthermore led to the isolation of two prenyl indols (indiacen A and B) in a bioactivity‐guided isolation approach. These secondary metabolites were found to exhibit both antibacterial and antifungal properties.^[9]^ Production of indiacen A and B was also observed in *S. defensii* MSr10575 in a comparative study between supercritical fluid and conventional extraction, where we additionally detected myxochelin A and terrestribisamid A in the strain's extracts and characterized a group of plasmid‐encoded nonribosomal peptide‐polyketide (NRPS‐PKS) hybrids after activation of their underlying biosynthetic machinery, the sandarazols.^[10]^ Myxobacterial terpenoids such as the indiacens are of special interest, as they are significantly underrepresented within the known myxobacterial NPs, despite their biosynthetic gene clusters (BGCs) being not particularly rare.^[11]^ Besides some small terpenoids, such as geosmin and germacradienol^[12]^‐which were first discovered and isolated from actinobacteria‐only few myxobacterial terpenes, such as salimyxin, cystodienoic acid, enhygromic acid or the aurachins, were described.[^13^, ^14^] Among those, only the strongly bioactive aurachins belong to the class of alkaloid terpenoids, making the sandacrabins‐unprecedented terpenoid‐alkaloid NPs which we describe in this article‐a promising target for further characterization.[^14^, ^15^]

## Results and Discussion

### Strain and genome description

**Figure 1:**
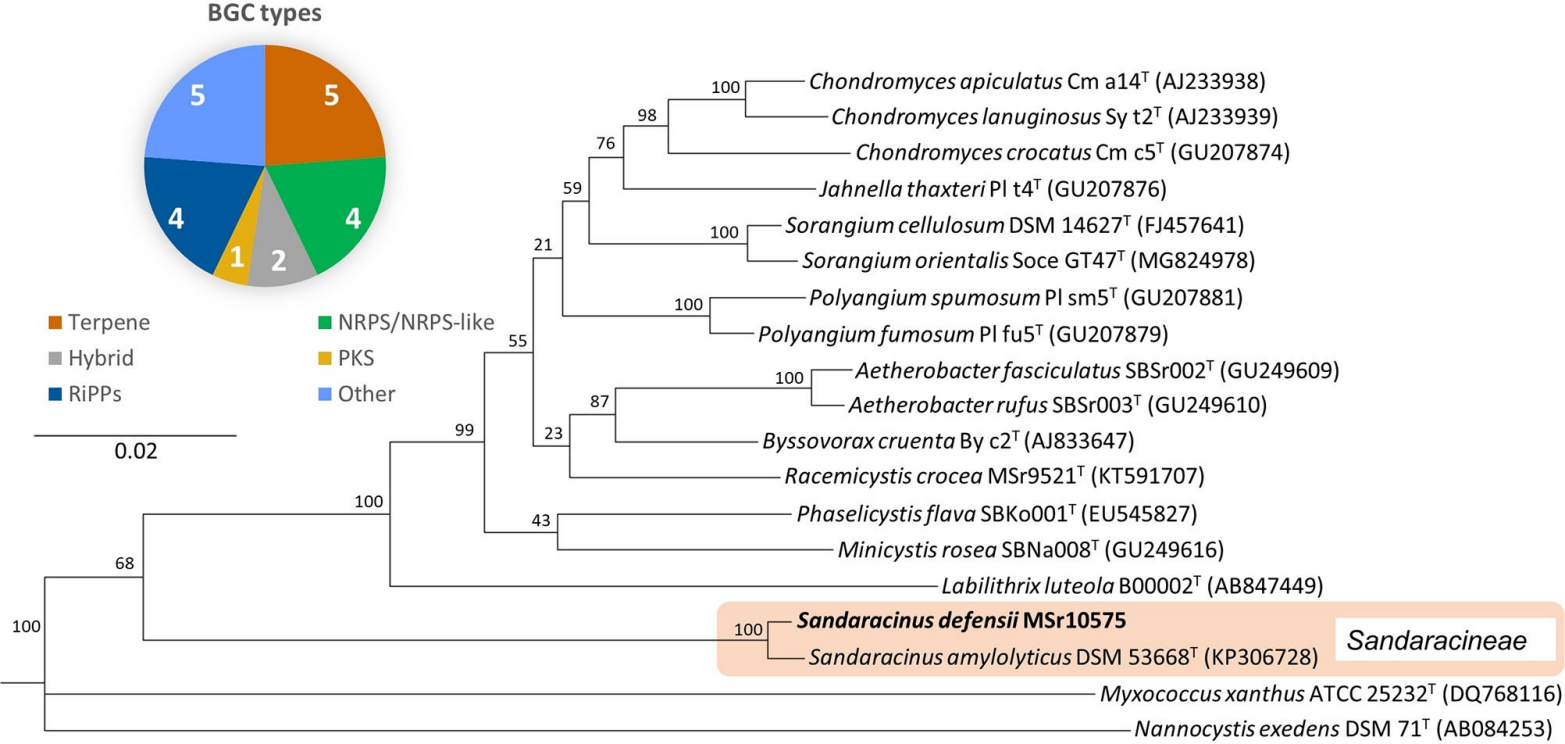
Phylogenetic classification of *S. defensii* MSr10575 as well as BGCs found in its genome using AntiSmash.^[1]^

*S. defensii* MSr10575 forms yellowish‐orange colonies with irregular edges towards the colony margin in axenic culture and was isolated in 2013 from the HZI soil collection (formerly GBF soil collection). Based on 16S rRNA gene phylogenetic analysis, MSr10575 was positioned among the *Sorangiineae* suborder in the *Sandaracinus* clade (see Figure 1), indicating that it belongs to the yet underexplored myxobacterial genera. The 16S rRNA sequencing and subsequent BLAST (Basic Local Alignment Search Tool) search revealed closest similarity of the strain (99.4 %) to *S. amylolyticus* NOSO4^T^ (GenBank accession number: KP306728).^[16]^ Its affiliation with the *Sandaracinus* clade was based on 16S rRNA gene sequence phylogenetic analysis, while the proposal for a new species was based on the average nucleotide identity (OrthoANI) and DNA‐DNA hybridization (DDH) (see Supporting Information) of the strain's PacBio genome sequence. Strain MSr10575 exhibits starch degrading properties comparable to *S. amylolyticus* NOSO4^T^, with the respective α‐amylases (five α‐amylase type and one α‐1‐6 glucosidase genes) present in its genome (see Supporting Information).

*S. defensii* presents a genome size of 10.754 Mb, which is above the bacterial average, for which myxobacteria are well‐known. We predict a total number of 21 BGCs by AntiSmash^[1]^ in the strains chromosomal DNA, with only one of those BGCs‐namely the myxochelin BGC‐correlated to the respective product yet. 19 % of the predicted BGCs belong to the ribosomally synthesized and post‐translationally modified peptides (RiPPs) and nonribosomal peptide synthetase (NRPS) or NRPS‐like BGCs each, while 9 % represent hybrids. The amount of polyketide encoding BGCs is comparably low with only one pure PKS cluster detectable in the genome. Interestingly, the majority of BGCs (24 %) are predicted to produce terpenoid NPs, highlighting the strains excellent potential for the discovery of novel molecules belonging to this underrepresented NP class in myxobacteria.

A complete description of the strain morphology and purification can be found in the Supporting Information. The respective genome sequence is deposited in GenBank alongside with the publication of this manuscript.

### Isolation and structure elucidation of the sandacrabins

**Figure 2:**
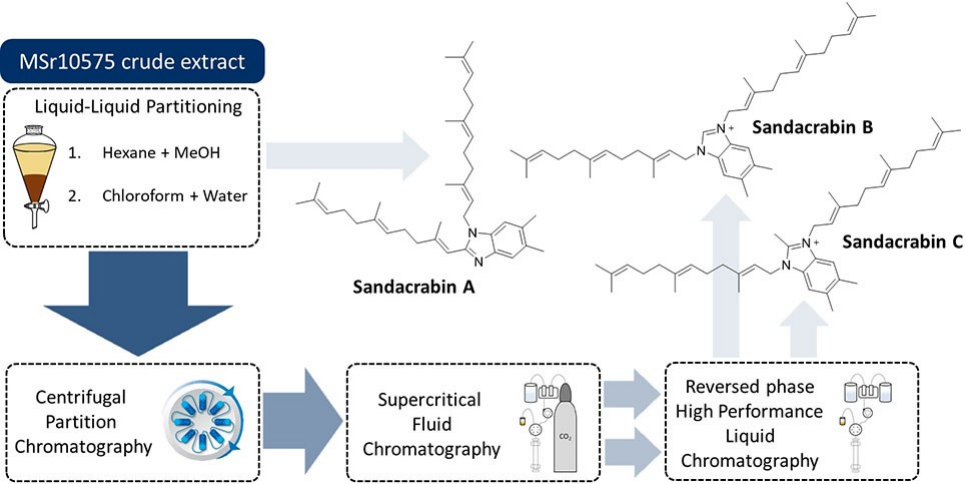
Structural formulae of sandacrabin A, B and C and the respective methods used for their purification.

In‐depth analysis of the *S. defensii* MSr10575 metabolome by high performance liquid chromatography coupled to mass spectrometry (HPLC‐MS) revealed a group of three peaks at *m/z* 541.45, 555.47 and 569.48 in positive ESI ionization mode. The three corresponding secondary metabolites were named sandacrabins A−C. According to sum formula predictions based on HRMS data, their sum formula lacks oxygen atoms commonly present in polypeptide and polyketide NPs, which suggested their classification among the alkaloid family of terpenoid NPs. Sandacrabin A was purified by semi‐preparative reversed phase (RP) HPLC from the dried hexane layer obtained by partitioning of a *S. defensii* MSr10575 crude extract between methanol and hexane. The high lipophilicity of sandacrabin B and C, along with their structural similarity, however, required a four‐step purification process (see Figure 2). After separation of sandacrabin A from the crude extract, the remaining methanol layer was dried and partitioned between water and chloroform. The dried chloroform layer was first subjected to centrifugal partitioning chromatography (CPC). Separation of sandacrabin B and C from the respective CPC fractions was subsequently achieved by supercritical fluid chromatography (SFC) prior to isolation of the secondary metabolites by semi‐preparative RP‐HPLC.

**Figure 3:**
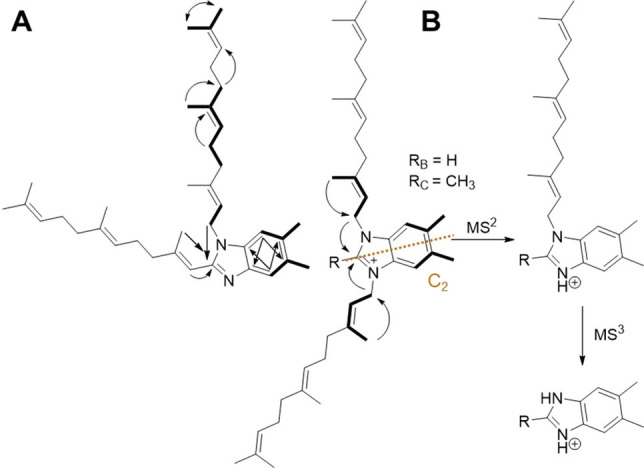
A Key NMR correlations used for structure elucidation. COSY correlations: bold line. HMBC correlations: arrows. Dashed orange line: symmetry axis of sandacrabin B and C. **B** Prominent MS^n^ fragments of sandacrabin B and C.

HRESI‐MS analysis of sandacrabin A showed an [M+H]^+^ signal at *m/z* 541.4514 (calc. 541.4516 Δ=0.4 ppm) consistent with the sum formula of C_38_H_57_N_2_ containing 12 double bond equivalents (DBEs). 1D and 2D NMR spectra of sandacrabin A (Tables see Supporting Information) suggested a benzimidazole core structure of the molecule. The splitting pattern of the two aromatic protons in line with their COSY correlations furthermore revealed a 5,6‐dimethyl substitution of this unit. COSY and HMBC correlations showed arrangement of the remaining methylene and methyl groups in two farnesyl moieties on the 5,6‐dimethylbenzimidazole (DMB) core. HMBC correlations of the aliphatic double bond proton at *δ*(^1^H)=6.22 ppm, as well as its downfield shift, showed that the second farnesyl side chain substitutes the 2‐position of DMB (see Figure 3A).

HRESI‐MS analysis of sandacrabin B and C showed an [M]^+^ signal at *m/z* 555.4514 (calc. 555.4673 Δ=0.2 ppm) corresponding to the sum formula of C_39_H_59_N_2_ and 569.4825 (calc. 569.4829 Δ=0.7 ppm) corresponding to the sum formula of C_40_H_61_N_2_, respectively, with both containing 12 DBEs, comparable to sandacrabin A. 1D and 2D NMR‐spectra of sandacrabin B and C (tables see supporting information), revealed a C_2v‐_symmetry (see Figure 3A) and a bisfarnesylated DMB core structure for both derivatives. The C_2v_‐symmetry of the molecules, as well as their permanent positive charge, indicated a 1,3‐substitution of the DMB unit. In contrast to sandacrabin B, 1D and 2D NMR‐spectra of sandacrabin C revealed a methylation in the 2‐position of the DMB core. Tautomerization of the imidazole double bond as well the symmetry axis passing through this part of the molecule however hindered a detection of the corresponding proton signal at this position in sandacrabin B, wherefore HRESI‐MS^3^ spectra of the three sandacrabins were used to provide additional proof for their structures (see Figure 3B).

The shielded shift of all methyl carbons indicates an all‐*E* configuration of all sandacrabin derivatives, which was later on confirmed by comparison of the NMR spectra with the synthetically derived congeners.

### Biosynthesis of the sandacrabins

To the best of our knowledge, there are no farnesylated 5,6‐dimethyl benzimidazoles described from bacteria yet, so we became interested in elucidating their biosynthetic origin. The sandacrabin core unit DMB likely derives from the respective pathway that supplies this biosynthetic precursor to the cobalamin (vitamin B12) biosynthesis pathway, similar to the biosynthesis very recently described for the myxadazoles.^[17]^ The BluB enzyme, involved in this pathway oxidizes riboflavin to form DMB.^[18]^ On the other hand, DMB biosynthesis can also derive from 5‐aminoimidazole ribotide, which is converted to 1H‐benzo[*d*]imidazole‐5‐ol by BzaA, D and F (see Figure S2). Subsequent methylation steps are carried out by BzaC and BzaD, before the resulting 5‐methoxy‐1H‐benzo[d]imidazole is converted to DMB by BzaE.^[19]^ Homologues for these enzymes can be found in the genome of *S. defensii* MSr10575 (see Supporting Information), which underlines the ability of the strain to synthesize DMB.

**Figure 4:**
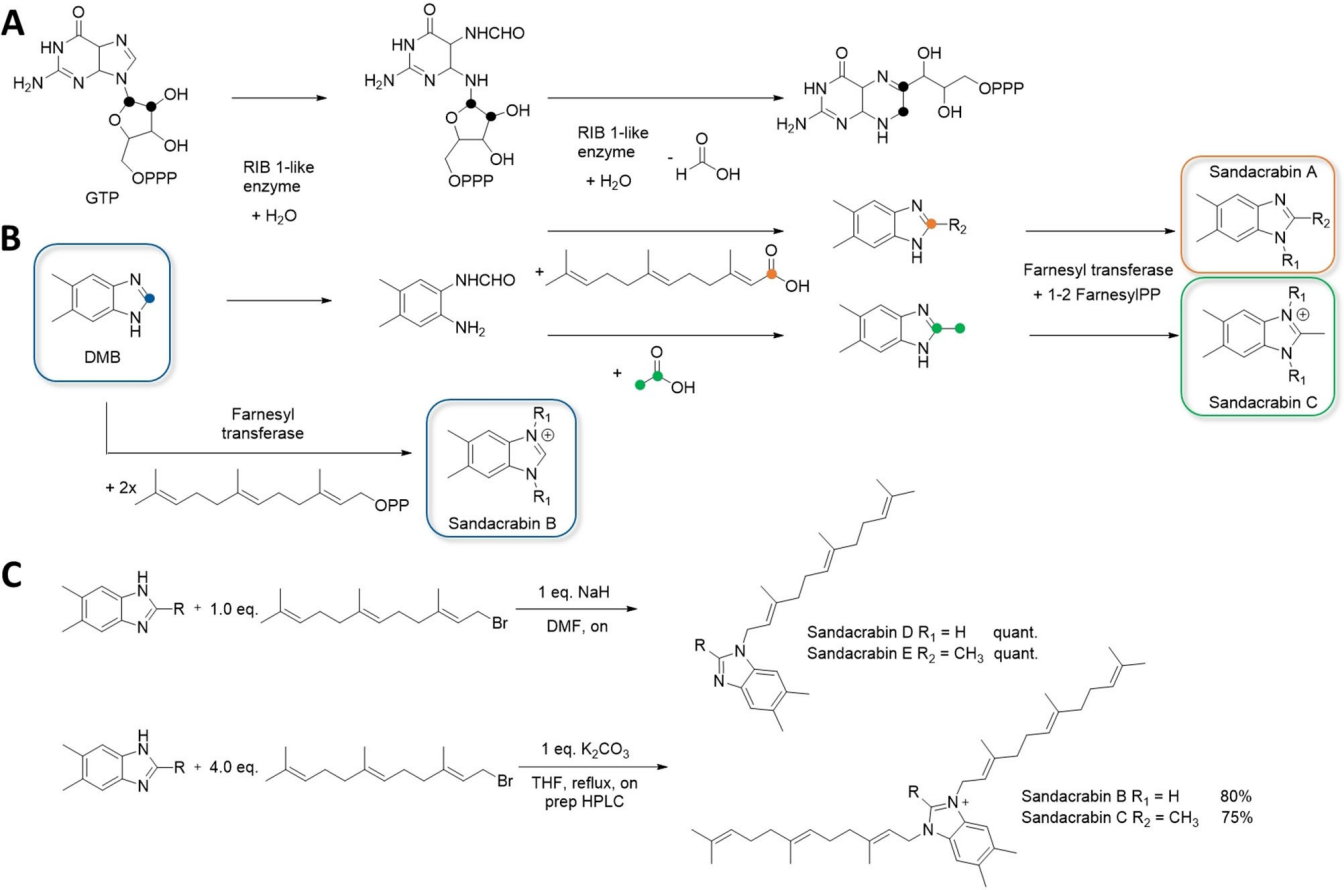
A GTP‐cyclohydrolase type reaction catalyzed by RIB 1‐like enzymes. **B** Putative sandacrabin biosynthesis starting from DMB. Carbon atoms are color‐coded highlighting their position in the respective educts and products. **C** Synthesis of sandacrabin B and C (lower part), as well as their mono‐farneslyated derivatives sandacrabin D and E (upper part).

Bacteria are also described to express a reversible condensation enzyme that fuses formate or other carboxylic acids to 1,2‐diamino‐4,5‐dimethylbenzol to form 5,6‐dimethylbenzimidazole.^[20]^ This enzyme is likely to work similarly to GTP cyclohydrolase type enzymes such as RIB1 removing a C_1_ unit bound to two vicinal nitrogen atoms as formate from GTP in a hydrolysis reaction (see Figure 4A).^[21]^ This reaction likely plays a key role in the biosynthesis of the DMB‐derived core structures of sandacrabin A and C, that may be produced by condensation of the 1,2‐diamino‐4,5‐dimethylbenzol moiety with farnesoic acid or acetic acid, respectively. To obtain their mature structure, the different DMB core structures would need to be substituted by an *N*‐farnesyl transferase that transfers one farnesyl sidechain to the precursor of sandacrabin A, while transferring two farnesyl residues to each of the two DMB ring nitrogen atoms of sandacrabin B and C (see Figure 4A).^[22]^

As the biosynthetic machinery supplying the DMB precursor was found encoded in close proximity to the cobalamin biosynthesis pathway, the complete sandacrabin biosynthetic machinery is not clustered in a single BGC as it is commonly observed in PKS or NRPS BGCs.^[23]^ Furthermore, genes encoding prenyl transferase enzymes such as the missing *N*‐farnesyl transferase are not rare in myxobacteria, and as such we were unable to pinpoint the genes responsible for sandacrabin biosynthesis.^[22]^ An overview about putative prenyl transferases detected in the *S. defensii* MSr10575 genome can be found in the Supporting Information.

### Synthesis and biological evaluation of the sandacrabins

Despite the novelty of bacterial farnesylated DMB biosynthesis, several synthetic approaches have already investigated benzimidazoles featuring various substituent patterns.^[24]^ They were found to exhibit a broad range of pharmacological activities from antimicrobial and anticancer to anthelmintic, insecticidal and anti‐inflammatory activities,^[25]^ which raised our interest in assessing the pharmacological activities of the sandacrabins as well. The isolated yields of sandacrabin B and C were comparably low, so we first aimed for a total synthesis of sandacrabin B and C (see Figure 4B) by adapting the synthesis route from the general experimental procedures for the synthesis of benzimidazoliums described by Lim et al.^[26]^ Decrease in temperature, while changing the deprotonating agent from K_2_CO_3_ to NaH during the S_N_‐type reaction also allowed us to obtain mono‐farnesylated versions called sandacrabin D and E in quantitative yields. Sandacrabin D and E were used to study the influence of the second farnesylation on the biological activities of the sandacrabins (see Figure 4B). It is worth mentioning that we detect only trace amounts of those derivatives in the extracts of MSr10575 (see Supporting Information), showing that sandacrabin biosynthesis is highly optimized for generating bis‐farnesylated sandacrabins.

Inspired by the diverse activities described for other benzimidazoles, we tested the sandacrabins activity against a broad panel of test organisms. Minimal inhibitory concentrations (MICs) for sandacrabin A−E were determined against a panel of Gram‐positive, Gram‐negative, as well as fungal human pathogens (see Table 1). Antiviral activity was assessed for the human pathogenic corona virus HCoV229E. Huh‐7.5 cells constitutively expressing a firefly luciferase reporter gene were therefore infected by a renilla luciferase HCoV229E reporter virus in the presence of indicated concentrations of the compound for 48 h at 33 °C. After lysis of cells, the renilla‐firefly luciferase dual assay allows assessment of reduction changes in viral replication, while monitoring the cell viability in parallel.^[27]^ To also assess effects on non‐infected cells, IC_50_ values against Huh 7.5 and U2‐OS were furthermore determined in an MTT‐based assay. Insecticidal activity was determined by applying 0.5 μL of an acetonic sandacrabin dilution to the posterior segment of adult *Acyrthosiphon pisum* adapted from the procedure described by Ahumada et al.^[28]^ After assessing the minimal insecticidal concentration in a small test group of three insects in a dilution series from 5–0.05 μg sandacrabin applied per insect, the death rate of 20 insects at this concentration was evaluated (see Table 1). All sandacrabins showed moderate activity against *Bacillus subtilis* and *Staphylococcus aureus* in the antimicrobial assays, with sandacrabin A exhibiting a MIC of 32 μg/mL against *B. subtilis*.

**Table 1:** Antimicrobial, insecticidal, antiviral and antiproliferative activities of the sandacrabins.

Test organism	MIC [μg/mL]					
	Sandacrabin A	Sandacrabin B	Sandacrabin C	Sandacrabin D	Sandacrabin E	**Positive control**
*Bacillus subtilis* DSM‐10	32	64	128	64	64	0.1–0.2 (Vancomycin)
	**IC_50_ [μM]**					**IC_50_ [nM]**
HCoV229E	>10	0.18	0.34	1.64	1.91	5.6 (Remdesivir)
SARS‐CoV‐2 RdRp complex	108	3.5	6.8	85.0	43.0	14 (Remdesivir)
Huh 7.5 cells	>37	0.70	3.61	4.63	6.72	37 (Doxorubicin)
U‐2 OS cells	>37	0.51	1.22	16.90	13.70	15 (Doxorubicin)
	**Death rate at 0.5 μg/insect [%]**					
*Acyrthosiphon pisum*	10	100	90	100	90	100 (Imidacloprid)

All sandacrabins except for sandacrabin A‐which did not show insecticidal activity up to 5 μg/insect‐were found to be toxic to *A. pisum* at 0.5 μg/insect. Sandacrabin B and D displayed a 100 % death rate, whereas sandacrabin C and E both showed a 90 % death rate at this concentration. The simultaneous cytotoxic effects on Huh‐7.5 cells, however, most likely impede insecticidal use of the sandacrabins.

**Figure 5:**
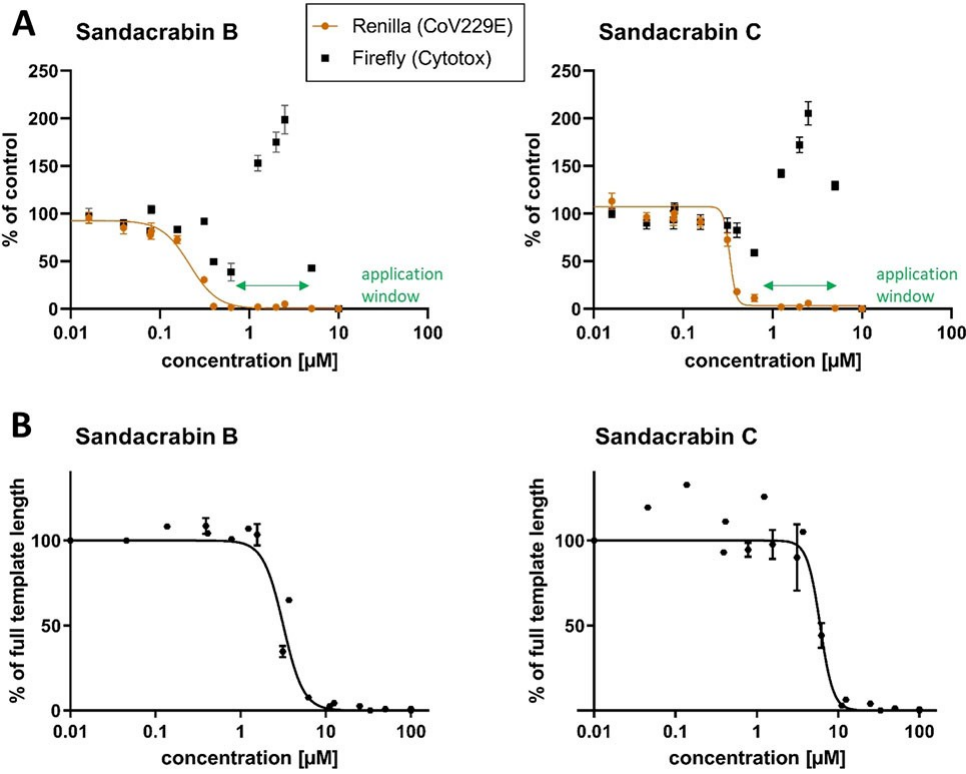
A Antiviral activities of Sandacrabin B and C against HCoV229E displayed as reduction in viral replication (orange) with simultaneous determination of the cell viability of Huh‐7.5 host cells (black). Mean values and standard deviation of triplicate measurements normalized to solvent control are given. Application window between cytotoxicity and antiviral activity as is marked in green. **B** Sandacrabin‐dependent inhibition of RNA synthesis catalyzed by SARS‐CoV‐2 RdRp complex represented as reduction in RNA synthesis products corresponding to the full template length. Respective curves for the positive controls can be found in the supporting information.

Most interestingly, we observed a significant reduction in viral replication when cells infected with the human pathogenic coronavirus HCoV229E were treated with sandacrabin B−E (see Table 1). As observed for the insecticidal activities, sandacrabin A did not show any effect on viral replication at the concentrations tested. Although sandacrabin B and C exhibited cytotoxic effects at the highest test concentration, a potential application window for development of antiviral pharmaceuticals is present (Figure 5 and Table 1). Due to the reduction in viral load, we even observe an increase in cell viability for cells treated with concentrations higher than 1 μM of sandacrabin B and C. At the two highest concentrations tested, cell viability is again reduced indicating that at this point the cytotoxic effects predominate the advantage of viral inhibition. Notably, the cytotoxic activity and antiviral reduction differ by a factor of 11 when comparing IC_50_ values against HCoV229E and Huh‐7.5 for sandacrabin C. For sandacrabin B, D and E we observe a smaller window represented by only a 3–4‐fold difference in cytotoxic and antiviral activities (see Figure 5 and Supporting Information). Interestingly, the cytotoxic effects of the monofarnesylated sandacrabins D and E are less prominent against U2‐OS cells than Huh‐7.5 cells, whereas they are slightly increased for congener B and C.

Driven by the intriguing antiviral activities of the sandacrabins, we moved on investigating their mode of action by analyzing their potential as inhibitors of viral RNA synthesis using the purified SARS‐CoV‐2 RNA‐dependent RNA polymerase complex consisting of the proteins Nsp7, Nsp8, and Nsp12 (RdRp). The results of this assay^[29]^ are depicted in Figure 5 and Figure S13. While all five sandacrabins inhibited RNA synthesis in vitro, sandacrabins B and C exhibited most promising IC_50_ values in the lower micromolar range with 3.5 μM and 6.8 μM, respectively (see Table 1). As visualized in Figure 5, inhibition of the RNA synthesis nicely correlates with the antiviral activity, whereby interaction with the RdRp complex likely represents at least part of the sandacrabin antiviral mode of action.

## Conclusion

With this study we extend the myxobacterial *Sandaracinus* family by its second member, *S. defensii* MSr10575 and report its genome sequence featuring 21 BGCs predicted by AntiSmash.^[1]^ Analysis of the strain's metabolome revealed a group of alkaloid terpenoids which we named sandacrabins. Their isolation and structure elucidation prove them as farnesylated DMBs belonging to the family of alkaloid terpenoids. To the best of our knowledge, the sandacrabins represent the first prenylated DMBs of bacterial origin, for which we developed a concise biosynthesis hypothesis. Analysis of the potential genes involved, revealed that sandacrabin biosynthesis likely consists of two steps: generation of the different DMB core structures and subsequent farnesylation by a prenyltransferase. Cobalamin biosynthesis also requires the synthesis of DMB, wherefore the biosynthesis pathway that supplies DMB to Vitamin B12 biosynthesis likely also supplies the sandacrabin biosynthesis with the respective DMB precursors. Removal of the DMB C_1_, bound to the two vicinal nitrogen atoms, would allow subsequent generation of the two sandacrabin derivatives A and C, whereas sandacrabin B incorporates the native DMB. Besides the genes involved in cobalamin biosynthesis, we could identify several genes encoding for prenyltransferases in the *S. defensii* MSr10575, supporting our biosynthesis hypothesis.

Substituted benzimidazoles have already been studied extensively in synthetic chemistry approaches towards their antimicrobial, antiviral, and insecticidal activities, which we could also observe for the sandacrabins. Highest structural similarity to the sandacrabins, which are described in this study, was found with synthetic mono‐geranylated 5,6‐dimethylbenzimidazole and mono‐farnesylated 1‐methylbenzimidazole derivatives, studied in a *Tribolium* chitin synthetase inhibition assay for generation of novel insecticidal compounds.^[30]^ Bis‐terpenylated benzimidazoles however, are rarely found in literature. One bis‐prenylated benzimidazole was generated by Holtgrewe et al. as an intermediate for studying the rearrangement of electron‐rich *N*‐allyldibenzotetraazafulvalenes. However, instead of DMB, it incorporates benzimidazole and its biological activities were not evaluated.^[31]^

Driven by the intriguing biological activities described for synthetic benzimidazole derivatives, we developed a chemical synthesis route for sandacrabin B and C produced in *S. defensii* in relatively low yields, allowing full characterization of their biological activities. We furthermore synthesized the mono‐farnesylated congeners sandacrabin D and E, which could only be detected in trace amounts in *S. defensii*. MSr10575 crude extracts. The observed broad‐spectrum activities of the different sandacrabins derivatives, which exhibit antibiotic, insecticidal and antiviral effects generally point towards a defensive function of sandacrabins for *S. defensii* MSr10575. The insecticidal activity against *A. pisum* alongside their straightforward synthesis indicates sandacrabins B−E as potential candidates for agricultural use, as their production could easily be upscaled. The detected reduction in viral replication of the human pathogenic coronavirus HCoV229E by sandacrabin B−E (Table 1) is one more example of a benzimidazole exhibiting antiviral activities, much needed in times of evolving viral pandemics such as the covid‐19 pandemic. Exemplified by remdesivir, the RdRp complex has already proven an excellent target for the treatment of SARS‐CoV2 infections.^[32]^ However, structural diversity of non‐nucleoside analogues targeting RdRp is low, having most of the few examples of this group suffer from poor pharmacokinetic properties. Furthermore, they do not bind to the active site of the complex, but rather to outer regions of the protein.^[33]^ Inhibition of the RdRp by the sandacrabins therefore marks an interesting starting point for developing non‐nucleoside analogue inhibitors addressing this intriguing target. Further studies might explore the exact binding site of the sandacrabins, as well as their pharmacokinetic properties and define the structural space where inhibition is detected.

As we observe cytotoxic effects at the highest concentration tested, medicinal chemistry optimization is certainly required to advance the sandacrabins as antiviral drug candidates. As sandacrabin A, contrary to the other derivatives, exhibits focused activity against *B. subtilis* without showing insecticidal or antiviral activity, modifications in the DMB substitution pattern (particularly at position 2) are of special interest for modifying the observed activities. The broad biological activities, which interestingly are different for the various sandacrabin derivatives, once more highlight the intriguing biosynthetic potential of rare myxobacteria and their suitability to isolate novel natural products for supplying the drug discovery pipeline.

## Conflict of interest

The authors declare no conflict of interest.

1

## Supporting information

As a service to our authors and readers, this journal provides supporting information supplied by the authors. Such materials are peer reviewed and may be re‐organized for online delivery, but are not copy‐edited or typeset. Technical support issues arising from supporting information (other than missing files) should be addressed to the authors.

Supporting Information

## Data Availability

The data that support the findings of this study are available in the supplementary material of this article.
