# Supplementary material for: Sandacrabins – Structurally Unique Antiviral RNA Polymerase Inhibitors from a Rare Myxobacterium
Source: Chemistry. 2022 Jan 22;28(10):e202104484. doi: 10.1002/chem.202104484 (PMC9306752; doi:10.1002/chem.202104484)
Supplement: Supplementary file 1 — Supporting Information [file CHEM-28-0-s001.pdf]

Chemistry–A European Journal

Supporting Information

Sandacrabins – Structurally Unique Antiviral RNA Polymerase Inhibitors from a Rare Myxobacterium

Chantal D. Bader, Fabian Panter, Ronald Garcia, Egor P. Tchesnokov, Sibylle Haid, Christine Walt, Cathrin Spröer, Alexander F. Kiefer, Matthias Götte, Jörg Overmann, Thomas Pietschmann, and Rolf Müller*

Table of Contents

S1 Cultivation conditions and extract generation 3

S1.1 Production and seed cultures 3

S1.2 Extraction 3

S1.3 Media recipes 4

S2 Analytical methods 5

S2.1 UHPLC-*hr*MS measurements 5

S2.2 MS^n measurements 5

S2.3 NMR measurements 5

S3 Isolation procedure 6

S3.1 Centrifugal partition chromatography (CPC) 6

S3.2 Supercritical fluid chromatography (SFC) 6

S3.3 Semi-preparative HPLC 6

S4 Strain and Genome analysis 7

S4.1 Morphology, Phylogenetic classification and Species Identification 7

S4.2 Genome sequencing 8

S4.3 *In-silico* analysis of the biosynthesis genes 8

S5 Structure elucidation of the sandacrabins 10

S5.1 Full description of the *de-novo* structure elucidaton 10

S5.2 NMR spectroscopic, *hr*ESI-MS and *hr*ESI-MS^3 data of the natural sandacrabins 12

S6 Chemical synthesis of the sandacrabins 18

S6.1 General experimental procedures 18

S7 Biological assays 25

S7.1 MTT assay 25

S7.2 Antimicrobial activities 25

S7.3 Antiviral activity 26

S7.4 Induction of the lysosomal apoptosis pathway 28

S7.5 Insecticidal activity 29

S7.6 RdRp assay 29

S8 NMR spectra 33

S9 References 62

S1 Cultivation conditions and extract generation

S1.1 Production and seed cultures

For fermentative production of the sandacrabins, *Sandaracinus* sp. MSr10575 was cultivated in a 50 ml pre-culture using a 300 ml shake flask containing 50 ml of 2SWT medium for 8 days at 30°C and 160 rpm. Sandacrabin production was achieved by fermentation of 6 x 5L shake flasks containing each 2L of 2SWYT medium and 40 ml of a 50% v/v sterilized XAD-16 resin. Each of these flasks was inoculated with one of the 50ml pre-cultures and fermented at 30°C and 160 rpm on an orbitron shaker for 2 weeks until the fermentation broth turns brightly orange.

S1.2 Extraction

Cells and XAD-16 resin were harvested by centrifugation and the residual medium was discarded. The XAD-16 cell mixture was repeatedly extracted using 50/50 mixtures of methanol/chloroform that were combined. The chloroform was evaporated under reduced pressure and the extract partitioned between methanol and hexane. Both fractions were dried under vacuum. The dry methanol layer was subsequently partitioned between chloroform and water and the chloroform phase was again dried again. The dried hexane layer was re-dissolved in methanol and used for the purification of sandacrabin A by semi-preparative reverse phase HPLC. The dried chloroform layer containing sandacrabin B and C was subjected to CPC separation on a Gilson CPC 100 SCPC system using a variation of the ARIZONA solvent system containing a 1:1:1:1 amount of TRIS buffer pH8, methanol, ethyl acetate and hexane. The lighter, more hydrophobic organic layer elutes a pre-purified fraction containing the sandacrabins.

S1.3 Media recipes

Table S1 Medium recipe of 2SWT medium

	**2SWT – Medium**		
Amount	Ingredient	Concentration	Supplier
3 g/L	Tryptone	-	BD
1 g/L	Soytone	-	BD
3.5 g/L	Soluble Starch	-	Roth
4 g/L	Maltose Monohydrate	-	
2 g/L	Glucose	-	Roth
10 g/L	Starch (soluble)	-	Roth
0.5 g/L	$CaCl_2$	-	Sigma Aldrich
1 g/L	$MgSO_4$ x $7H_2O$	-	Grüssing
10 ml/L	TRIS x HCl ph8	1M	Sigma Aldrich
100 µL/L	Sterile Vit. B12 solution (added after autoclaving)	1 mg/ml	Roth
200 µL/L	Sterile FeEDTA solution (added after autoclaving)	8 mg/ml	Sigma Aldrich
	Dissolved in milli-Q. Water, pH adjusted to 7.2 with 1N KOH		

Table S2 Medium recipe of 2SWYT medium.

	**2SWYT – Medium**		
Amount	Ingredient	Concentration	Supplier
3 g/L	Tryptone	-	BD
1 g/L	Soytone	-	BD
3.5 g/L	Soluble Starch	-	Roth
4 g/L	Maltose Monohydrate	-	
10 g/L	Baker's yeast (alive)	-	
2 g/L	Glucose	-	Roth
10 g/L	Starch (soluble)	-	Roth
0.5 g/L	$CaCl_2$	-	Sigma Aldrich
1 g/L	$MgSO_4$ x $7H_2O$	-	Grüssing
10 ml/L	TRIS x HCl ph8	1M	Sigma Aldrich
100 µL/L	Sterile Vit. B12 solution (added after autoclaving)	1 mg/ml	Roth
200 µL/L	Sterile FeEDTA solution (added after autoclaving)	8 mg/ml	Sigma Aldrich
	Dissolved in milli-Q Water, pH adjusted to 7.2 with 1N KOH		

S2 Analytical methods

S2.1 UHPLC-*hr*MS measurements

UHPLC-qTOF measurements were carried out on a Dionex Ultimate 3000 SL system using a Bruker maXis 4G UHRqTOF for mass spectrometric analysis. The mobile phase consisted of (A) ddH2O with 0.1% formic acid and (B) acetonitrile with 0.1% formic acid. A linear gradient from 95-5% A in B at a flow rate of 0.6 mL/min was used. Dionex Ultimate 3000 SL system was equipped with a Waters Acquity BEH C18 column (100 x 2.1 mm, 1.7 µm dp). The column was heated to 45 °C and the sample injection volume 1 µL. The LC flow was split to 75 µL/min before entering the mass spectrometer, which was externally calibrated to a mass accuracy of < 1 ppm on sodium formate clusters. Mass spectra in a mass range from 150-2500 m/z were acquired in centroid mode at a 2 Hz scan rate. Measurements were conducted in positive ionization mode, using a capillary voltage of + 4000 V. Dry gas was set to a flow rate of 5 L/min at 200 °C.

S2.2 MS^n measurements

MS^2 and MS^3 experiments are carried out in positive ionization mode on a SolariX XR 7T FT-ICR (Bruker Daltonics, Bremen, Germany) equipped with an Apollo II ESI source. The mass spectrometer was externally calibrated to a mass accuracy below 1 ppm by injecting the LC/MS Calibration standard for ESI-TOF (Agilent). The samples were injected with a preinstalled syringe pump at 2.0 µl min^{-1} flowrate. 16 scans were performed accumulating for 50 ms. The data size was set to 2 M. The capillary voltage was set to -4500 V and the dry gas to a flow rate of 4 L/min at 220 °C. CID energy is set to 22 eV for sandacrabin A and 26 eV for sandacrabin B and C. SORI energy for the second fragmentation step after isolation of the generated fragment precursor ions is set to 1.29 for sandacrabin A, 1.40 for sandacrabin B and 1.26 for sandacrabin C.

S2.3 NMR measurements

NMR spectra were recorded on a Bruker UltraShield 500 or a Bruker Ascend 700 spectrometer equipped with a 5 mm TCI cryoprobe (^{1}H at 500 and 700 MHz, ^{13}C at 125 and 175 MHz). All observed chemical shift values (δ) are given in in ppm and coupling constant values (J) in Hz. Standard pulse programs were used for HMBC, HSQC, and gCOSY experiments. HSQC experiments were optimized for ^1JC-H = 145 Hz and HMBC experiments were optimized for $^{2,3}J$C-H = 6 Hz. The spectra were recorded in methanol-d_4 and chemical shifts of the solvent signals at δ_H 3.31 ppm and δ_C 49.2 ppm were used as reference signals for spectra calibration. To increase sensitivity, the measurements were conducted in a 5 mm Shigemi tube (Shigemi Inc., Allison Park, PA 15101, USA).

S3 Isolation procedure

Separation of sandacrabin B and C from the respective CPC fractions was achieved by supercritical fluid chromatography (SFC) prior to isolation of the secondary metabolites by semi-preparative reverse phase (rp) HPLC. Sandacrabin A was directly purified from the hexane extracts using semi-preparative HPLC.

S3.1 Centrifugal partition chromatography (CPC)

Pre-purification and fractionation of the different sandacrabins into four fractions is achieved on a Gilson CPC 100 device (Gilson purification S.A.S.) connected to a Varian ProStar Solvent delivery module and a Varian ProStar 2 Channel UV detector. Fraction collection is done with a Foxy Jr. autosampler (Isco). Used biphasic solvent system is the buffered ARIZONA solvent system consisting of a 1:1:1:1 mixture of 10 mM Tris x HCl buffer pH 8.0 (Sigma), methanol (analytical grade, Fluka), EtOAc (analytical grade, Fluka) and hexane (analytical grade, Fluka). After equilibration of the system and loading of the sample, the aqueous phase is used as a stationary phase for 40 min at 2 ml/min and a fraction size of 4 ml. Then the organic phase is used as a stationary phase in dual mode for 40 min at 2 ml/min and a fraction size of 4 ml. The CPC process is followed up on the UV detector at the wavelengths 220nm and 254nm. Subsequently the sandacrabins were detected in their respective fractions using our standard analytical UHPLC-*hr*MS.

S3.2 Supercritical fluid chromatography (SFC)

Sandacrabin B was separated from sandacrabin C on a Waters Prep 15 SFC System equipped with a 5 µm Torus Diol 130 Å OBD Prep Column 250 x 19 mm thermostated at 40°C. Separation was achieved using a linear gradient of 5-50 MeOH with 0.5 % triethylamine as a co-solvent over 10 minutes. The percentage of co-solvent was kept at 50 % for 1 minute before returning to 5 % in 1 minute and re-equilibration for 3 minutes. Flow rate was set to 15 mL/min and backpressure to 110 bar.

S3.3 Semi-preparative HPLC

The final purification step for all sandacrabins was performed on a Dionex Ultimate 3000 SDLC low pressure gradient system equipped with a Waters BEH 5 µm C18(2) 100 Å, LC Column 250 x 10 mm thermostated at 45 °C coupled to a Thermo Fisher Scientific ISQ™ EM single quadrupole mass spectrometer. Separation was achieved using a linear gradient from 15% (B) acetonitrile with 0.1% formic acid to 100% (B) ddH_2O with 0.1% formic acid over 20 minutes. Detection of the compounds was achieved by UV absorption at 210 nm and selected ion monitoring (SIM) at *m/z* 541.5, 555.5 and 569.5.

S4 Strain and Genome analysis

S4.1 Morphology, Phylogenetic classification and Species Identification

Sandaracinus defensii MSr10575 was found to form swarm colonies on a standard myxobacterial mineral salts agar baited with filter paper. In axenic culture, the strain sculpts yellowish-orange colonies with flare edges towards the colony margin. Hump-shaped aggregates of orange cells are sometimes formed on the colony surface. Vegetative cells are slender rod-shaped with blunted ends capable of starch degradation (see Figure 1). Repeated sub-cultivations of the farthest swarm edge allowed the purification of the MSr10575 strain.Strain MSr10575 was classified belonging to the genus *Sandaracinus* based on 16S rRNA gene phylogenetic analysis, which it shows clustering with the *Sandaracinus amylolyticus* (Figure S1.). Genome analysis of the novel strain revealed 90.61% average nucleotide identity (OrthoANI) and 68.64% DNA-DNA hybridization (DDH) with *S. amylolyticus* DSM 53668^T (GenBank accession number: CP011125). Since it shows an identity value below the cut-off for both orthoANI (95%) and DDH (70%),[1] this suggests that it represents a new species, which is designated here as *Sandaracinus defensii*. The valid description of this species will be formally described in a separate taxonomic manuscript.

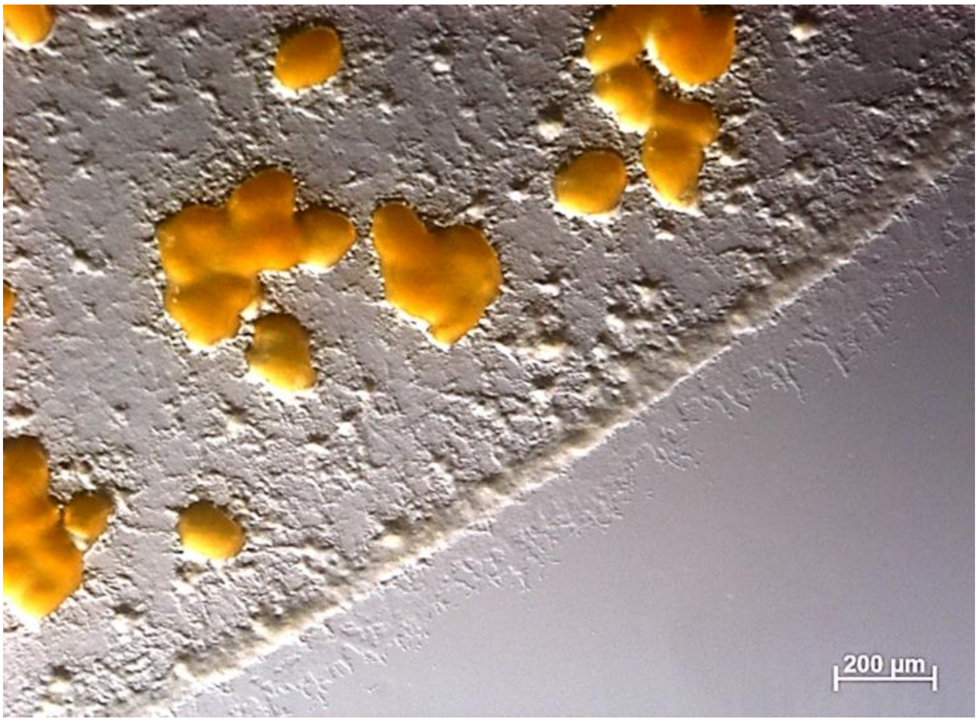

Figure S1 Growth morphology of MSr10575 on agar plates.

S4.2 Genome sequencing

The SMRTbell™ template library was prepared according to the instructions from PacificBiosciences, Menlo Park, CA, USA, following the Procedure & Checklist - *20 kb Template Preparation Using BluePippin™ Size-Selection System*. Briefly, for preparation of 15kb libraries 8µg genomic DNA was sheared using g-tubes™ from Covaris, Woburn, MA, USA according to the manufacturer´s instructions. DNA was end-repaired and ligated overnight to hairpin adapters applying components from the DNA/Polymerase Binding Kit P6 from Pacific Biosciences. Reactions were carried out according to the instructions of the manufacturer. BluePippin™ Size-Selection was performed according to the instructions of Sage Science (Beverly, MA, USA). Conditions for annealing of sequencing primers and binding of polymerase to purified SMRTbell™ template were assessed with the Calculator in RS Remote (PacificBiosciences). SMRT sequencing was carried out on the PacBio *RSII* (PacificBiosciences, Menlo Park, CA, USA) taking one 240-minutes movie on a single SMRT cell resulting in 85,167 post-filtered reads with a mean read length of 13,157 bp. Data were assembled using the “RS_HGAP_Assembly.3“ protocol included in SMRTPortal version 2.3.0 and using default parameters with the exception of the target genome size, which was increased to 13 Mbp. Three contigs were obtained from the assembly process of 10.8 Mbp, 226 kb and 60 kb. The chromosomal contig was covered 65x.

S4.3 *In-silico* analysis of the biosynthesis genes

To narrow down the biosynthetic origin of the sandacrabins we performed a global analysis of enzymes responsible for producing DMB and of farnesyl transferases in *Sandaracinus sp.* MSr10575. To support our biosynthesis hypothesis, we looked for the BluB BzaA and BzaE protein homologs that would potentially be involved in biosynthesis of the DMB core unit on the MSr10575 chromosome. Furthermore, we screened the bacterial chromosome for prenyl transferase homologs that would transform DMB into the mature sandacrabins. Locus tags described here are shown with respect to the MSr10575 chromosome deposited on GenBank under BioSample SAMN16969406 and genome Accession number CP070225 (for the bacterial chromosome) and CP070226 (for the bacterial megaplasmid). Locus tags for the Sandacrabion biosynthetic machinery are exclusively found on the bacterial chromosome under Genbank accession number CP070225.

Table S3 List of enzymes likely involved in sandacrabin biosynthesis.

Putative sandacrabin biosynthesis protein	locus-tag on the MSr10575 chromosome (CP070225)
BluB homolog 1	I5071_14990
BzaA homolog 1	I5071_83700
BzaE homolog 1	I5071_64500
BzaE homolog 2	I5071_64680
BzaE homolog 3	I5071_64650
BzaE homolog 4	I5071_54990
BzaE homolog 5	I5071_64670
Farnesyl transferase homolog 1	I5071_79930
Octaprenyl transferase homolog 1	I5071_71490
Octaprenyl transferase homolog 2	I5071_14240

It is very likely that the sandacrabin biosynthesis enzymes are among the proteins we discovered in silico. Still, as mutations in MSr10575 are difficult to achieve and we still lack the possibility to perform double crossover mutagenesis, we are unable to confirm the enzyme's involvement in the sandacrabin biosynthesis experimentally.

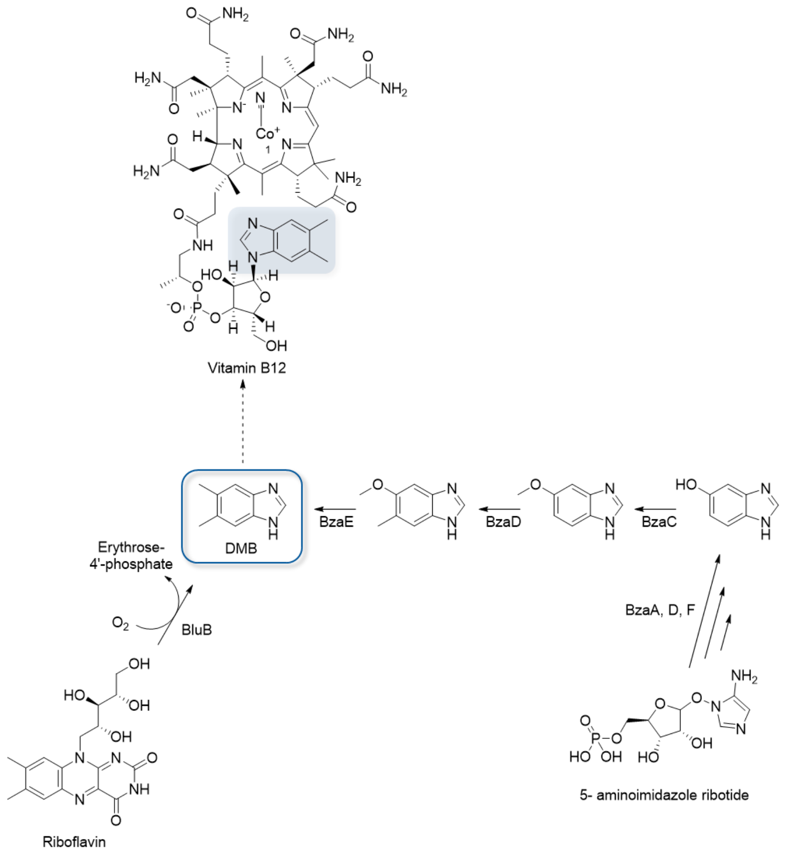

Figure S2 DMB Formation from riboflavin and 5-aminoimidazole ribotide adapted from Mattes *et al.*.

S5 Structure elucidation of the sandacrabins

S5.1 Full description of the *de-novo* structure elucidation

HRESI-MS analysis of sandacrabin A showed an $[M+H]^+$ signal at *m/z* 541.4514 (calc. 541.4516 Δ = 0.4 ppm) consistent with the sum formula of $C_{38}H_{57}N_2$ containing 12 double bond equivalents (DBEs). The ^{1}H-NMR and HSQC spectra of sandacrabin A revealed two signals characteristic for aromatic double bonds at δ(1H) = 7.37 (^{1}H, s) $\delta(^{13}C)$ = 119.6 and $\delta(^1H)$ = 7.15 (^{1}H, s) $\delta(^{13}C)$ = 111.4 ppm. Furthermore, six signals correlating to aliphatic double bond protons were detected at $\delta(^1H)$ = 6.22 (1H, s), 5.21 (1H, m), 5.16 (1H, m), 5.09 (1H, m) 5.00 (1H, m) and 4.99 ppm (1H, m). The proton spectrum exhibited 10 methyl groups at $\delta(^1H)$ = 2.38 (3H, s), 2.36 (3H, s), 2.05 (3H,m), 1.88 (3H, bs), 1.66 (3H, bs), 1.63 (3H, d, J = 1.06), 1.62 (3H, d, J = 1.06), 1.58 (3H, bs), 1.53 (3H, bs) and 1.51 ppm (3H, bs), as well as nine methylene groups at 4.76 (2H, d, J = 6.73), 2.33 (2H, m), 2.30 (2H, m), 2.11 (2H, m), 2.09 (2H, m), 2.06 (2H,m), 2.01 (2H, m), 1.91 (2H, m) and 1.82 ppm (2H, m). Based on their characteristic chemical shifts at $\delta(^{13}C)$ = 151.3, 142.2, 134.1, 133.0 and 132.3 ppm as well as HMBC correlations to the two aromatic double bond protons at $\delta(^1H)$ = 7.37 and 7.15 ppm, five quaternary carbons could be allocated in the aromatic ring system. The downfield shift of the three quaternary carbons at $\delta(^{13}C)$ 151.3, 142.2 and 134.1 ppm suggested a benzimidazole core structure of sandacrabin A. The splitting pattern of the two aromatic protons in line with COSY correlations of the methyl group at $\delta(^1H)$ = 2.36 ppm to the aromatic proton at $\delta(^1H)$ = 7.37 ppm and the methyl group at $\delta(^1H)$ = 2.38 ppm to the aromatic proton at $\delta(^1H)$ = 7.15 ppm revealed a 5,6 dimethyl substitution of the benzimidazole unit. COSY and HMBC correlations showed arrangement of the remaining methylene and methyl groups in two farnesyl moieties. Based on the chemical shift of the methylene group at $\delta(^1H)$ = 4.76 ppm and its correlation to the quaternary carbon at $\delta(^{13}C)$ = 134.1 ppm, the first farnesyl side chain is connected to the 5,6 dimethyl benzimidazole (DMB) core structure at position 1. HMBC correlations of the aliphatic double bond proton at $\delta(^1H)$ = 6.22 ppm, as well as its downfield shift showed, that the second farnesyl side chain substitutes the 2-position of the benzimidazole core structure. The high-field chemical shift of all methyl carbons pointed towards an all-*trans* configuration of the sandacrabins, which was confirmed by total synthesis and NMR spectra comparison for sandacrabin B and C.

HRESI-MS analysis of sandacrabin B showed a $[M]^+$ signal at *m/z* 555.4674 (calc. 555.4673 Δ = 0.2 ppm) consistent with the sum formula of $C_{39}H_{59}N_2$ containing 12 DBEs like sandacrabin A. 1D and 2D NMR-spectra of sandacrabin B, revealed a C2v-symmetry of the molecule (see Figure 3). Characteristic chemical shifts of two aromatic protons at $\delta(^1H)$ = 7.64 (2H, s), two methyl groups at $\delta(^1H)$ = 2.48 (6H, s) and four quaternary carbons at $\delta(^{13}C)$ = 138.5 and 131.4 ppm as well as their COSY and HMBC correlations showed that sandacrabin B also contains

a DMB core structure, comparable to sandacrabin A. COSY and HMBC correlations of further eight methyl groups, six aliphatic double bond protons, as well as ten methylene groups suggested two farnesyl substituents of the DMB core structure as well. The C2v-symmetry of the molecule, as well as its permanent positive charge indicated a 1,3- substitution of the DMB unit. A carbon signal at $\delta(^{13}C)$ = 140.2 ppm showed correlations to the two starter methylene groups of the farnesyl branches at $\delta(^{1}H)$ = 5.07 ppm, revealing its allocation at the two position in the benzimidazole core structure. Tautomerization of the imidazole double bond as well as passage of the symmetry axis at this part of the molecule however, hindered a detection of the corresponding proton signal at this position. Higher yields from chemical synthesis of sandacrabin B later on allowed detection of the respective proton at $\delta(^{1}H)$ = 9.27 ppm. HRESI-MS analysis of sandacrabin C showed a $[M]^+$ signal at *m/z* 569.4825 (calc. 569.4829 Δ = 0.7 ppm) consistent with the sum formula of $C_{40}H_{61}N_2$ containing 12 DBEs like sandacrabin A and B. 1D and 2D NMR-spectra revealed a quaternary carbon at $\delta(^{13}C)$ = 150.5 ppm, as well as its substituent, a methyl group at $\delta(^{1}H)$ = 2.81 and $\delta(^{13}C)$ = 10.4 ppm. Besides the quaternary carbon at $\delta(^{13}C)$ = 140.2 ppm in sandacrabin B, the remaining carbon and proton chemical shifts were identical for the two derivatives. In line with the C2v symmetry of sandacrabin C, this observation revealed a methylation in 2-position of the 5,6 dimethyl benzimidazole core. HRESI-MS^3 of the three sandacrabins gave additional proof for their structures proposed based on the 1D and 2D NMR spectra. They all showed loss of one farnesyl side chain. For sandacrabin B an additional peak at *m/z* 147.1 corresponding to its 5,6–dimethyl benzimidazole core structure was detected in the positive ionization ESI MS^3 spectra, respectively for sandacrabin C a peak at *m/z* 161.1074 corresponding to 1,5,6–trimethyl benzimidazole.

S5.2 NMR spectroscopic, *hr*ESI-MS and *hr*ESI-MS^3 data of the natural sandacrabins

Table S4 NMR spectroscopic data for sandacrabin A in methanol-d_4 at 500/125 MHz.

#	δ ^{13}C [PPM]	δ ^{1}H [ppm], mult (*J* [Hz])	COSY	HMBC
**1**	132.4	-	-	-
**2**	125.5	5.09, m	3, 29, 30	3, 4, 29, 30
**3**	27.9	2.09, m	2, 4	1, 2, 4
**4**	41.0	2.01, m	3	2, 3, 5, 31
**5**	137.0	-	-	-
**6**	124.8	5.21, m	7, 31	4, 5, 7, 8, 31
**7**	27.4	2.30, m	6, 8, 31	5, 6, 8, 9
**8**	41.5	2.33, m	7	6, 7, 9, 10, 11, 32
**9**	136.8	-	-	-
**10**	113.2	6.22, s	-	8, 9, 11, 32
**11**	151.3	-	-	-
**12**	142.2	-	-	-
**13**	119.6	7.37, s	16, 33	15, 17, 33
**14**	132.3	-	-	-
**15**	134.0	-	-	-
**16**	111.4	7.15, s	13, 34	12, 14, 34
**17**	134.1	-	-	-
**18**	42.9	4.76, d (6.73)	19, 35	11, 17, 19, 20, 21
**19**	121.1	5.16, m	18, 35	18, 21, 35
**20**	140.4	-	-	-
**21**	40.5	2.06, m	22	19, 20, 22, 23, 35
**22**	27.1	2.11, m	21, 23, 36	20, 21, 23, 24
**23**	124.8	5.00, m	23, 36	21, 22, 25, 36
**24**	136.5	-	-	-
**25**	40.9	1.82, m	26	23, 24, 26, 27, 36
**26**	27.9	1.91, m	25, 27, 37	24, 25, 27, 28
**27**	125.6	4.99, m	26, 37, 38	25, 26, 37, 38
**28**	132.1	-	-	-
**29**	17.9	1.58, bs	2, 30	1, 2, 30
**30**	26.1	1.63, d (1.06)	2, 3, 29	1, 2, 29
**31**	16.4	1.66, bs	6, 7	4, 5, 6
**32**	19.4	2.05, m	-	8, 10
**33**	20.6	2.36, s	13	13, 14, 15
**34**	20.8	2.38, s	16	14, 15, 16
**35**	16.9	1.88, bs	18, 19	19, 20, 21
**36**	16.3	1.51, bs	22, 23	23, 24, 25
**37**	26.0	1.62, d (1.06)	26, 27, 38	27, 28, 38
**38**	17.9	1.53, bs	27, 37	27, 28, 37

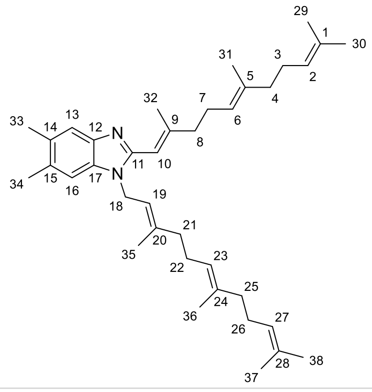

Table S5 NMR spectroscopic data for sandacrabin B in methanol-d_4 at 500/125 MHz.

#	δ ^{13}C [ppm]	δ ^{1}H [ppm], mult (*J* [Hz])	COSY	HMBC
**1, 28**	132.0	-	-	-
**2, 27**	125.2	5.01	3/26, 29/37, 30/38	3/26, 4/25, 29/37, 30/38
**3, 26**	27.6	1.95	2/27, 4/26, 29/37	1/28, 2/27, 4/25, 5/24
**4, 25**	40.6	1.85	3/26	2/27, 3/26, 5/24, 6/23, 31/38
**5, 24**	136.5	-	-	-
**6, 23**	124.5	5.05	7/22, 8/21, 31/36	4/25, 7/22, 8/21, 31/38
**7, 22**	26.8	2.18	6/23, 8/21, 31/36	6/23, 8/21, 9/20
**8, 21**	40.3	2.17	7/22	6/23, 7/22, 9/20, 10/19, 32/35
**9, 20**	145.8	-	-	-
**10, 19**	117.2	5.49	11/18, 32/35	8/21, 11/18, 32/35
**11, 18**	45.9	5.07	10/19, 32/35	9/20, 10/19, 12/17, 39
**12, 17**	131.4	-	-	-
**13, 16**	114.1	7.64	33/34	12/17, 14/15, 16/13, 33/34
**14, 15**	138.5	-	-	-
**29, 37**	25.7	1.64	2/27, 3/26, 30/38	1/28, 2/27, 30/38
**30, 38**	17.6	1.56	2/27, 29/37	1/28, 2/27, 29/37
**31, 36**	15.9	1.56	6/23, 7/22	4/25, 5/24, 6/23
**32, 35**	16.6	1.92	10/19, 11/18	8/21, 9/20, 10/19
**33, 34**	20.6	2.48	13/16	13/16, 14/15
**39**	140.2	n.d.	n.d.	n.d.

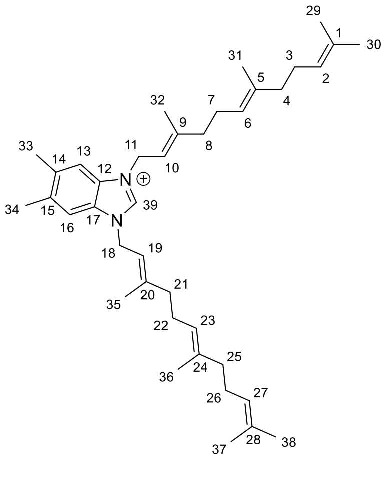

Table S6 NMR spectroscopic data for sandacrabin C in methanol-d_4 at 500/125 MHz.

#	δ ^{13}C [ppm]	δ ^{1}H [ppm], mult (J [Hz])	COSY	HMBC
**1, 28**	132.2	-	-	-
**2, 27**	125.4	5.01	3/26, 29/37, 30/38	3/26, 4/25, 29/37, 30/38
**3, 26**	27.9	1.92	2/27, 4/25, 29/37	1/28, 2/27, 4/25, 5/24
**4, 25**	41.0	1.85	3/26	2/27, 3/26, 5/25, 6/23, 31/36
**5, 24**	137.0	-	-	-
**6, 23**	124.7	5.01	7/22, 31/36	3/26, 4/25, 7/22, 8/12, 31/36
**7, 22**	27.0	2.16	6/22, 8/21, 31/36	5/24, 6/23, 8/21
**8, 21**	40.4	2.13	7/22	6/23, 7/22, 9/20, 10/19, 32/35
**9, 20**	143.9	-	-	-
**10, 19**	118.1	5.26	11/18, 32/35	8/21, 11/18, 32/35
**11, 18**	44.8	5.09	10/19, 32/35	9/20, 10/19, 12/17, 32/35, 39
**12, 17**	131.3	-	-	-
**13, 16**	113.9	7.58	33/34	12/17, 16/13, 14/15, 33/34
**14, 15**	138.0	-	-	-
**29, 37**	17.9	1.54	2/27, 3/26, 30/38	1/28, 2/27, 30/38
**30, 38**	26.0	1.64	2/27, 29/37	1/28, 2/27, 29/37
**31, 36**	16.2	1.54	6/23, 7/22	4/25, 5/24, 6/23
**32, 35**	16.9	1.95	10/19, 11/18	8/21, 9/20, 10/19, 11/18
**33, 34**	20.7	2.46	13/16	13/16, 14/15
**39**	150.5	-	-	-
**40**	10.4	2.81	-	12/17

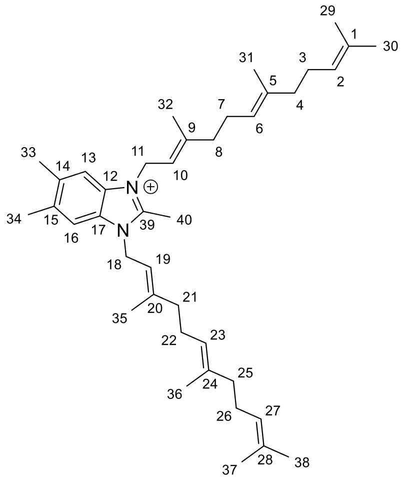

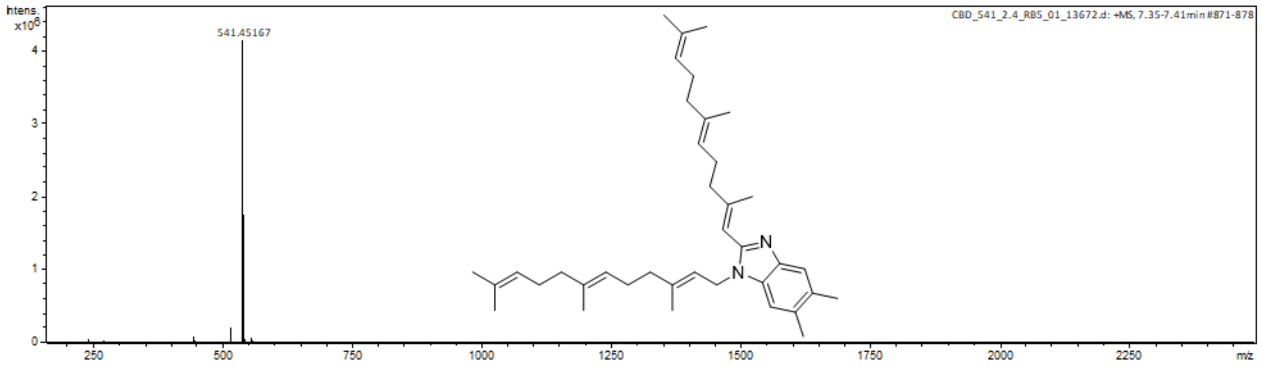

Figure S3 *hr*ESI-MS spectrum of sandacrabin A.

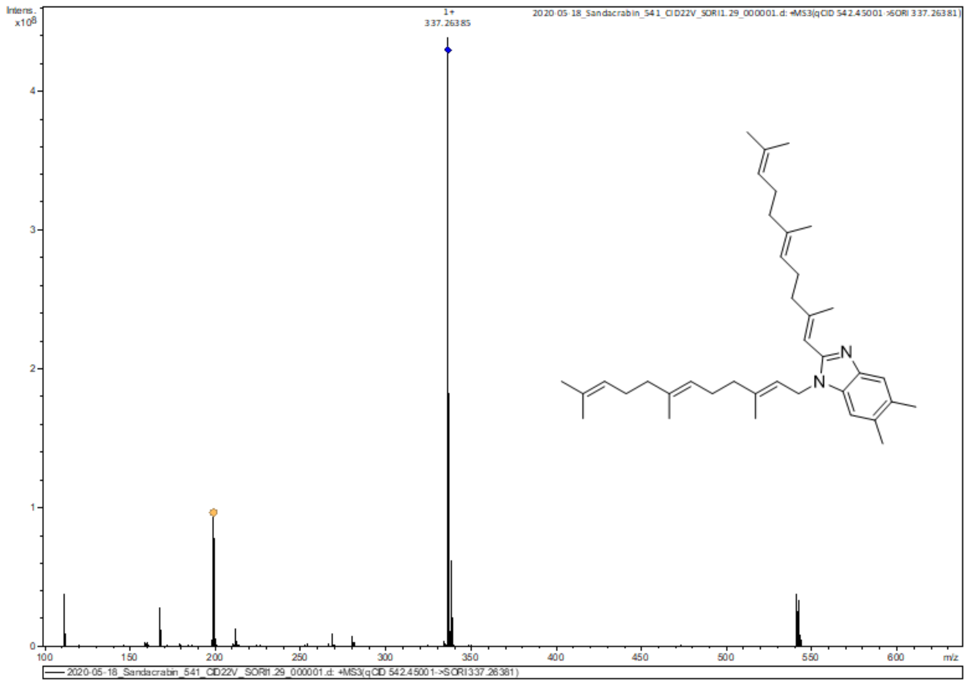

Figure S4 *hr*ESI-MS^3 spectrum of sandacrabin A.

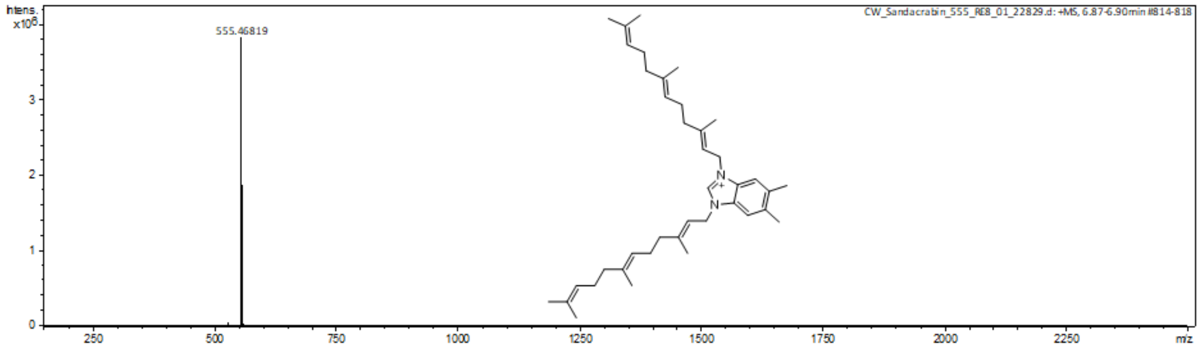

Figure S5 *hr*ESI-MS spectrum of sandacrabin B.

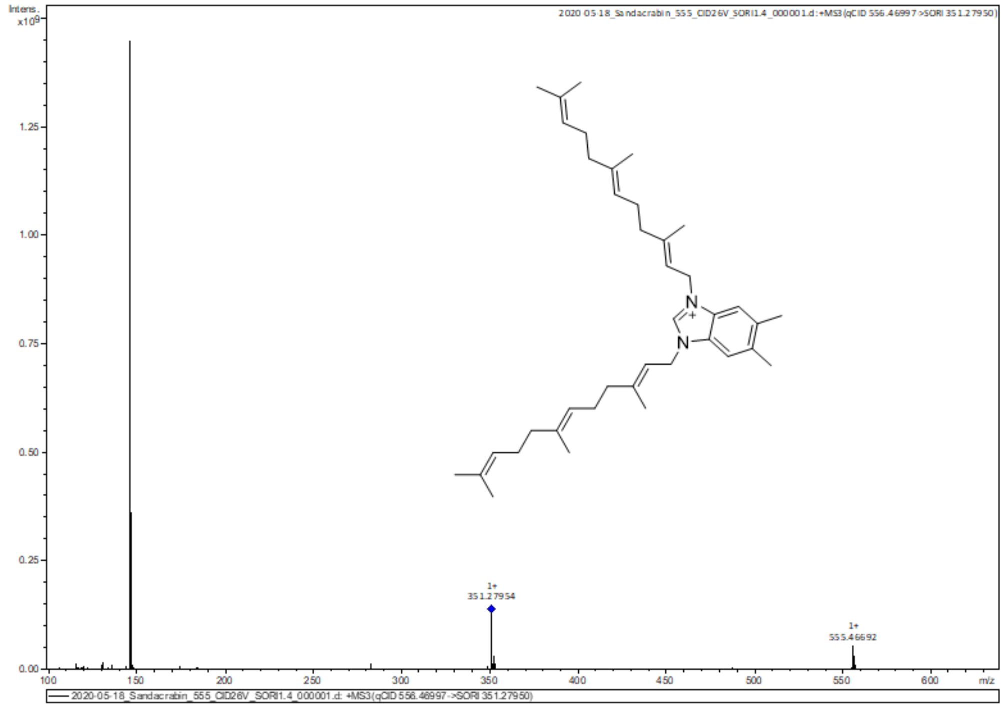

Figure S6 *hr*ESI-MS^3 spectrum of sandacrabin B.

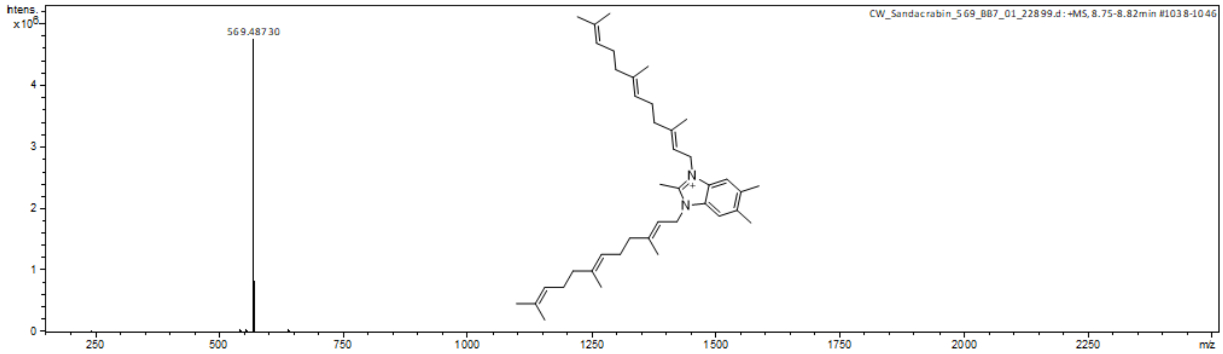

Figure S7 *hr*ESI-MS spectrum of sandacrabin C.

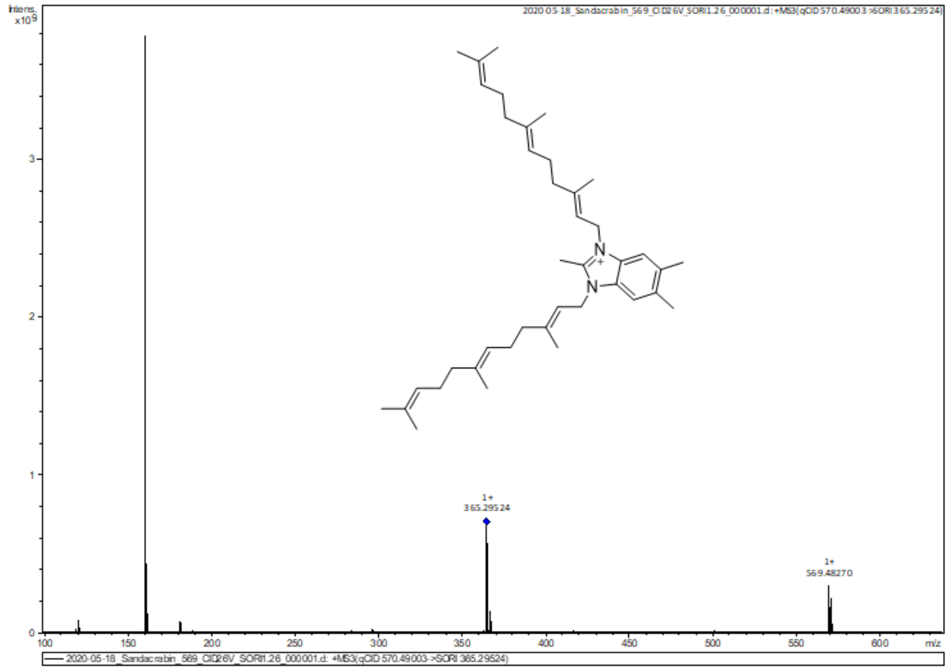

Figure S8 *hr*ESI-MS^3 spectrum of sandacrabin C.

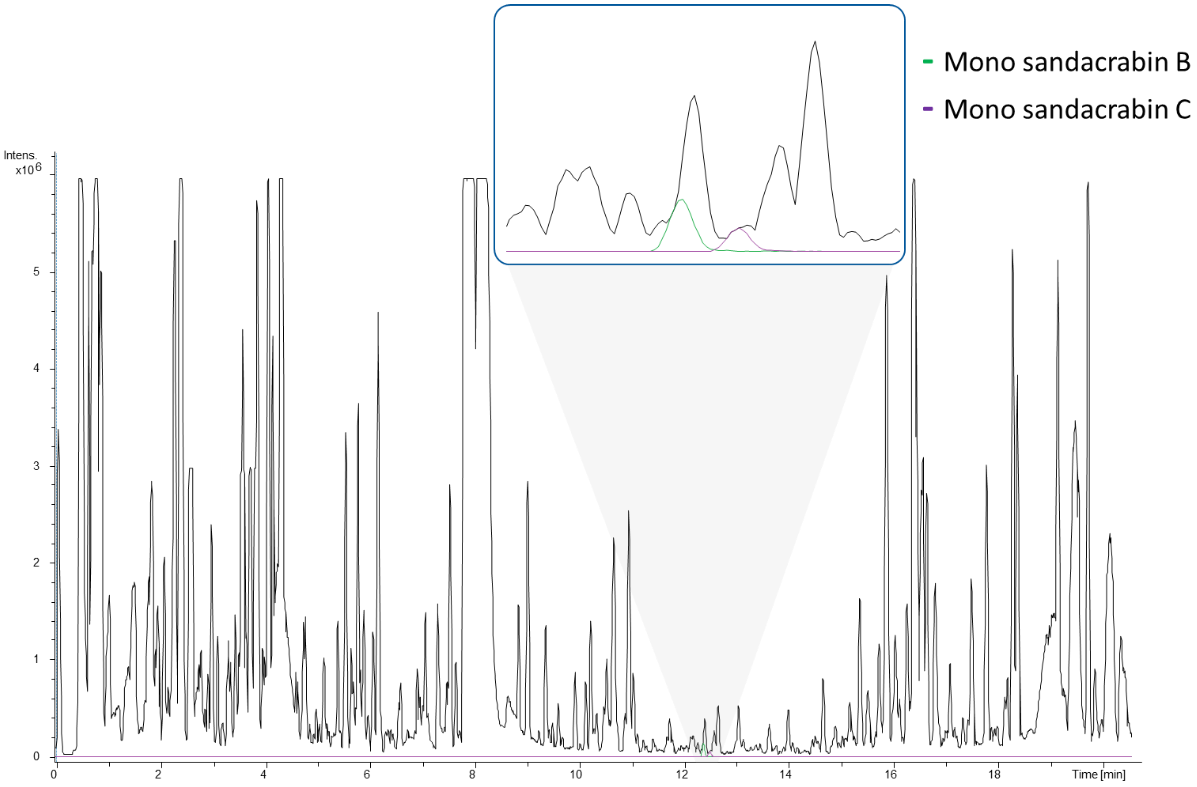

Figure S9 LC-MS analysis of MSr10575 crude extract. Base peak chromatogram (BPC, black trace), extracted ion chromatograms (EIC) for monofarnesylated sandacrabin B (green trace) at 351.2790 ±0.005 Da and monofarnesylated sandacrabin C (violet trace) at 365.2951 ±0.005 Da.

S6 Chemical synthesis of the sandacrabins

S6.1 General experimental procedures

General procedure A: monofarnesylation

Mono-farnesylation of the respective benzimidazole core structures was achieved adapted from the procedure described by Kishida *et al.*.(5) The benzimidazole (1.0 equiv) in DMF was added dropwise to a mixture of DMF and a 90% oil suspension of NaH (1.0 equiv) at room temperature. The resulting mixture was stirred for 1 h, before farnesylbromide (1.0 equiv) in DMF was added dropwise at 5-10 °C. The 0.1 M solution was stirred overnight at room temperature. After complete conversion (LC-MS), the reaction was quenched with water. The mixture was extracted with EtOAc twice, before the combined organic layers were washed with brine and dried over Na_2SO_4. Concentration of the solvent *in vacuo* afforded the desired product as light yellow oils.

General procedure B: bis-farnesylation

Bis-farneylation of the respective benzimidazole core structures was achieved adapted from the procedure described by Lim *et al.*.[2] 2 mL of THF was added to a crimp vial equipped with a magnetic stir bar followed by the addition of the benzimidazole (1 equiv), farnesylbromide (4 equiv) and finally potassium carbonate (1 equiv). This suspension was subsequently heated at 80 °C over night. After cooling down the reaction to room temperature, the solvent was evaporated under reduced pressure. The residue was dissolved in EtOAc and the mixture extracted twice with brine. The combined organic layers were dried over Na_2SO_4, the EtOAc was evaporated under reduced pressure and the residue re-dissolved in methanol before purification by semipreparative HPLC.

2,5,6-trimethyl-1H-benzo[*d*]imidazole (1)

2,5,6-trimethyl-1H-benzo[d]imidazole was synthesized like previously described by Llona-Minguez *et al.*.[3] 4,5-dimethylbenzene-1,2-diamine (1.02 g, 7.36 mmol) was added to 20 mL (73.6 mmol) acetic acid in a crimp vial equipped with a magnetic stir bar. The crimp vial was sealed and heated at 145 °C for 30 min. The reaction mixture was allowed to cool to room temperature and then poured into 40 mL of saturated sodium carbonate. The precipitate was filtered off and washed with water repeatedly. The precipitate was dried *in vacuo* giving the desired benzimidazole as light brown solid in quantitative yields.

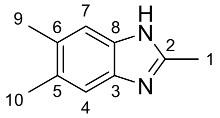

^{1}H NMR (500 MHz, DMSO-d_6): δ = 2.26 (s, 6H, 9-H, 10-H), 2.43 (s, 3H, 1-H), 7.20 (bs, 2H, 4-H,7-H), 11.96 (bs, 1H, NH)

^{13}C NMR (125 MHz, DMSO-d_6): δ = 14.6 (s, 1-C), 19.9 (s, 9-C, 10-C), 110.9 (bs, 7-C), 118.1 (bs, 4-C), 129.2 (bs, 5-C, 6-C), 133.0 (bs, 8-C), 142.2 (bs, 3-C), 150.3 (s, 2-C)

HRMS (ESI): *m/z* calculated 161.1073 for $C_{10}H_{13}N_2$; found 161.1077 Δ-0.37 ppm.

IR (neat, cm^{-1}): v = 2979, 2920, 1452, 1432, 1380, 1307, 1103, 1020, 1001, 867, 854, 806, 678, 654

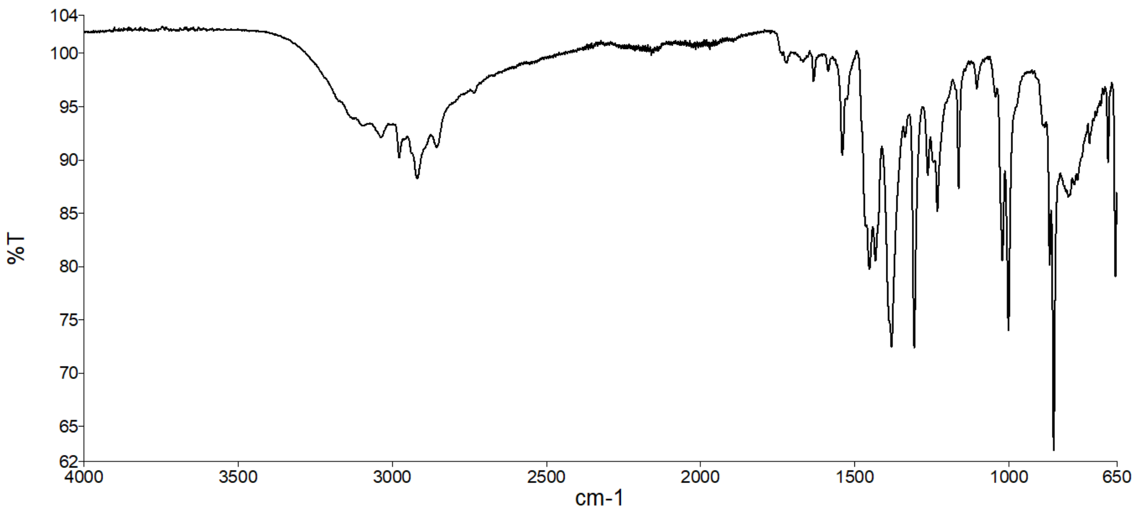

Figure S10 IR spectrum of **1**.

5,6-dimethyl-1-((2*E*,6*E*)-3,7,11-trimethyldodeca-2,6,10-trien-1-yl)-1*H*-benzo[*d*]imidazole (2)

According to **GP A**, 5,6-dimethyl-1*H*-benzo[*d*]imidazole (26.3 mg, 0.18 mmol) was reacted with farnesylbromide (50.0 mg, 0.18 mmol) utilizing NaH (4.1 mg, 0.18 mmol) in 1.8 mL DMF. The reaction resulted in 5,6-dimethyl-1-((2*E*,6*E*)-3,7,11-trimethyldodeca-2,6,10-trien-1-yl)-1*H*-benzo[*d*]imidazole (**2**) in quantitative yields as a pale yellow oil.

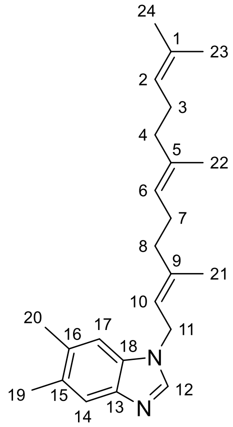

HRMS (ESI): m/z calculated 351.2795 for $C_{24}H_{35}N_2$; found 351.2795 Δ -0.01 ppm.

UHPLC (18min): Rt = 11.98 min

Table S7 NMR spectroscopic data for 2 in methanol-d_4 at 700/175 MHz.

#	δ ^{13}C [ppm]	δ ^{1}H [ppm], mult (*J* [Hz])	COSY	HMBC
**1**	132.1	-	-	-
**2**	125.6	4.99	3, 23	3, 4, 23, 24
**3**	27.8	1.93	2, 4	1, 2, 4, 5
**4**	40.9	1.84	3	2, 3, 5, 6, 22
**5**	136.6	-	-	-
**6**	124.9	5.00	7, 22	4, 7, 8, 22
**7**	27.1	2.11	6, 8	4, 5, 6, 8, 9, 22
**8**	40.5	2.07	7	6, 7, 9, 10, 21
**9**	142.4	-	-	-
**10**	120.2	5.34	11, 21	8, 11, 21
**11**	43.9	5.37	10, 21	8, 9, 10, 12, 13, 18, 21
**12**	143.4	7.93	-	13, 18
**13**	142.9	-	-	-
**14**	120.2	7.41	19	16, 18, 19
**15**	132.6	-	-	-
**16**	133.5	-	-	-
**17**	111.8	7.19	20	13, 15, 20
**18**	133.5	-	-	-
**19**	20.5	2.32	14	14, 15, 16
**20**	20.9	2.35	17	15, 16, 17
**21**	16.7	1.83	10, 11	8, 9, 10, 11
**22**	16.3	1.53	6	4, 5, 6
**23**	26.1	1.61	2	1, 2, 24
**24**	17.9	1.52	-	1, 2, 23

2,5,6-trimethyl-1-((2*E*,6*E*)-3,7,11-trimethyldodeca-2,6,10-trien-1-yl)-1*H*-benzo[*d*]imidazole (3)

According to **GP A**, 2,5,6-trimethyl-1*H*-benzo[*d*]imidazole (28.8 mg, 0.18 mmol) was reacted with farnesylbromide (50.0 mg, 0.18 mmol) utilizing NaH (4.1 mg, 0.18 mmol) in 1.8 mL DMF. The reaction resulted in 2,5,6-trimethyl-1-((2*E*,6*E*)-3,7,11-trimethyldodeca-2,6,10-trien-1-yl)-1*H*-benzo[*d*]imidazole (**3**) in quantitative yields as a pale orange oil.

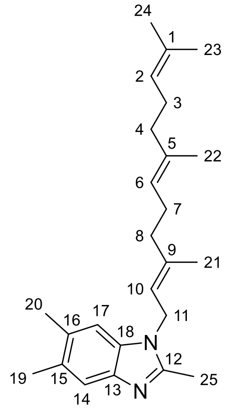

HRMS (ESI): m/z calculated 365.2951 for $C_{25}H_{37}N_2$; found 365.2957 Δ -1.45 ppm.

UHPLC (18min): Rt = 12.06 min

Table S8 NMR spectroscopic data for 3 in methanol-*d₄* at 700/175 MHz.

#	δ ^{13}C [ppm]	δ ^{1}H [ppm], mult (*J* [Hz])	COSY	HMBC
**1**	132.2	-	-	-
**2**	125.5	4.99	3, 23	3, 4, 23, 24
**3**	27.9	1.91	2, 4, 23, 24	1, 2, 4, 5
**4**	40.9	1.83	3	2, 3, 5, 6, 22
**5**	136.6	-	-	-
**6**	124.9	5.00	7, 22	4, 7, 8, 22
**7**	27.1	2.10	6, 8, 22	5, 6, 8, 9
**8**	40.5	2.06	7	6, 7, 9, 10, 21
**9**	140.9	-	-	-
**10**	120.5	5.14	11, 21	8, 11, 21
**11**	42.9	4.72	10, 21	9, 10, 12, 13, 18
**12**	152.2	-	-	-
**13**	141.3	-	-	-
**14**	119.1	7.30	19	16, 18, 19
**15**	132.1	-	-	-
**16**	132.6	-	-	-
**17**	111.3	7.12	20	13, 14, 15, 20
**18**	134.7	-	-	-
**19**	20.5	2.32	14	14, 15, 16
**20**	20.8	2.35	17	15, 16, 17
**21**	16.8	1.87	10, 11	8, 9, 10
**22**	16.3	1.52	6, 7	4, 5, 6
**23**	26.0	1.62	2, 3, 24	1, 2, 3, 24
**24**	17.9	1.53	2, 3, 23	1, 2, 23
**25**	13.6	2.51	-	

5,6-dimethyl-1,3-bis((2*E*,6*E*)-3,7,11-trimethyldodeca-2,6,10-trien-1-yl)-1*H*-benzo[*d*]imidazolium (4)

According to **GP B**, 5,6-trimethyl-1*H*-benzo[*d*]imidazole (6.6 mg, 0.05 mmol) was reacted with farnesylbromide (50.0 mg, 0.18 mmol) utilizing K_2CO_3 (6.2 mg, 0.05 mmol) in 2.0 mL THF. The reaction resulted in 5,6-dimethyl-1,3-bis((2*E*,6*E*)-3,7,11-trimethyldodeca-2,6,10-trien-1-yl)-1*H*-benzo[*d*]imidazoleium (**4**) (14.0 mg, 0.04 mmol, 80%) as a pale yellow oil.

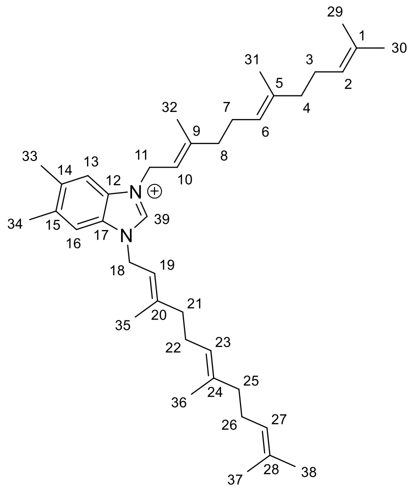

HRMS (ESI): m/z calculated 555.4673 for $C_{39}H_{59}N_2$; found 555.4669 Δ0.76ppm

UHPLC (9min): Rt = 8.76 min

Table S9 NMR spectroscopic data for 4 in methanol-*d*$_4$ at 500/125 MHz.

#	δ ^{13}C [ppm]	δ ^{1}H [ppm], mult (*J* [Hz])	COSY	HMBC
**1, 28**	132.3	-	-	-
**2, 27**	125.5	5.01	3/26, 29/37, 30/38	3/26, 4/25, 29/37, 30/38
**3, 26**	27.9	1.95	2/27, 4/26, 29/37	1/28, 2/27, 4/25, 5/24
**4, 25**	40.9	1.85	3/26	2/27, 3/26, 5/24, 6/23, 31/38
**5, 24**	136.9	-	-	-
**6, 23**	124.7	5.06	7/22, 8/21, 31/36	4/25, 7/22, 8/21, 31/38
**7, 22**	27.1	2.19	6/23, 8/21, 31/36	5/24, 6/23, 8/21, 9/20
**8, 21**	40.6	2.17	7/22	6/23, 7/22, 9/20, 10/19, 32/35
**9, 20**	146.1	-	-	-
**10, 19**	117.5	5.49	11/18, 32/35	8/21, 11/18, 32/35
**11, 18**	46.3	5.08	10/19, 32/35	9/20, 10/19, 12/17, 39
**12, 17**	131.7	-	-	-
**13, 16**	114.5	7.64	33/34	12/17, 14/15, 16/13, 33/34

**14, 15**	138.8	-	-	-
**29, 37**	26.1	1.64	2/27, 3/26, 30/38	1/28, 2/27, 30/38
**30, 38**	17.9	1.56	2/27, 29/37	1/28, 2/27, 29/37
**31, 36**	16.3	1.56	6/23, 7/22	4/25, 5/24, 6/23
**32, 35**	16.9	1.93	10/19, 11/18	8/21, 9/20, 10/19
**33, 34**	20.8	2.48	13/16	13/16, 14/15
**39**	140.9	9.27	-	-

2,5,6-trimethyl-1,3-bis((2*E*,6*E*)-3,7,11-trimethyldodeca-2,6,10-trien-1-yl)-1*H*-benzo[*d*]imidazol-3-ium (5)

According to **GP B**, 2,5,6-trimethyl-1*H*-benzo[*d*]imidazole (**1**) (7.3 mg, 0.05 mmol) was reacted with farnesylbromide (50.0 mg, 0.18 mmol) utilizing K_2CO_3 (6.2 mg, 0.05 mmol) in 2.0 mL THF. The reaction resulted in 2,5,6-dimethyl-1,3-bis((2*E*,6*E*)-3,7,11-trimethyldodeca-2,6,10-trien-1-yl)-1*H*-benzo[*d*]imidazole (**5**) (13.7 mg, 0.04 mmol, 75%) as a pale orange oil.

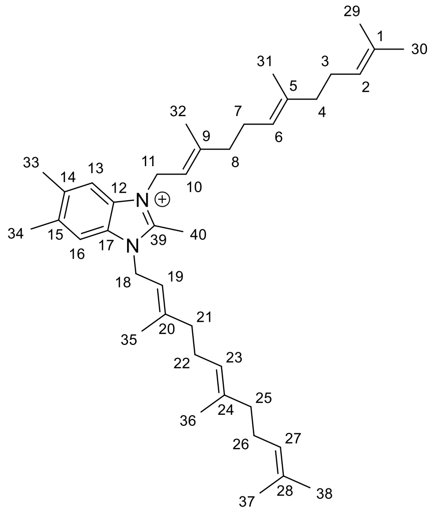

HRMS (ESI): m/z calculated 569.4829 for $C_{40}H_{61}N_2$; found 569.4822.

UHPLC (9min): Rt = 8.79 min

Table S10 NMR spectroscopic data for 5 in methanol-d_4 at 500/125 MHz.

#	δ ^{13}C [ppm]	δ ^{1}H [ppm], mult (*J* [Hz])	COSY	HMBC
**1, 28**	132.4	-	-	-
**2, 27**	125.4	5.00	3/26, 30/38	3/26, 4/25, 29/37, 30/38
**3, 26**	27.9	1.93	2/27, 4/25, 29/37, 30/38	1/28, 2/27, 4/25, 5/24
**4, 25**	40.9	1.84	3/26	2/27, 3/26, 5/25, 6/23
**5, 24**	136.8	-	-	-
**6, 23**	124.7	5.02	7/22, 31/36	4/25, 7/22, 8/12, 31/36
**7, 22**	27.0	2.14	6/22, 8/21	5/24, 6/23, 8/21, 9/20
**8, 21**	40.5	2.13	7/22, 10/19, 32/35	6/23, 7/22, 9/20, 10/19, 32/35
**9, 20**	143.9	-	-	-
**10, 19**	118.0	5.26	8/21, 11/18	8/21, 11/18, 32/35
**11, 18**	44.9	5.09	10/19, 32/35	9/20, 10/19, 12/17, 32/35, 39
**12, 17**	131.1	-	-	-
**13, 16**	113.9	7.58	33/34	12/17, 16/13, 14/15, 33/34
**14, 15**	138.0	-	-	-
**29, 37**	17.9	1.54	2/27, 3/26, 30/38	1/28, 2/27, 30/38
**30, 38**	26.1	1.64	3/26, 29/37	1/28, 2/27, 29/37
**31, 36**	16.3	1.54	6/23	4/25, 5/24, 6/23
**32, 35**	16.9	1.95	8/21, 11/18	8/21, 9/20, 10/19, 11/18
**33, 34**	20.8	2.46	13/16	13/16, 14/15
**39**	150.5	-	-	-
**40**	10.8	2.85	-	12/17, 39

S7 Biological assays

For the biological assays described in the following chapters S7.1-7.3 10 mM DMSO stock solutions of the sandacrabins were diluted with the respective buffers. We did not observe any aggregation or precipitation of the sandacrabins at the tested concentrations.

S7.1 MTT assay

U-2 human osteosarcoma cells (U-2 OS) were bought from the German Collection of Microorganisms and Cell Cultures (Deutsche Sammlung für Mikroorganismen und Zellkulturen, DSMZ) and non-infected Huh-7.5 cells were kindly provided by Twincore. Both were cultured under the conditions recommended by the depositor. To determine the cytotoxic activities of the sandacrabins, cells from actively growing cultures were harvested and seeded at 5 x 10^4 cells per well in a 96 CELLBind® surface well plate in 120 µL 90% modified McCoy's 5A medium with 10% h.i. fetal bovine serum (FBS). After 2 h of equilibration, the cells were treated with the compounds in a serial dilution. After 5 days of incubation at 37 °C, 20 µL of 5 mg/mL thiazolyl blue tetrazolium bromide (MTT) in PBS was added. After discarding the medium, 100 µL of a 2-propanol 10 N HCl mixture (250:1) was added to dissolve formazan granules. A microplate reader (EL808, Bio-Tek Instruments Inc.) was used to determine the absorbance at 570 nm. Doxorubicin was used as a positive control (see table 1).

S7.2 Antimicrobial activities

All microorganisms used for the biological assays were obtained from the German Collection of Microorganisms and Cell Cultures (Deutsche Sammlung für Mikroorganismen und Zellkulturen, DSMZ) or were part of our in-house strain collection and were cultured under the conditions recommended by the depositor. Bacterial cultures were prepared in MHB medium inoculated from the strain grown on agar plate (2.9 g/L beef infusion solids, 17.5 g/L casein hydrolysate, 1.5 g/L starch at pH 7.4).The compounds were diluted serially in sterile 96 well-plates before adding the bacterial cell suspension. The bacteria were grown for 24 h at 37 °C. Growth inhibition was inspected visually. MIC_{50} values were determined relative to the respective control samples by sigmoidal curve fitting. Activities are summarized in table S11.

Table S11. Antimicrobial activities of the sandacrabins.

Test organism	MIC [µg/mL]				
	Sandacrabin A	Sandacrabin B	Sandacrabin C	Sandacrabin D	Sandacrabin E
Bacillus subtilis DSM-10	32	64	128	64	64
Staphylococcus aureus Newman	128	64-128	64-128	64	64
Candida. albicans DSM-1665	> 64	> 64	> 64	> 64	> 64
Pichia anomala	> 64	> 64	> 64	> 64	> 64
Escherichia coli BW25113	> 64	> 64	> 64	> 64	> 64
Escherichia coli ΔacrB JW0451-2	> 64	> 64	> 64	> 64	> 64
Pseudomonas aeruginosa PA14	> 64	> 64	> 64	> 64	> 64
Mycobacterium smegmatis mc^2 155	> 64	> 64	> 64	64	64

S7.3 Antiviral activity

Huh-7.5 cells constitutively expressing a firefly luciferase reporter gene were infected with a renilla luciferase CoV229E reporter virus in the presence of indicated concentrations of the compound. 48h post inoculation, cells were lysed in PBS/0.5% Triton-X100 and infection efficiency was determined by renilla luciferase assay, whereas cell survival was analyzed by firefly luciferase assay. Data were normalized to solvent treated control. Remdesivir was used as a positive control (see table 1 and figure S12).

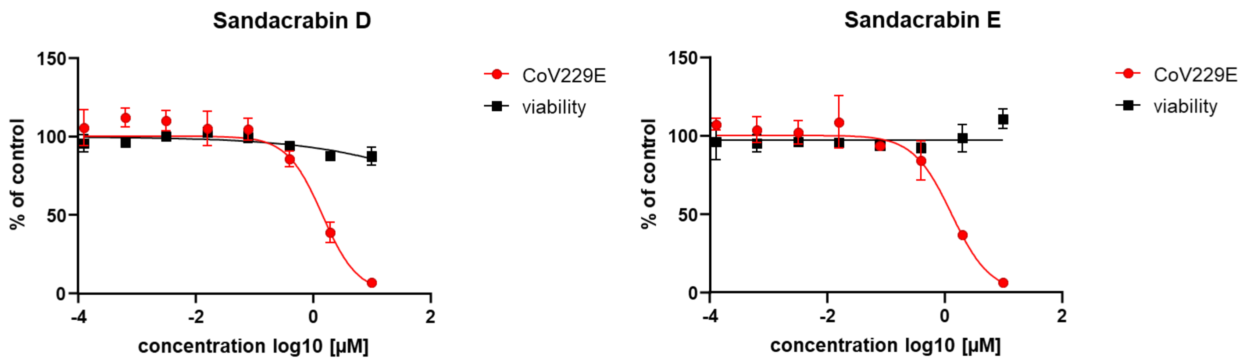

Figure S11. Antiviral activities of Sandacrabin D and E against HCoV229E displayed as reduction in viral replication (red) with simultaneous determination of the cell viability of Huh-7.5 host cells (black). Mean values and standard deviation of triplicate measurements normalized to solvent control are given. IC_{50} values against Huh-7.5 cells were determined in a separate experiment.

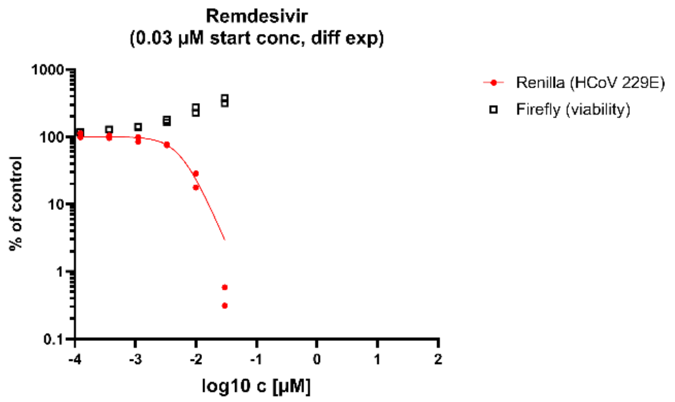

Figure S12. Antiviral activities of Remdesivir against HCoV229E displayed as reduction in viral replication (red) with simultaneous determination of the cell viability of Huh-7.5 host cells (black). Mean values and standard deviation of triplicate measurements normalized to solvent control are given.

S7.4 Induction of the lysosomal apoptosis pathway

Lysosomal apoptosis induction was determined by DNA ladder assay adapted from Saadat *et al.*.[4] Human HCT-116 colon carcinoma cells (ACC-581) were cultivated in a 6 well plate containing 2 mL 90% modified McCoy's 5A medium with 10% h.i. fetal bovine serum (FBS) each. After 48 h of equilibration, the cells were treated with the compounds in MeOH at 2 x MIC. As negative control pure MeOH and as positive control 500 µM H_2O_2 was used. After 24h incubation at 37 °C, floating apoptotic cells were harvested by pipetting the supernatant into tubes and centrifuging the supernatant at 5000 rpm for 5 minutes. The supernatant was discarded and the empty wells containing adherent cells were incubated with 500 µL lysis buffer. After 10 minutes of incubation at room temperature, the lysate cells from the wells were pipetted into the respective tubes. The combined cells in lysis buffer were incubated at 65 °C for 5 minutes. After cooling to room temperature, 700 µL chloroform-isoamyl alcohol was added and centrifuged at 12000 rpm for 5 minutes. The aquatic upper phase was transferred into fresh tubes and 100 µL of 3.9M $(NH_4)_2SO_4$solution was added to precipitate residual protein and centrifuged at 12000 rpm for 5 minutes. The pellet is discarded and 800µL of Isopropanol at -20°C were added to the supernatant in a fresh tube to precipitate the DNA. The tubes were inverted gently before another centrifugation step at 12000 rpm for 5 minutes. The supernatant was discarded and the pellet air-dried for 30 minutes. The dry DNA was dissolved in 50 µL distilled water. The samples were separated by electrophoresis on a 1.5% agarose gel containing 1 µL/100 mL Roti-Safe DNA gel stain (Roth). The gel was examined by ultraviolet gel documentation.

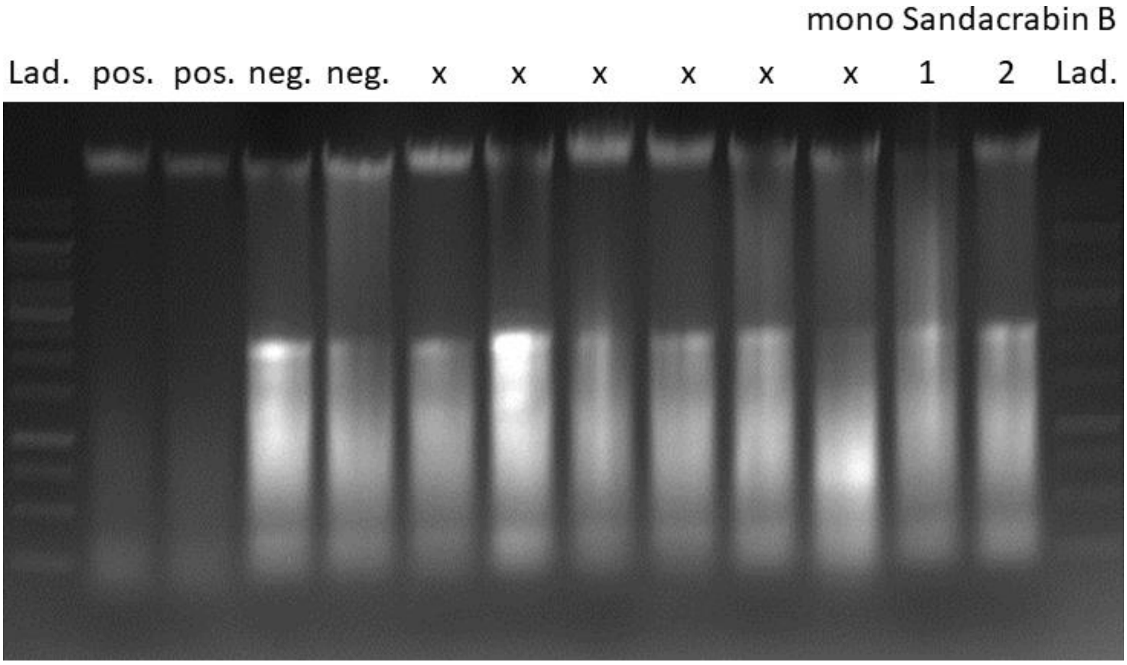

Figure S13. Gel documentation picture of the apoptosis assay, pos indicating H_2O_2 positive control, neg indicating negative control, x belonging to assays not related to sandacrabins that were not cropped to show integrity of the agarose gel and lanes 1 and 2 belonging to mono sandacrabin B. Lad refers to the 1kb DNA ladder purchased from Thermo scientific.

S7.5 Insecticidal activity

Acyrthosiphon pisum were obtained from DendroShop Terraristik and maintained as non-treated culture on pea sprouts prior to retrieval for the insecticidal assays. Insecticidal activity against *A. pisum* was determined as direct spray insecticidal activity adapted from the procedure described by Ahumanda *et al.*.[5] 0.5 microliter of insecticide solution in acetone were applied with a transfer pipette (BRAND, Germany) directly onto the posterior segment of the adult *A. pisum* in a petri dish. As negative control pure acetone and as positive control 0.01 µg/mL imidacloprid (PESTANAL®, Sigma-Aldrich) in acetone was used. Twenty replicates were tested for each insecticide. The exposed insects were maintained at 20 °C until evaluation of their survival via tactile stimulation after one hour. The acetone control group showed a death rate of 10%, whereas the group treated with imidacloprid showed a 100% death rate.

S7.6 RdRp assay

Protein expression and purification

SARS–CoV-2 RdRp complex (nsp7, -8, and -12) was expressed and purified as we reported previously.[6] The pFastBac-1 (Invitrogen, Burlington, Ontario, Canada) plasmid with the codon-optimized synthetic DNA sequences (GenScript, Piscataway, NJ) coding for a portion of 1ab polyprotein of SARS-CoV-2 (NCBI: QHD43415.1) containing only nsp5, nsp7, nsp8, and nsp12 was used as a starting material for protein expression in insect cells (Sf9, Invitrogen). We employed the MultiBac (Geneva Biotech, Geneva, Switzerland) system for protein expression in insect cells (Sf9, Invitrogen) according to published protocols.[7] SARS–CoV-2 RdRp complex was purified using Ni-NTA affinity chromatography of the nsp8 N-terminal eight-histidine tag according to the manufacturer's specifications (Thermo Scientific, Rockford, IL, USA).

RNA synthesis inhibition assay

RNA synthesis inhibition assay consisted of pre-incubating 300 nM of SARS-CoV-2 RdRp complex with either 100 µM (screening experiments) or serial dilutions of selected compounds (IC_{50} experiments) in the presence of 5 mM $MgCl_2$ and 25 mM Tris-HCl (pH 8) for 10 minutes at the ambient temperature during the aliquoting and 10 minutes at 30 °C. The RNA synthesis was initiated by addition of a mixture containing 200 µM RNA primer, 2 µM RNA template, 0.1 µM [α-^{32}P]-GTP, and 0.1 µM NTP. Final concentration of DMSO in the reaction mixtures due to the addition of the compounds was 10%. Reactions (15 µL) were incubated for 45 minutes at 30 °C and then stopped by the addition of 15 µL of formamide/EDTA (25 mM) mixture and incubated at 95 °C for 10 min. 3 µL reaction samples were subjected to denaturing 8 M urea 20% polyacrylamide gel electrophoresis to resolve products of RNA synthesis followed by

signal quantification (ImageQuant 5.2, GE Healthcare Bio-Sciences, Uppsala, Sweden) through phosphorimaging (Amerhsham Typhoon 5, Cytivia, Marlborough, MA, USA). The signal corresponding to the full template-length product of RNA synthesis in the presence of a compound was normalized (%) to the signal obtained in the absence of the compound. The normalized values of RNA synthesis were plotted versus compound concentrations and fitted to a log(inhibitor)-versus-normalized-response-(variable slope) equation using GraphPad Prism 7.0 (GraphPad Software, Inc., San Diego, CA, USA) to determine the IC_{50} values for the inhibition of RNA synthesis by a given compound. Suramine and remdesivir were used as positive controls (see table 1 and figure S16).

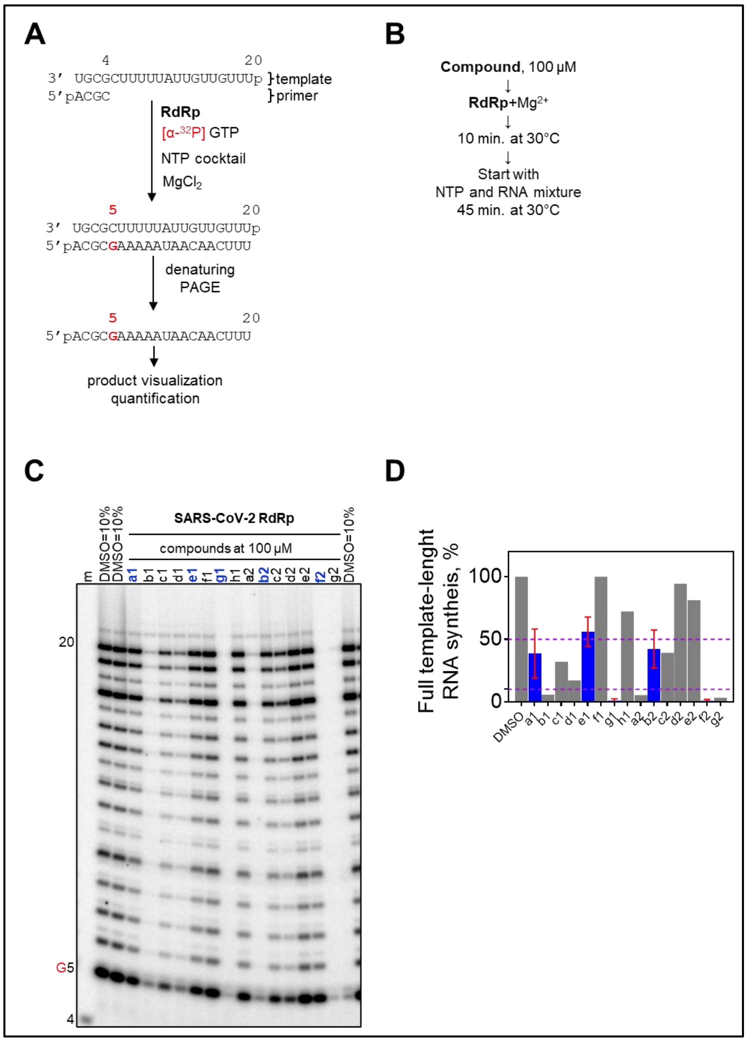

Figure S14 SARS-CoV-2 RdRp complex inhibition assay. (**A**) Schematic representation of the reaction and data analysis. RNA synthesis by purified SARS-CoV-2 RdRp complex is monitored with a short, 4-nt RNA primer and a 20-nt RNA template in the presence of NTPs and $MgCl_2$. Incorporation of [α-^{32}P]GTP at position 5 provides the label. (**B**) Order of addition. Compounds with potential inhibitory activity are allowed to interact with the SARS-CoV-2 RdRp complex prior to the addition of the RNA and NTP substrates. (**C**) RNA synthesis catalyzed by SARS-CoV-2 RdRp complex in the absence and presence of the selected compounds. Bands corresponding to the first incorporated nucleotide (G5) and to the full template-length products (20) are indicated on the left. The 5'-^{32}P-labeled 4-nt primer (4) is used as a size marker (m). A set of sandacrabins is highlighted in blue. (**D**) Graphical representation of the data shown in panel C. Horizontal purple dashed lines illustrate 50% and 90% percent inhibition, respectively. A set of sandacrabins is highlighted in blue. Error bars illustrate standard deviation of the data points within a set of four independent experiments.

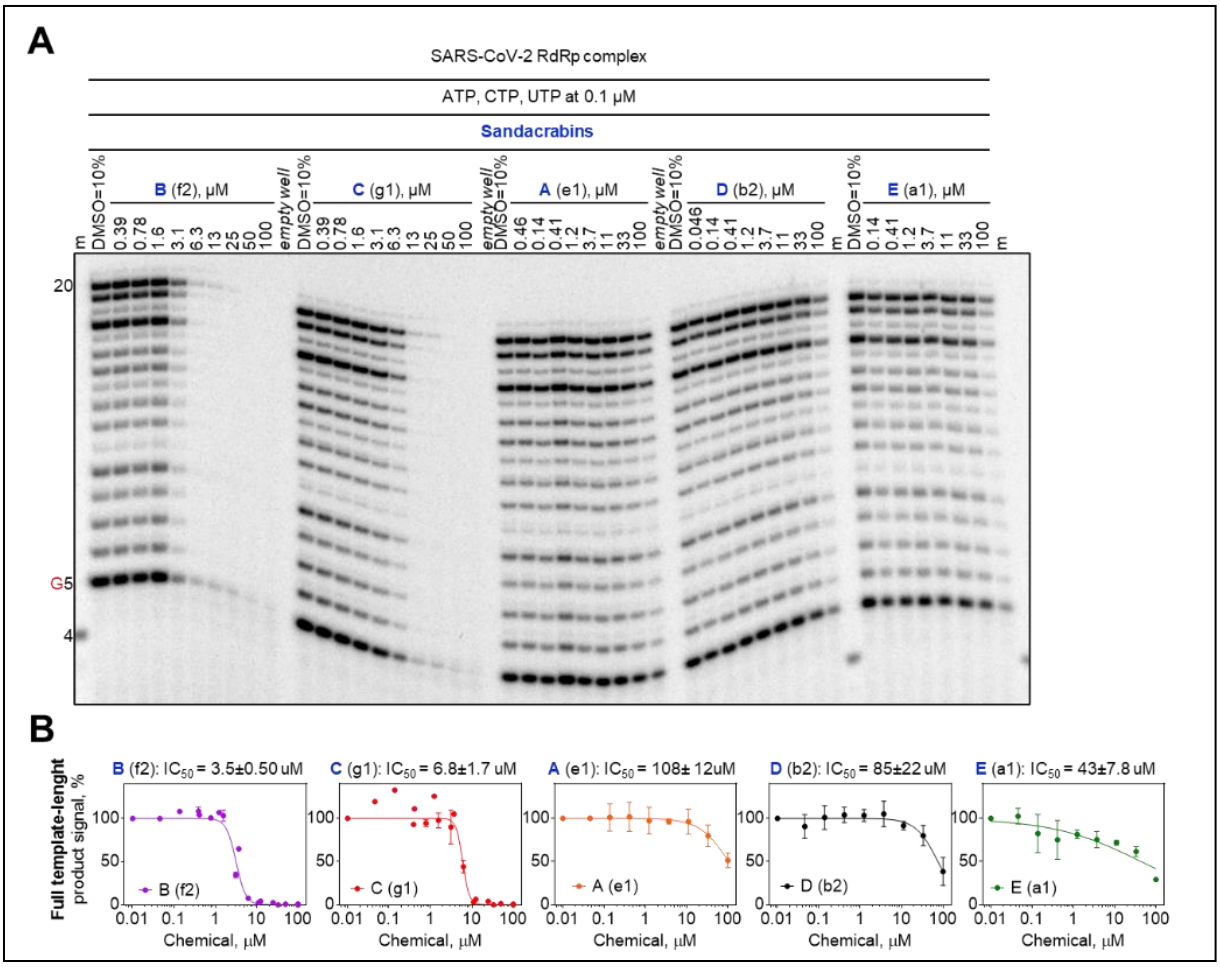

Figure S15 Sandacrabin-dependent inhibition of RNA synthesis catalyzed by SARS-CoV-2 RdRp complex. (A) RNA synthesis catalyzed by SARS-CoV-2 RdRp complex in the presence of the serial dilutions of the selected set of sandacrabin compounds. Bands corresponding to the first incorporated nucleotide (G5) and to the full template-length products (20) are indicated on the left. The 5'-^{32}P-labeled 4-nt primer (4) is used as a size marker (m). (B) Graphical representation of the data and data analysis used to determine the IC_{50} values of the selected inhibitors shown in panel B. The experiments were repeated three times.

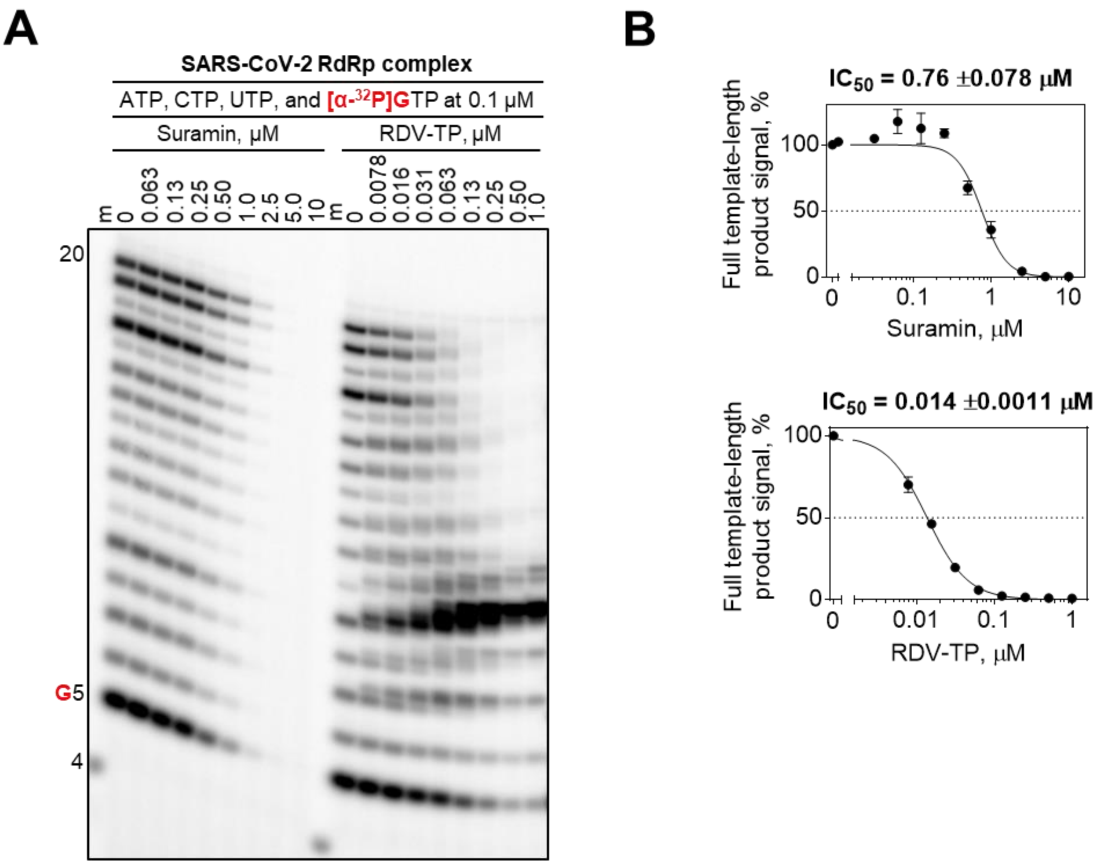

Figure S16 Suramin- and remdesivir-triphosphate (RDV-TP)-dependent inhibition of RNA synthesis catalyzed by SARS-CoV-2 RdRp complex. (A) RNA synthesis catalyzed by SARS-CoV-2 RdRp complex in the presence of the serial dilutions of indicated compounds. Bands corresponding to the first incorporated nucleotide (G5) and to the full template-length products (20) are indicated on the left. The 5'-^{32}P-labeled 4-nt primer (4) is used as a size marker (m). (B) Graphical representation of the data and data analysis used to determine the IC_{50} values of the indicated compounds shown in panel A. The experiments were repeated three times.

S8 NMR spectra

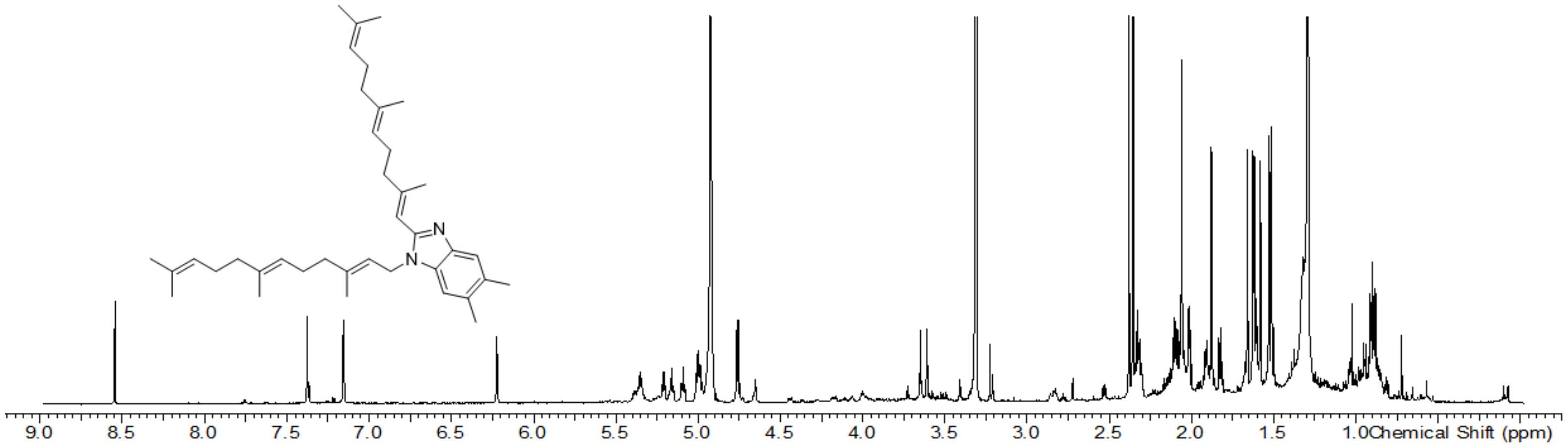

Figure S17 ^{1}H spectrum of sandacrabin A in methanol-d_4 500 MHz.

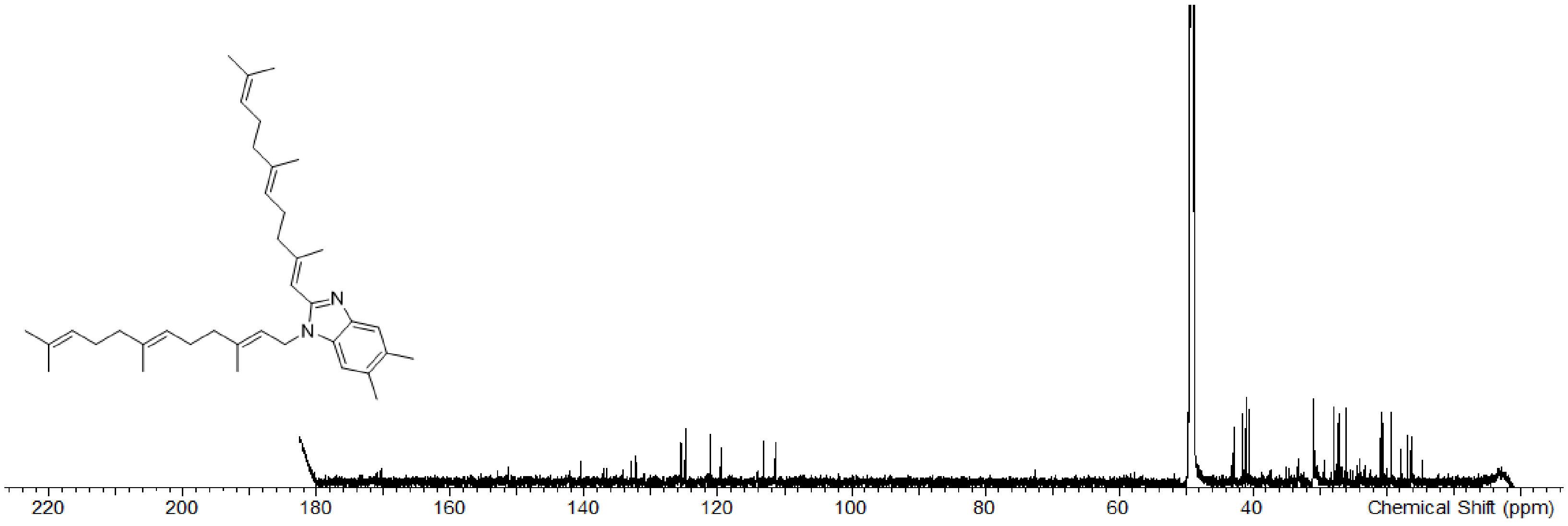

Figure S18 ^{13}C spectrum of sandacrabin A in methanol-d_4 125 MHz.

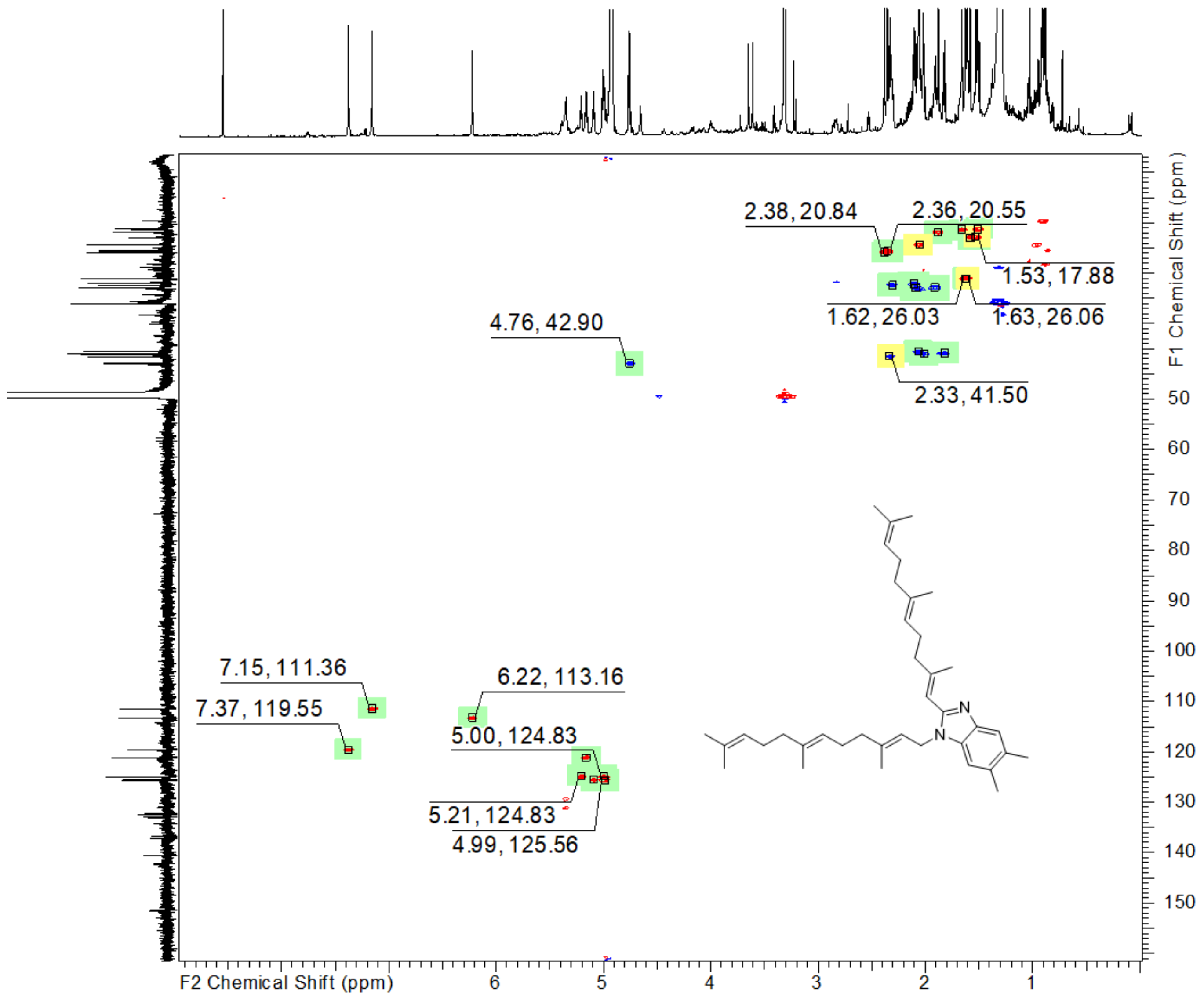

Figure S19 HSQC spectrum of sandacrabin A in methanol-d_4 500/125 MHz.

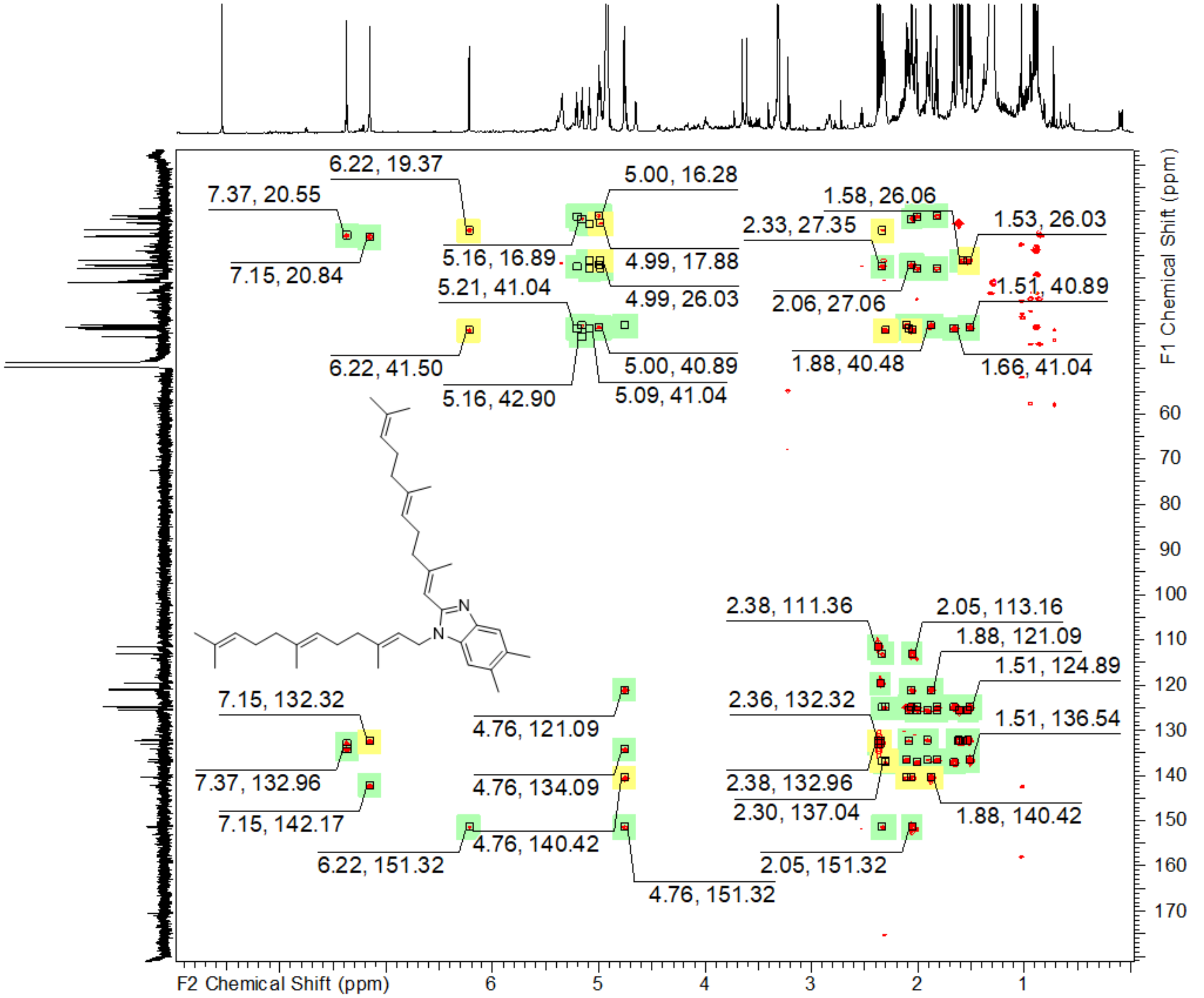

Figure S20 HMBC spectrum of sandacrabin A in methanol-d_4 500/125 MHz.

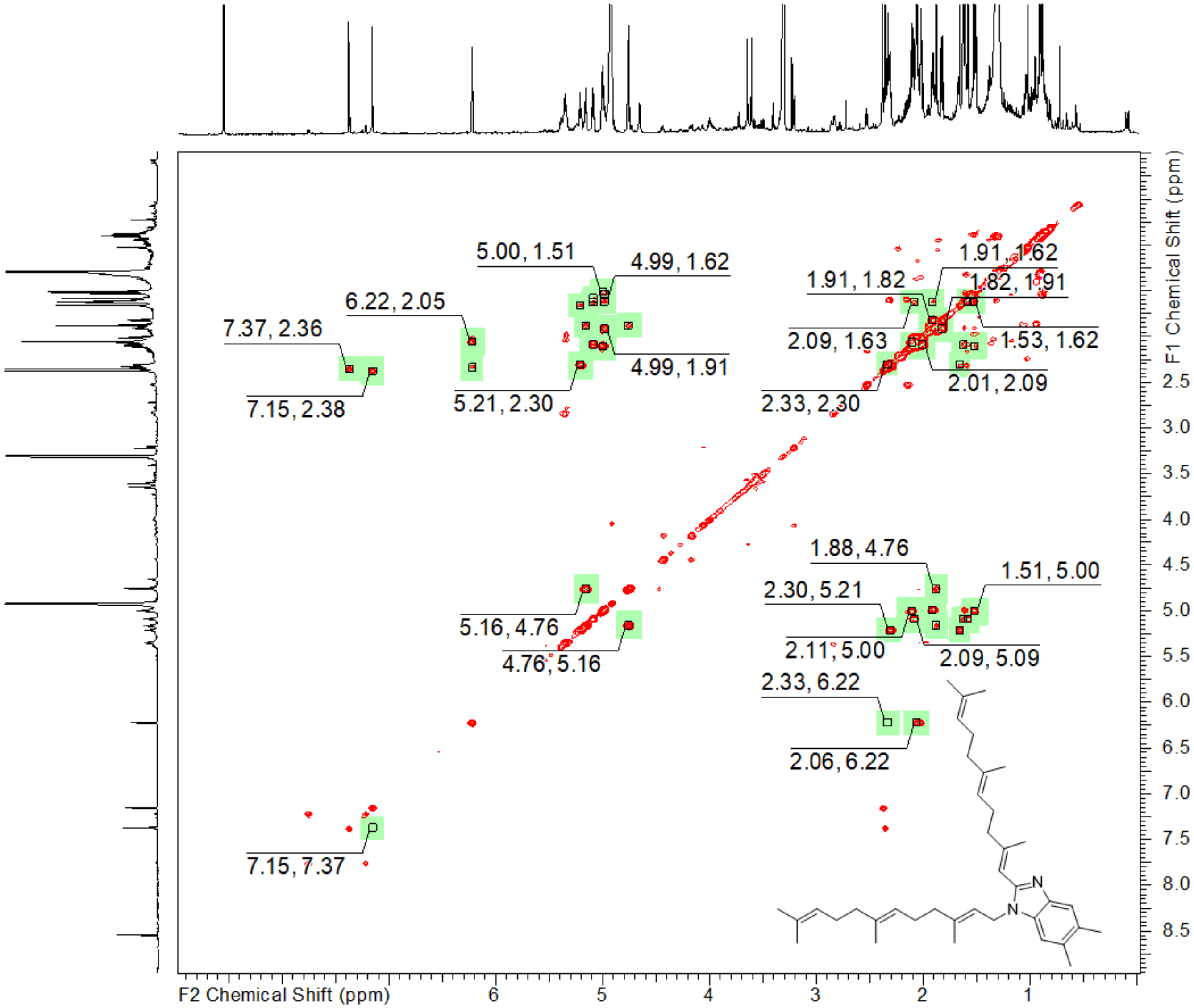

Figure S21 COSY spectrum of sandacrabin A in methanol-d_4 500 MHz.

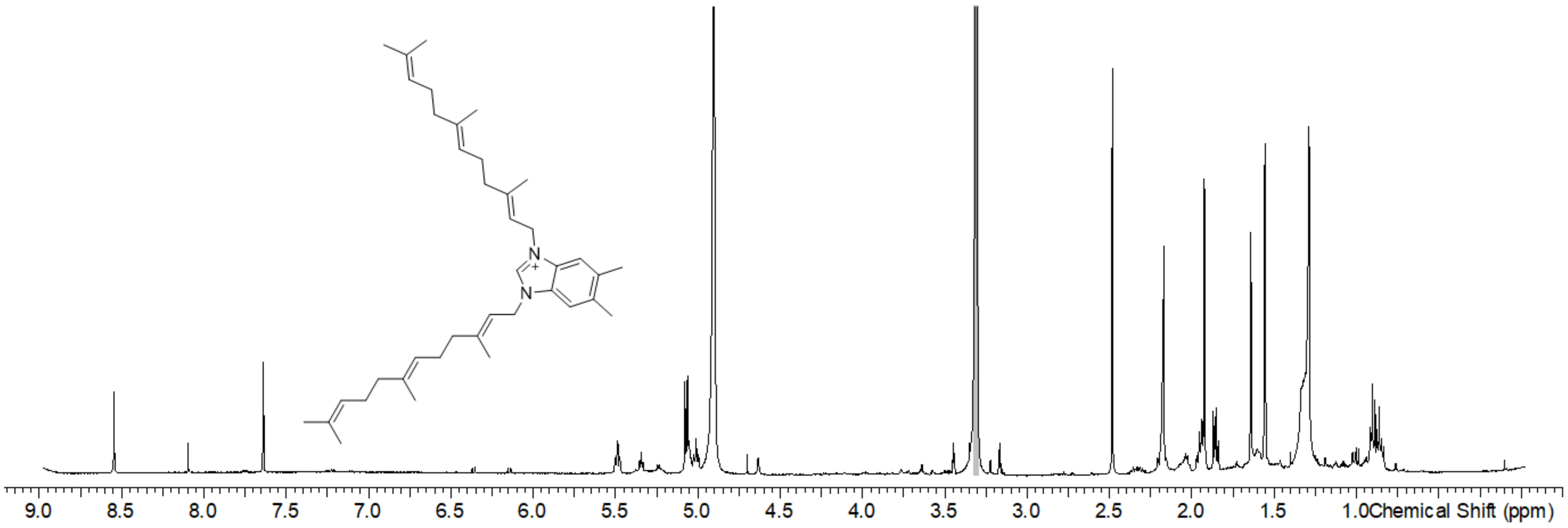

Figure S22 ^{1}H spectrum of sandacrabin B in methanol-d_4 500 MHz.

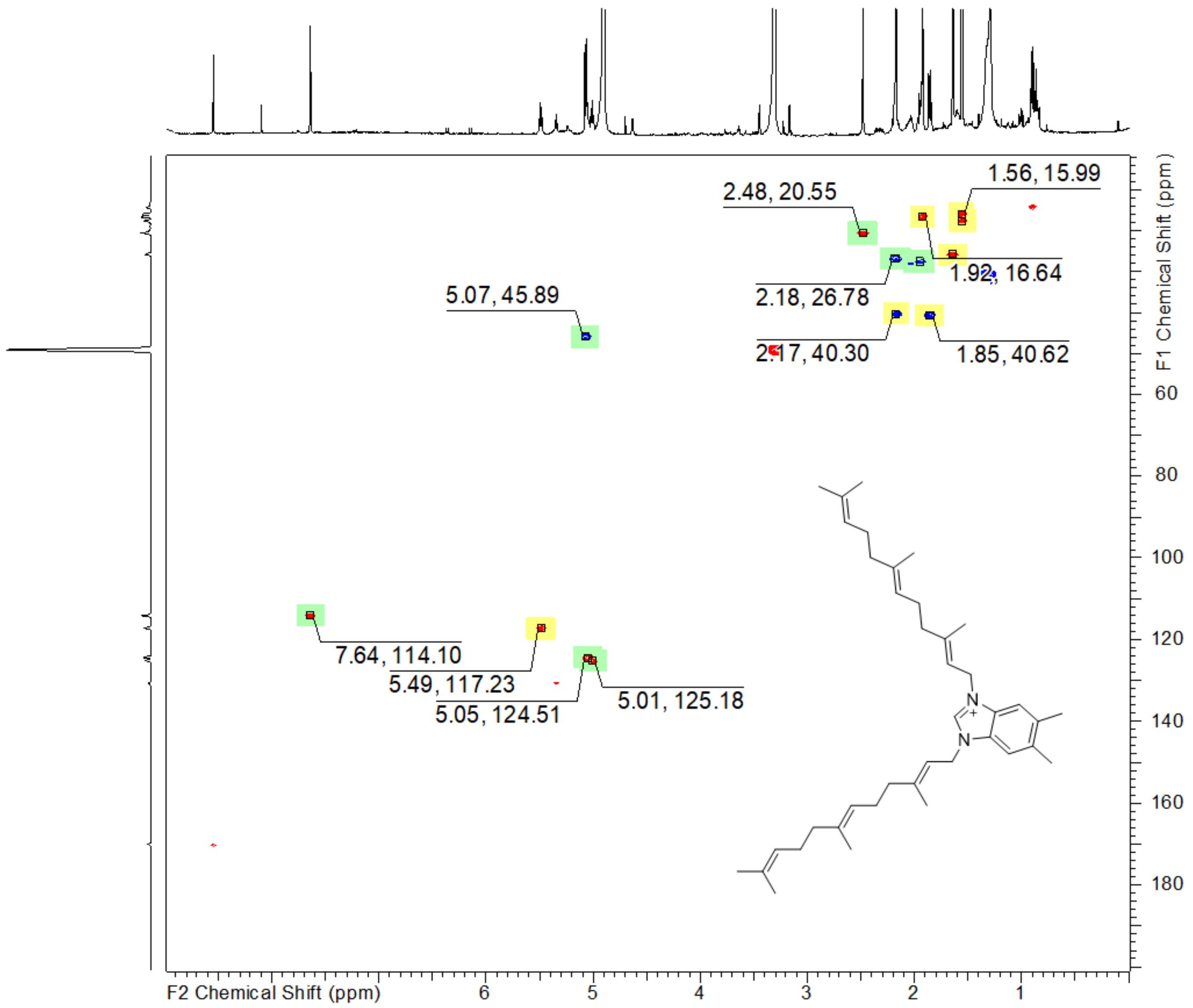

Figure S23 HSQC spectrum of sandacrabin B in methanol-d_4 500/125 MHz.

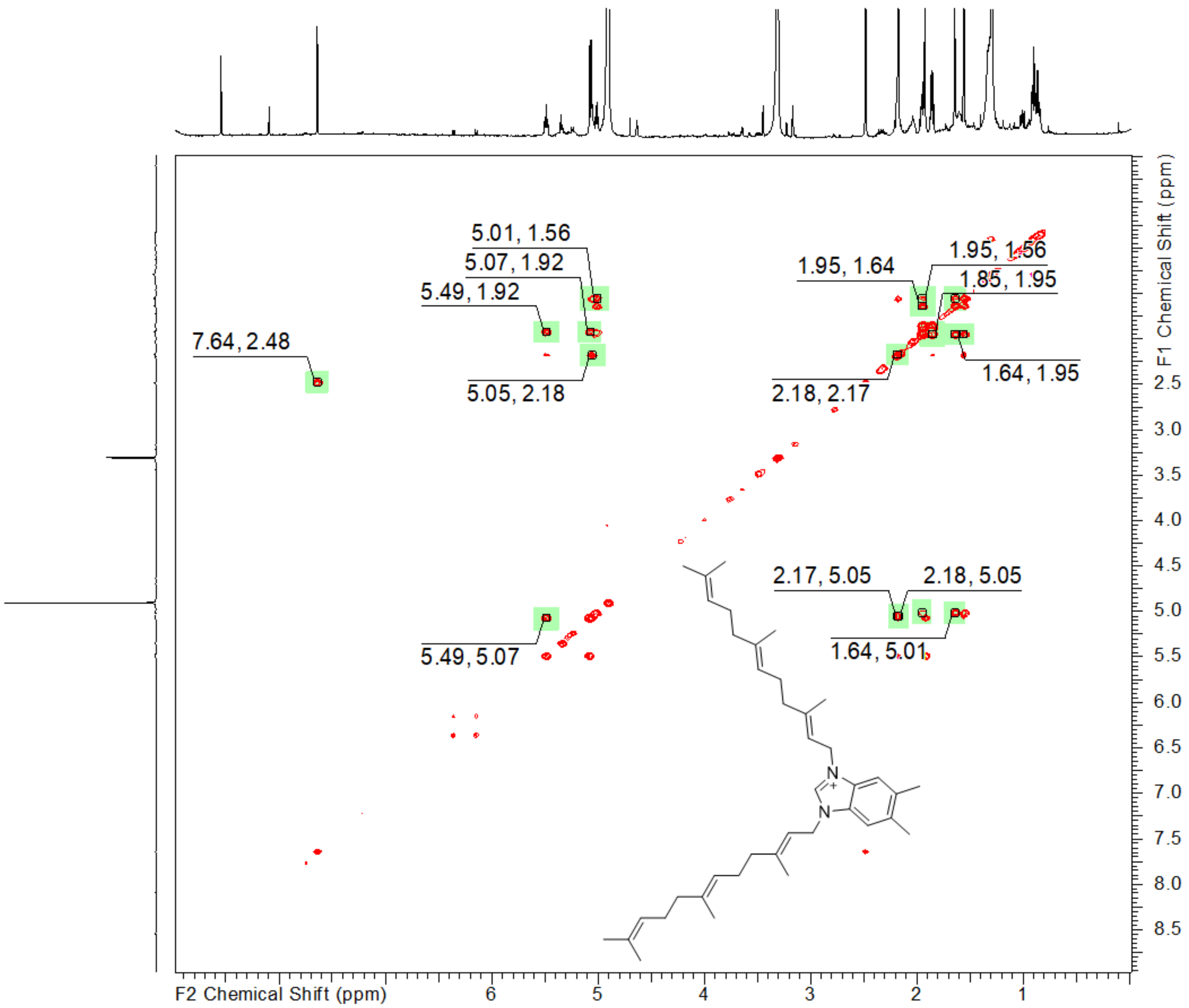

Figure S24 COSY spectrum of sandacrabin B in methanol-d_4 500 MHz.

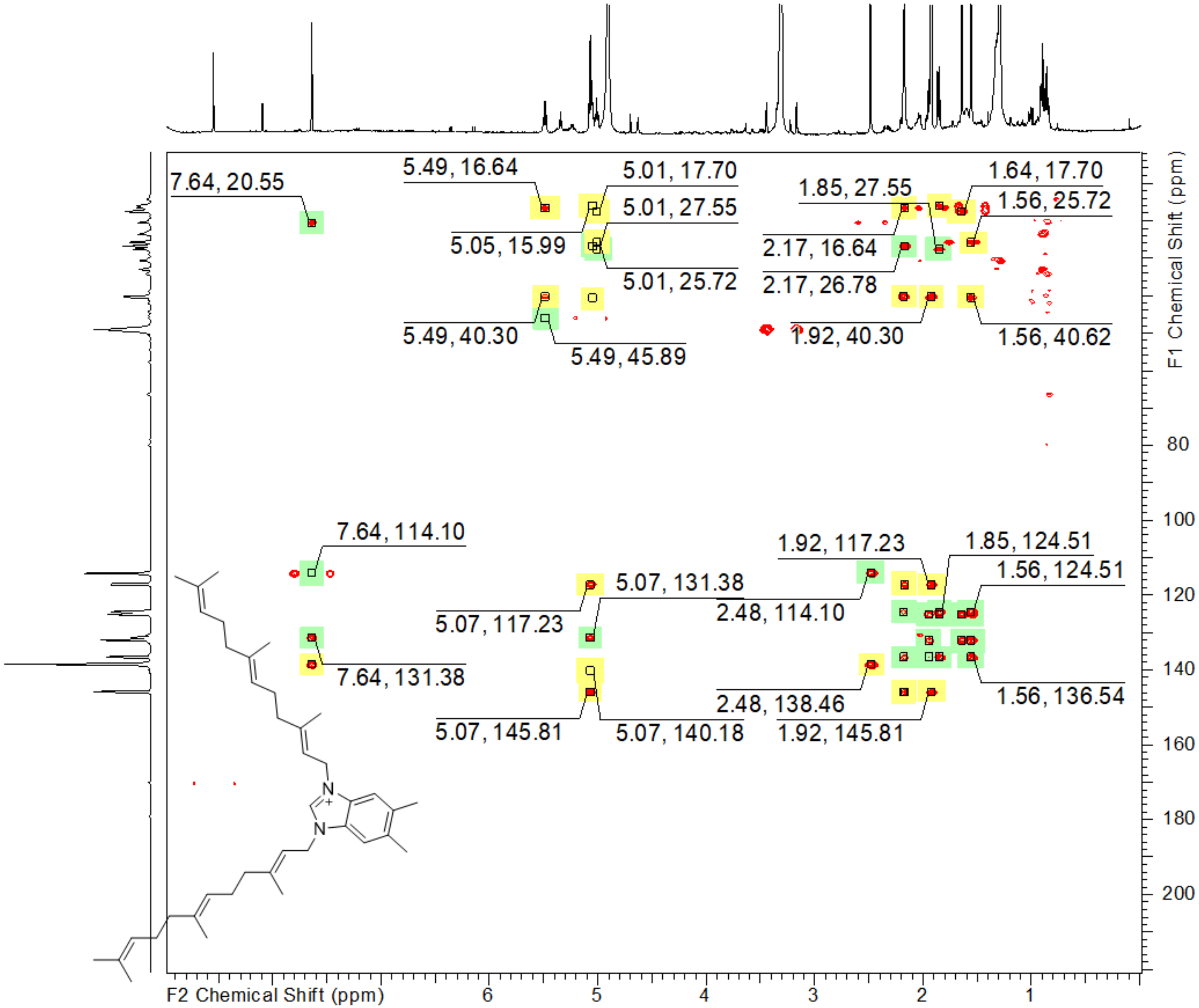

Figure S25 HMBC spectrum of sandacrabin B in methanol-d_4 500/125 MHz.

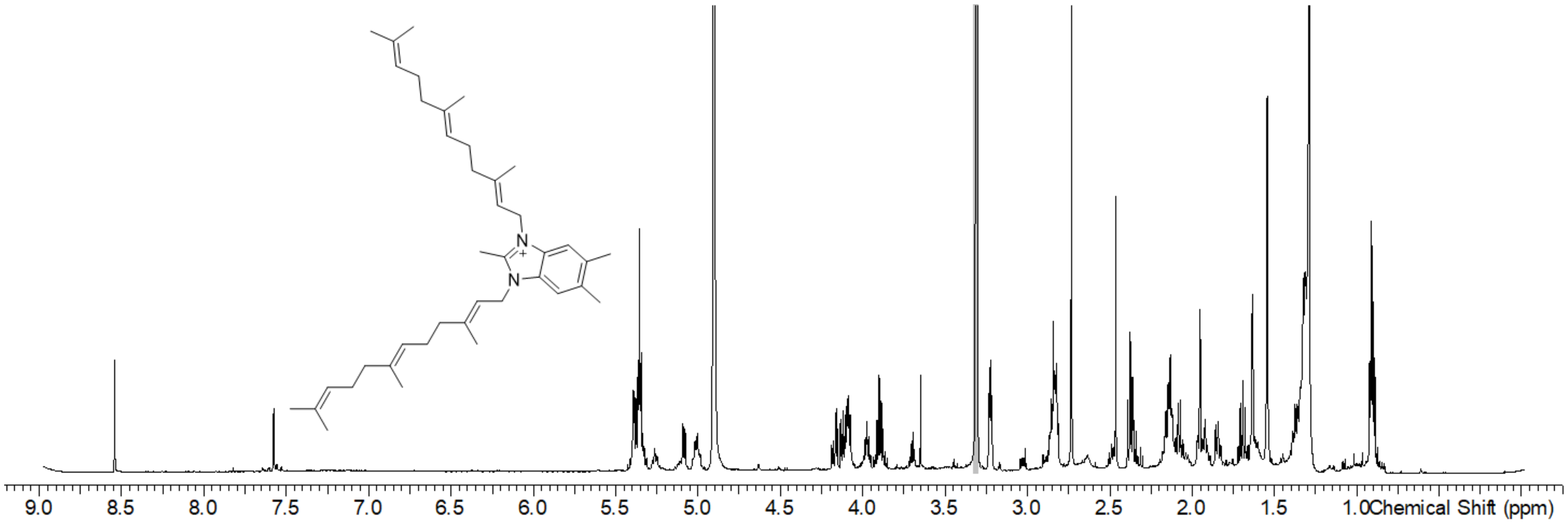

Figure S26 ^{1}H spectrum of sandacrabin C in methanol-d_4 500 MHz.

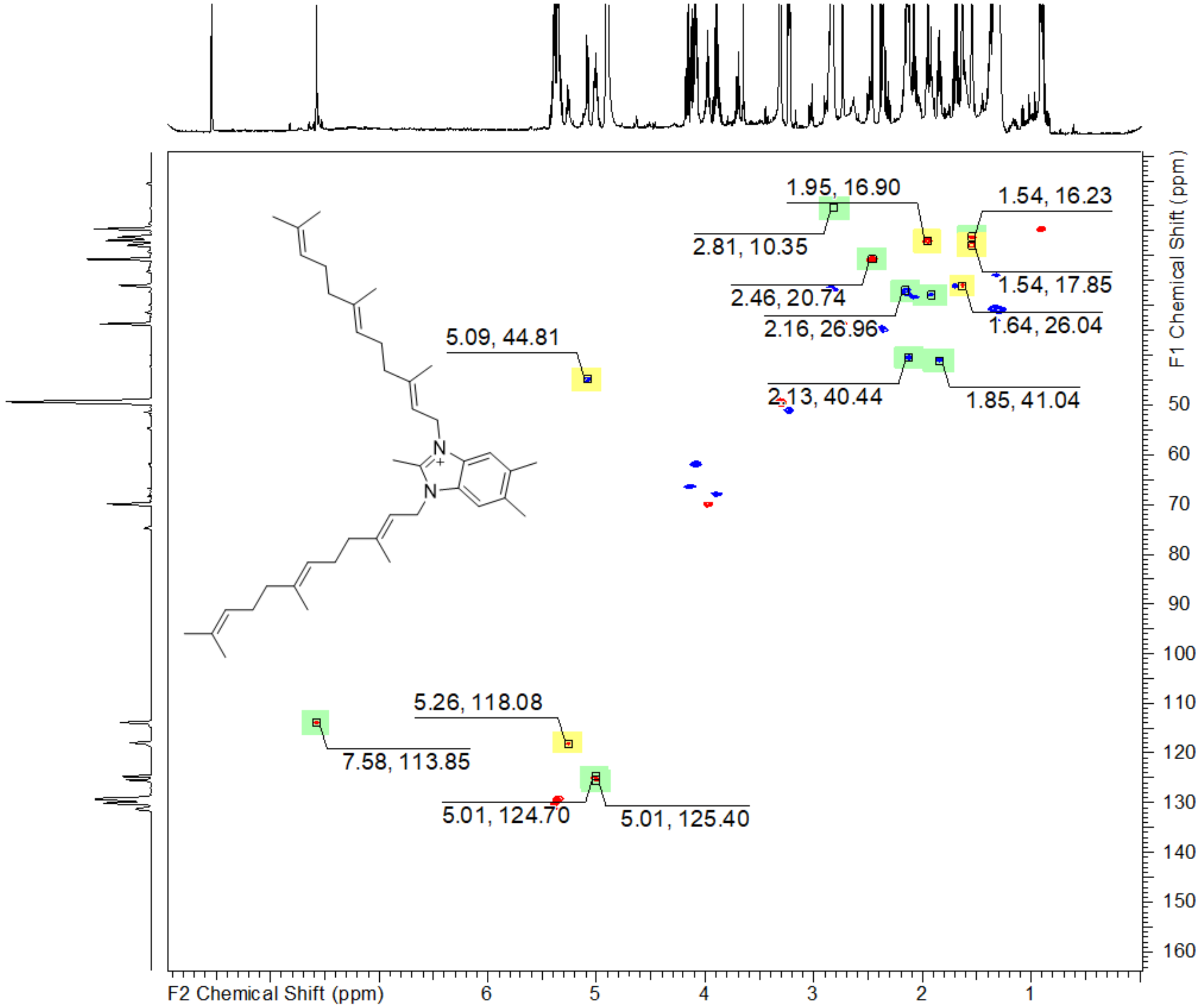

Figure S27 HSQC spectrum of sandacrabin C in methanol-d_4 500/125 MHz.

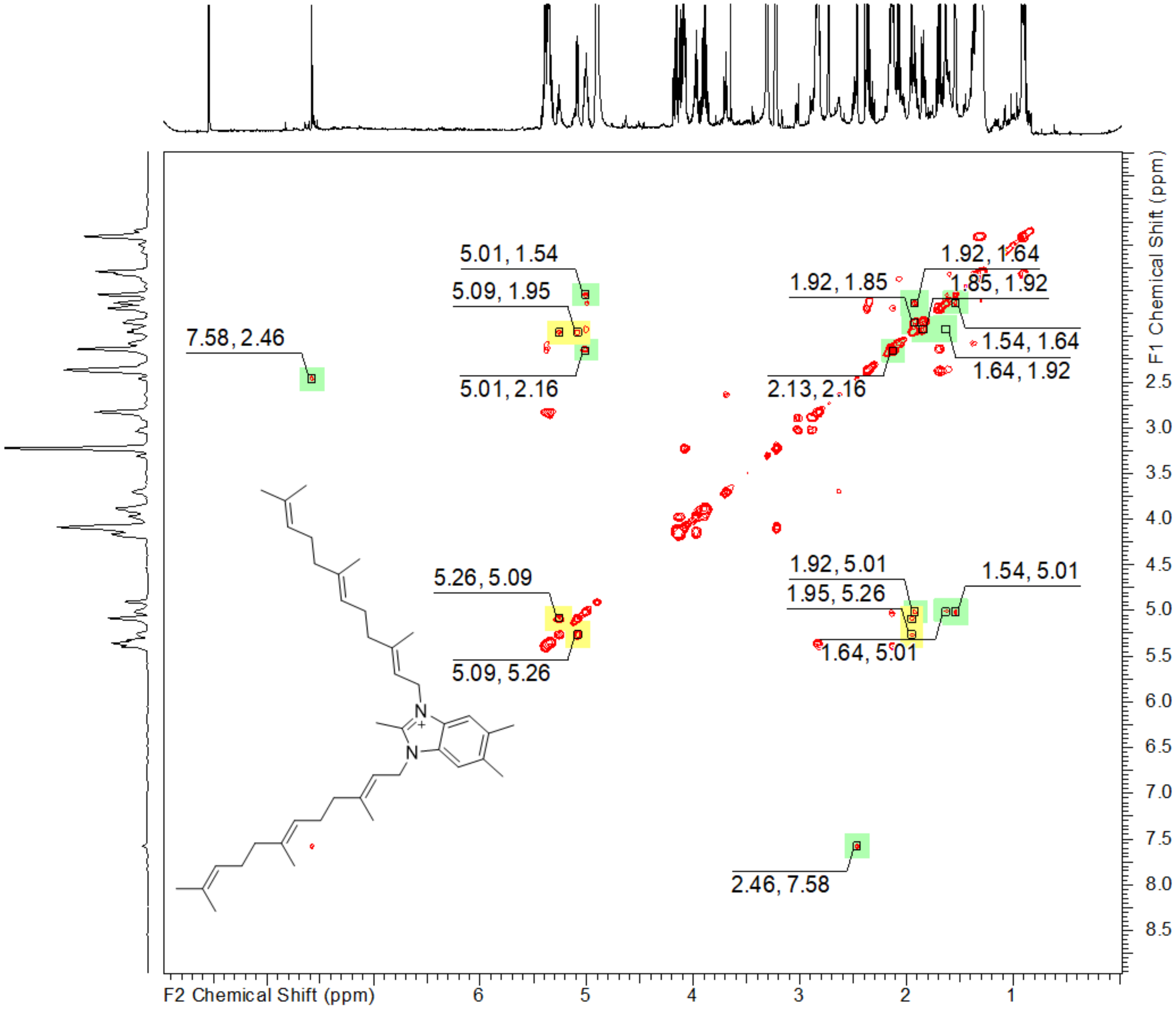

Figure S28 COSY spectrum of sandacrabin C in methanol-d_4 500 MHz.

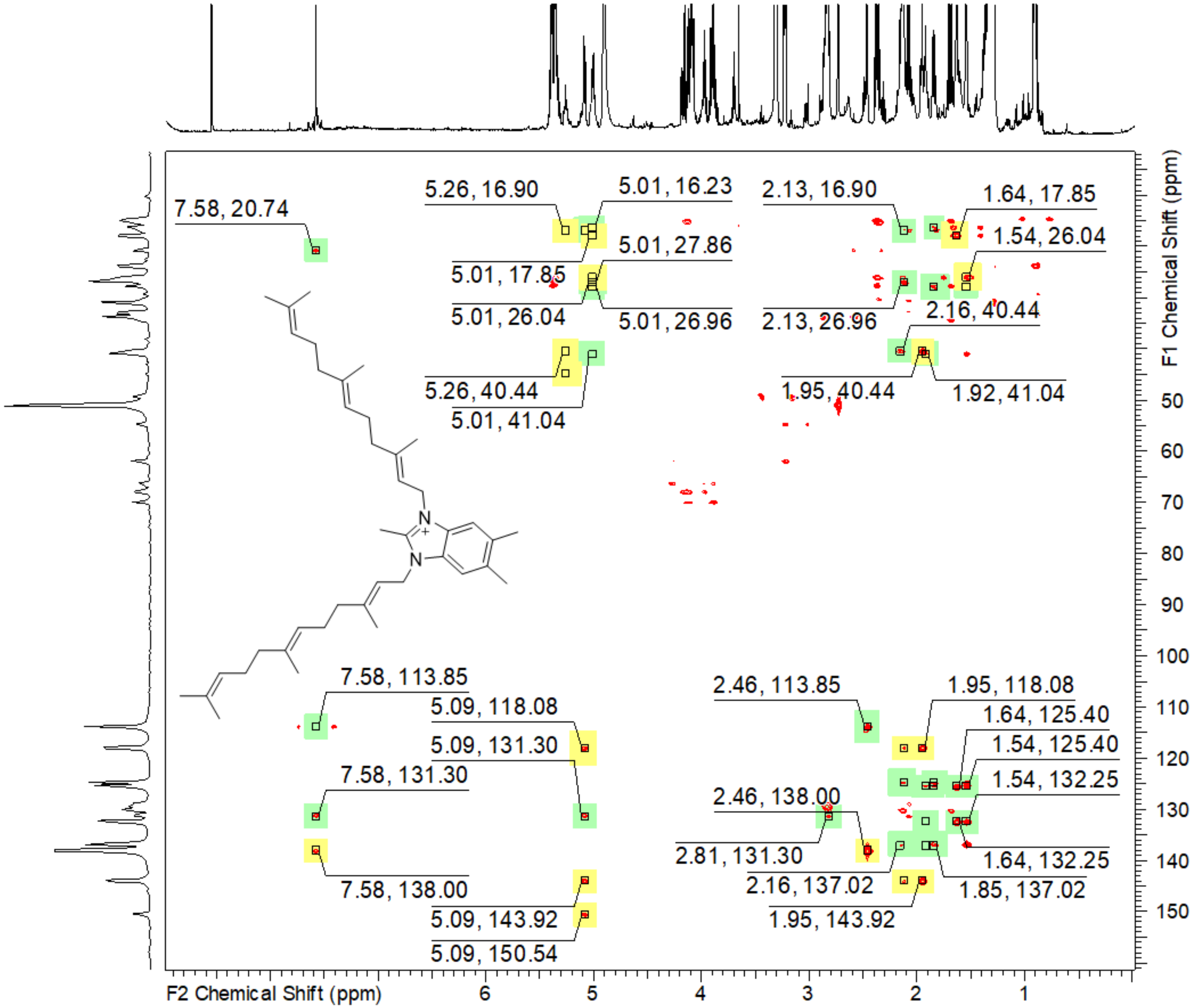

Figure S29 HMBC spectrum of sandacrabin C in methanol-d_4 500/125 MHz.

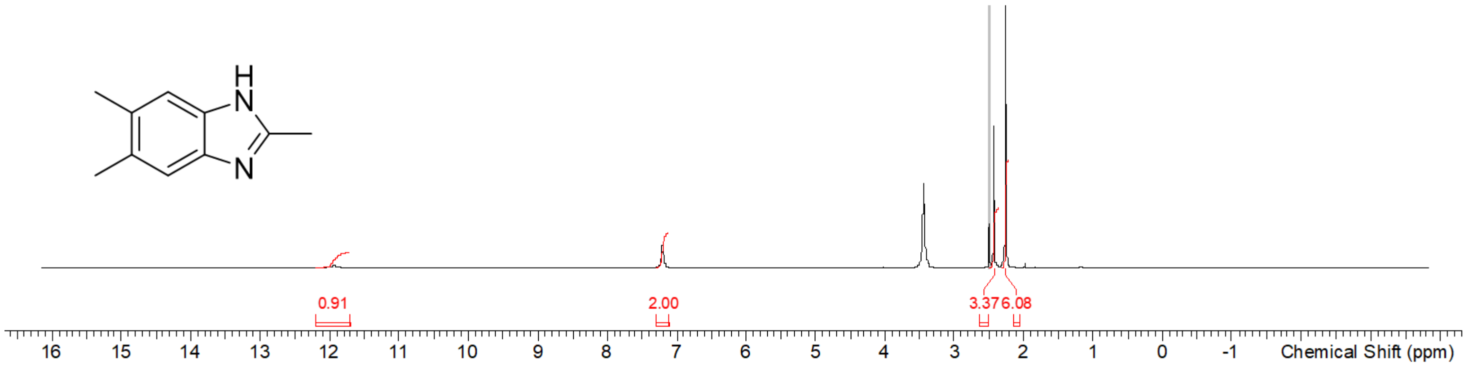

Figure S30 ^{1}H spectrum of **1** in DMSO-d_6 at 500 MHz.

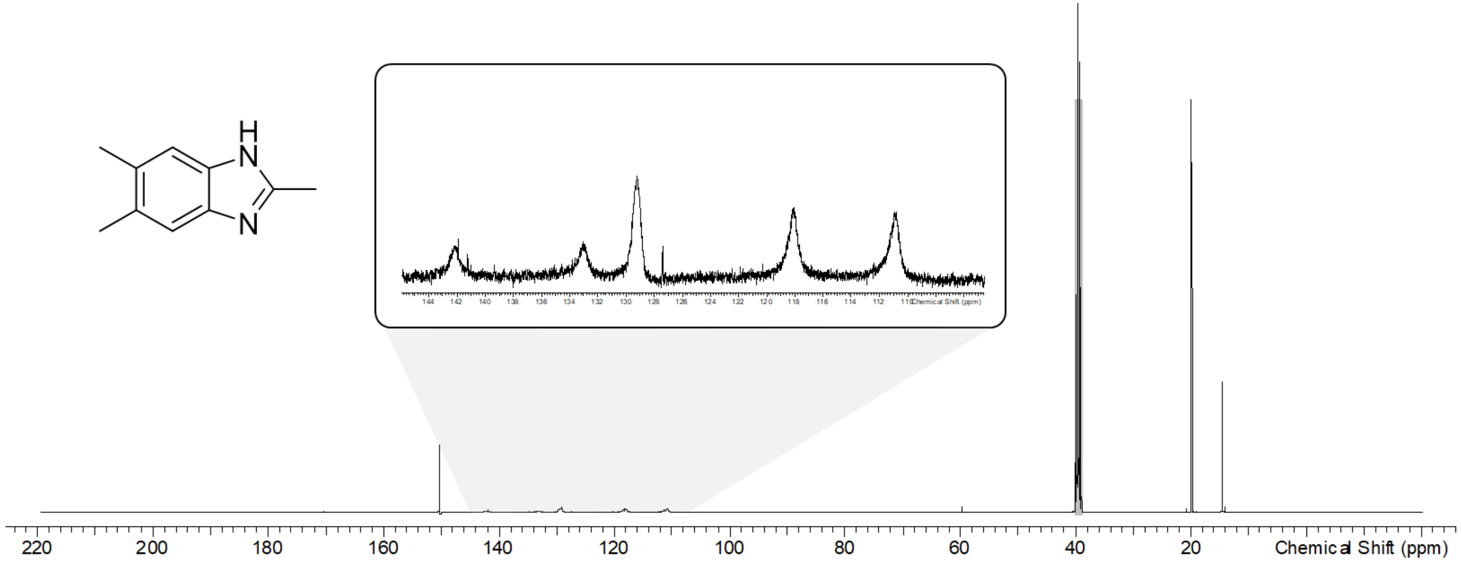

Figure S31 ^{13}C spectrum of **1** in DMSO-d_6 at 125 MHz.

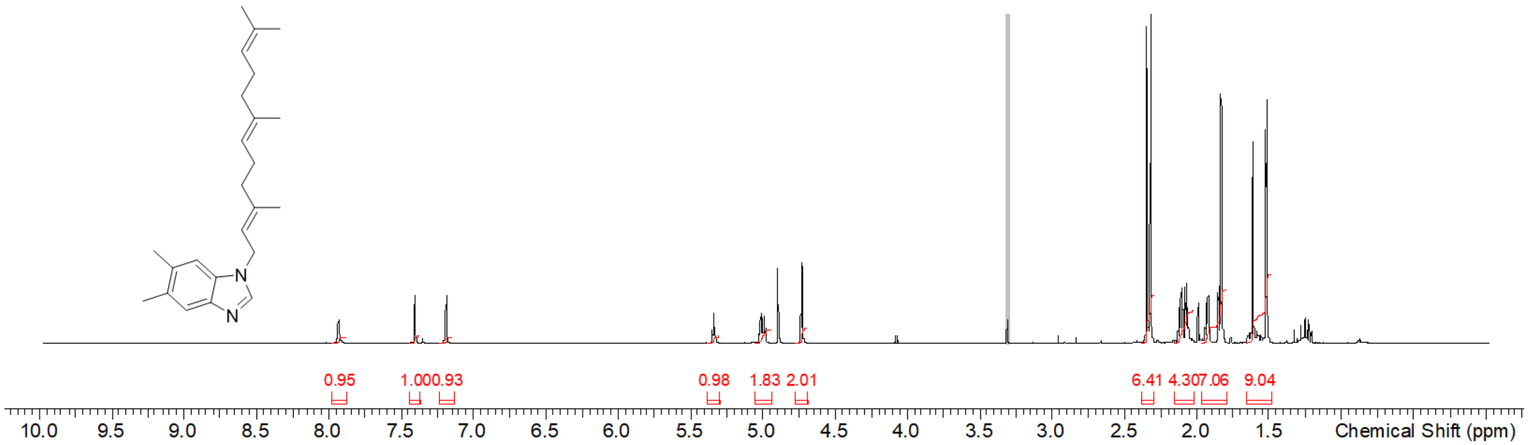

Figure S32 ^{1}H spectrum of **2** in MeOD-d_4 at 700 MHz.

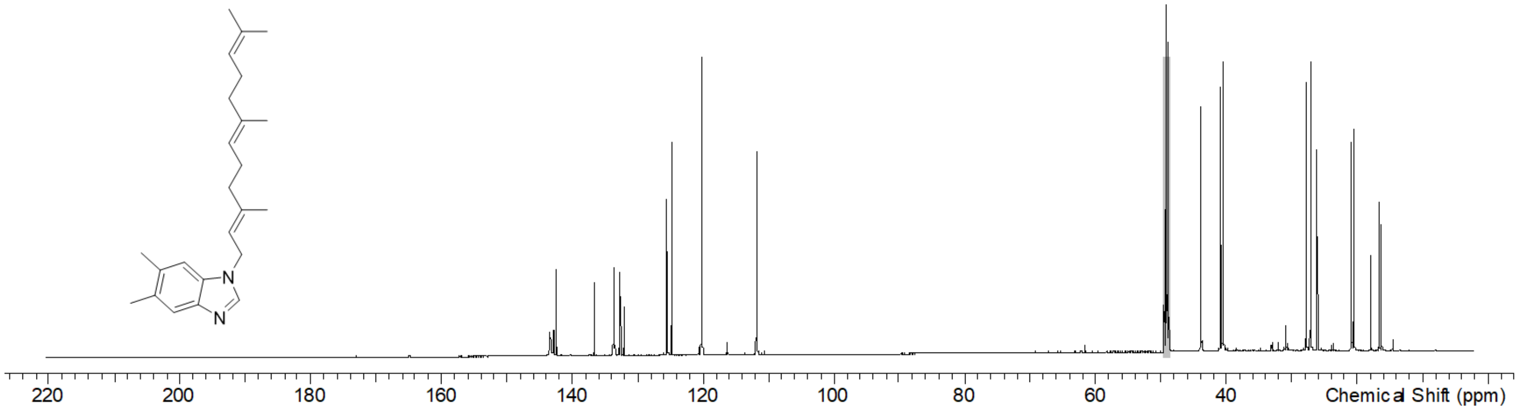

Figure S33 ^{13}C spectrum of **2** in MeOD-d_4 at 175 MHz.

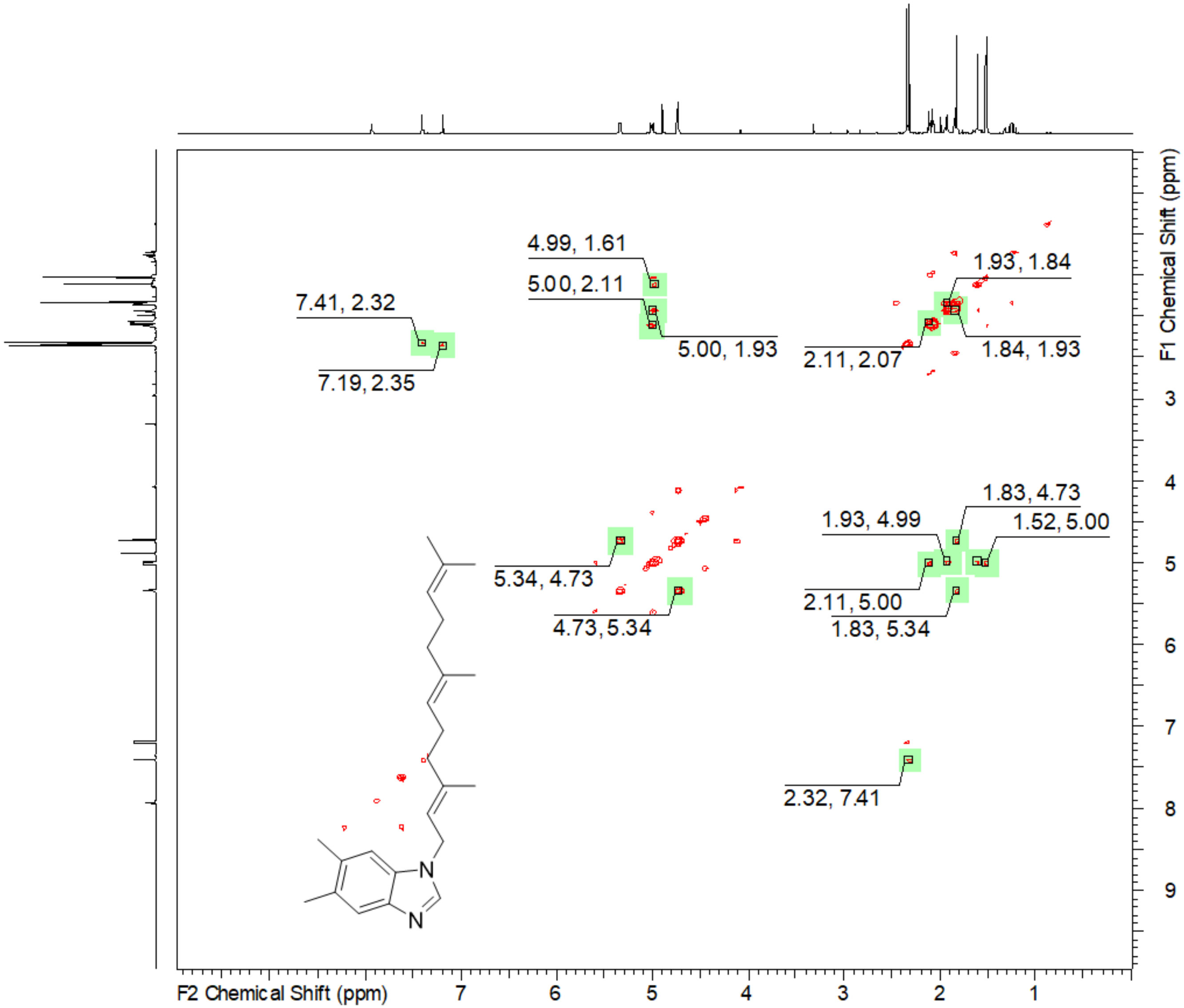

Figure S34 COSY spectrum of **2** in MeOD-d_4 at 700 MHz.

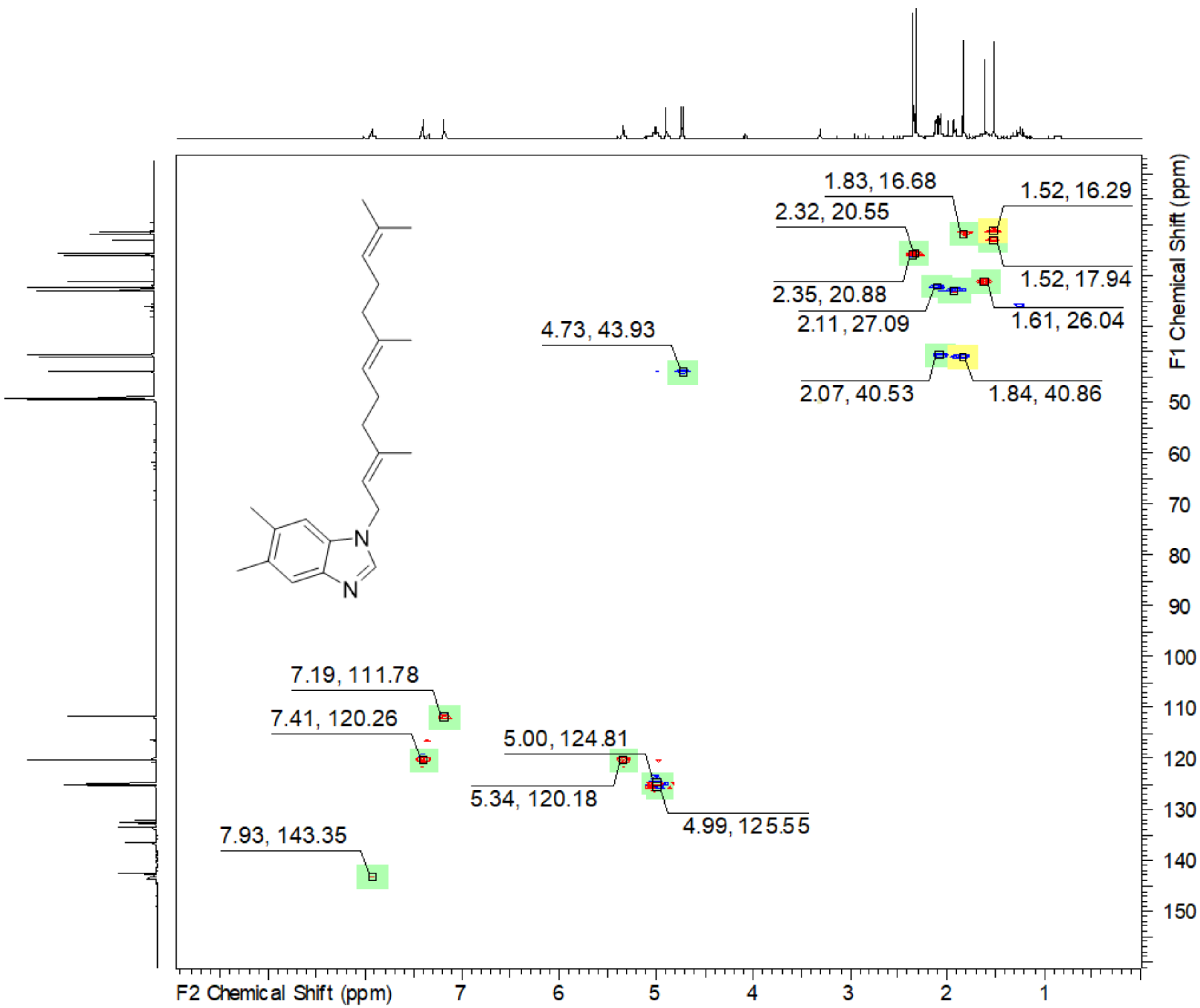

Figure S35 HSQC spectrum of **2** in MeOD-d_4 at 700/175 MHz.

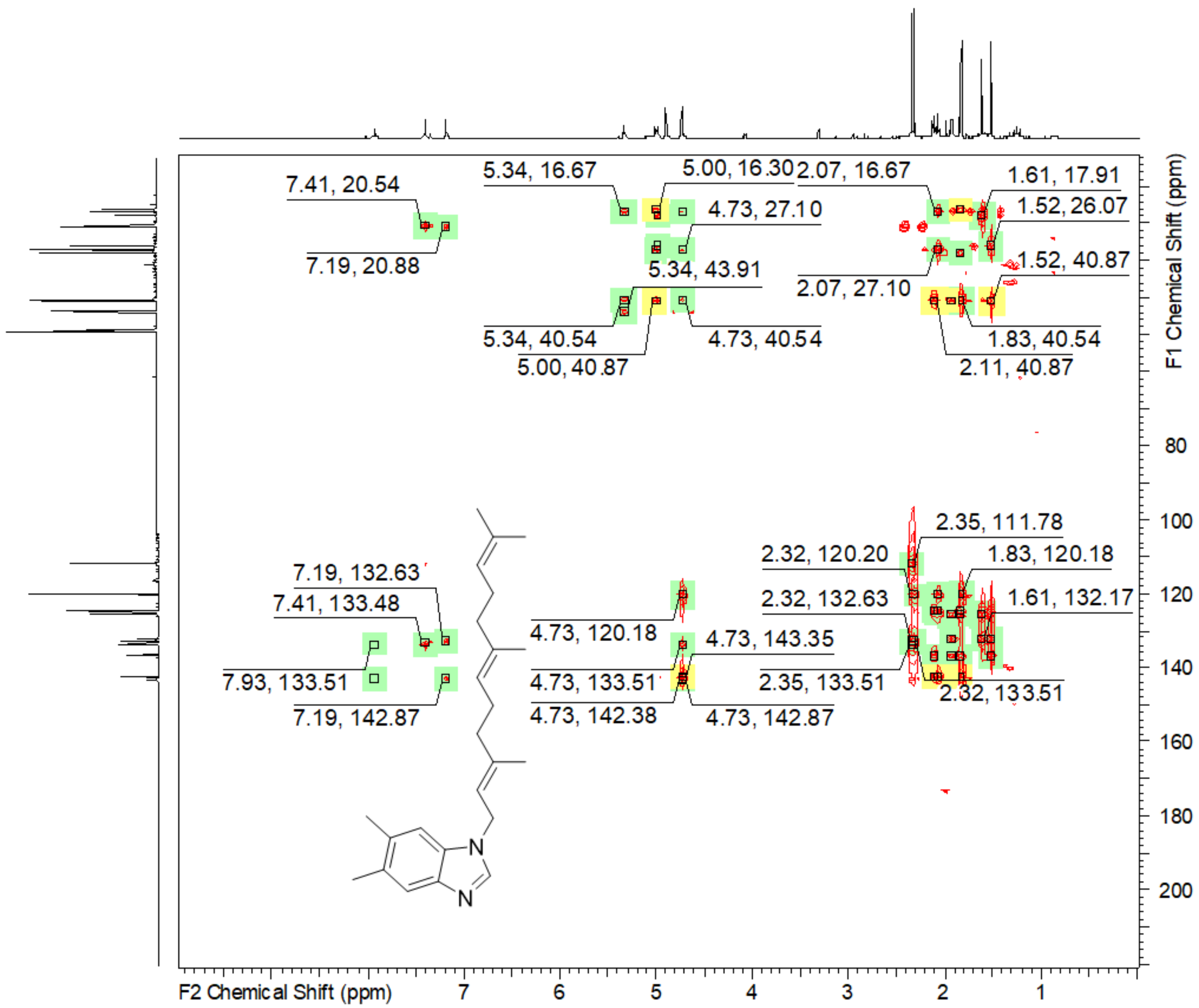

Figure S36 HMBC spectrum of **2** in MeOD-d_4 at 700/175 MHz.

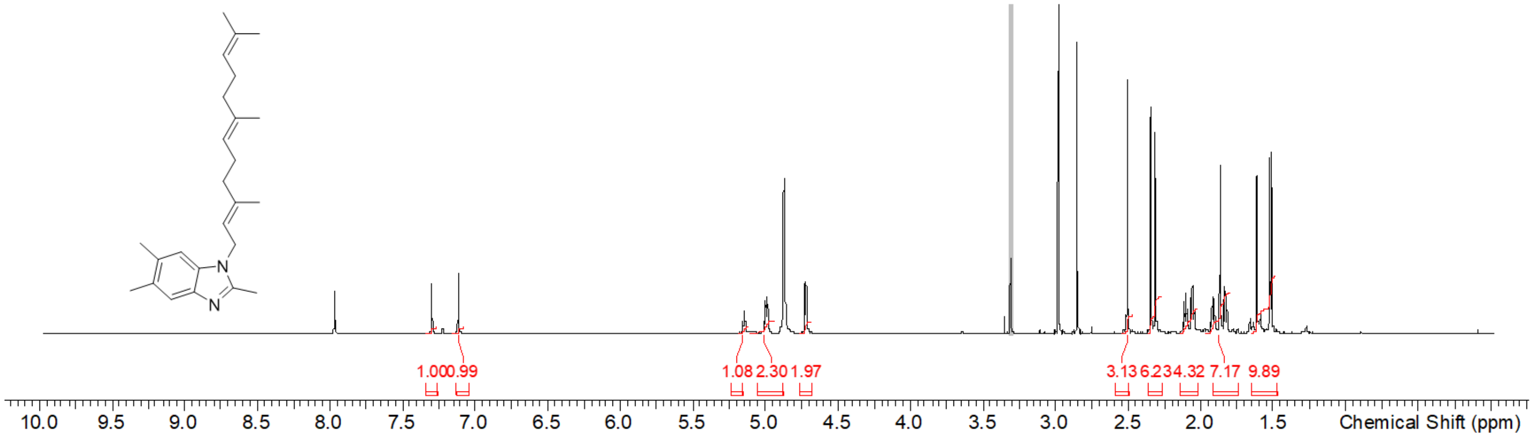

Figure S37 ^{1}H spectrum of **3** in MeOD-d_4 at 700 MHz.

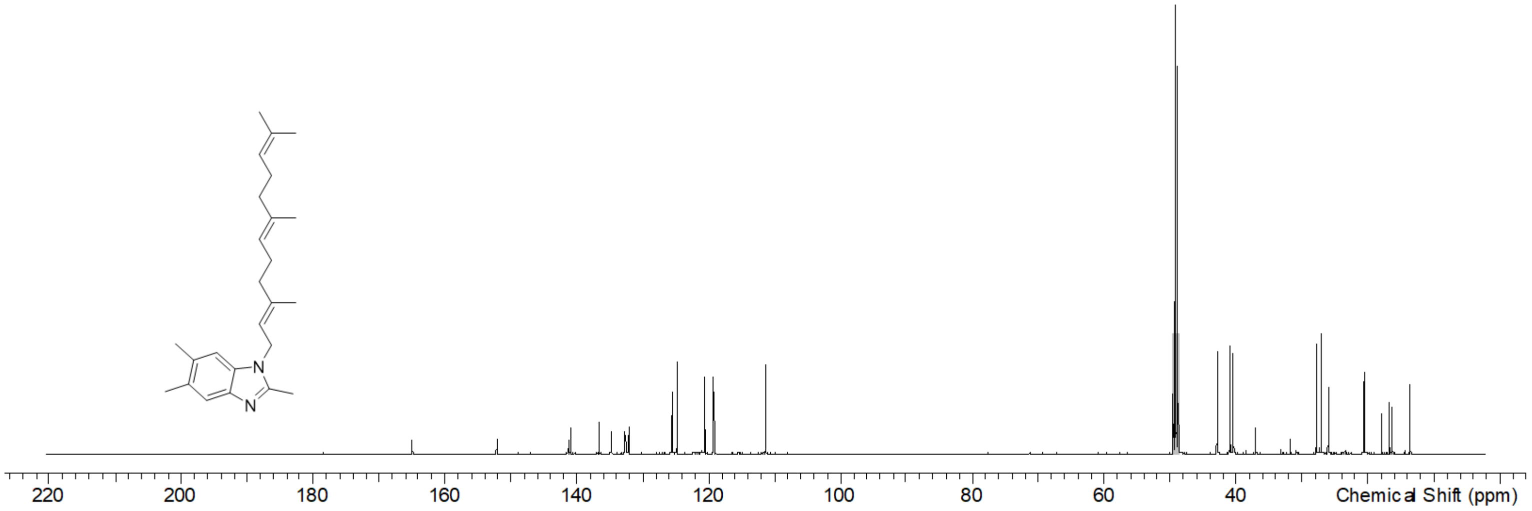

Figure S38 ^{13}C spectrum of **3** in MeOD-d_4 at 175 MHz.

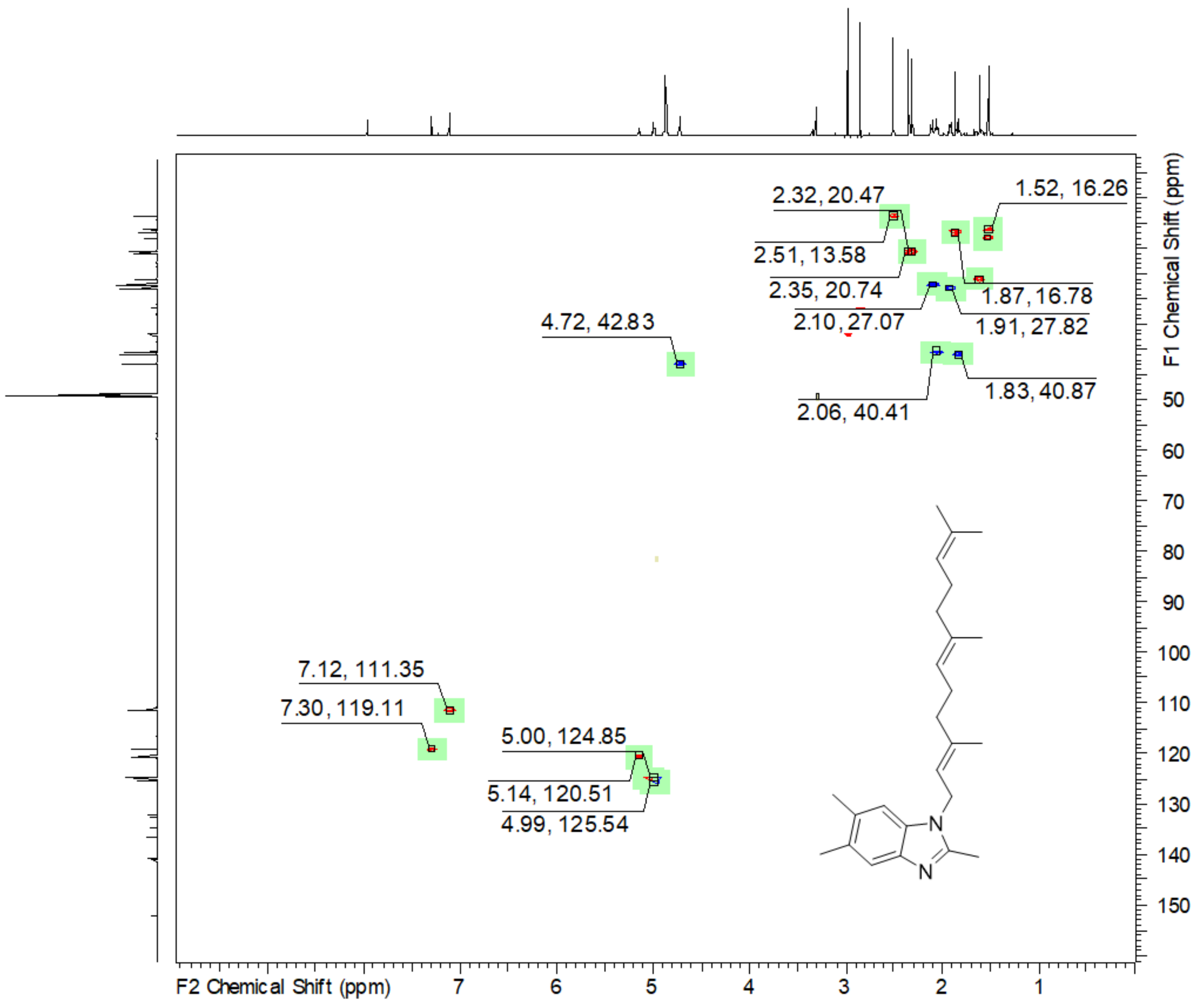

Figure S39 HSQC spectrum of **3** in MeOD-d_4 at 700/175 MHz.

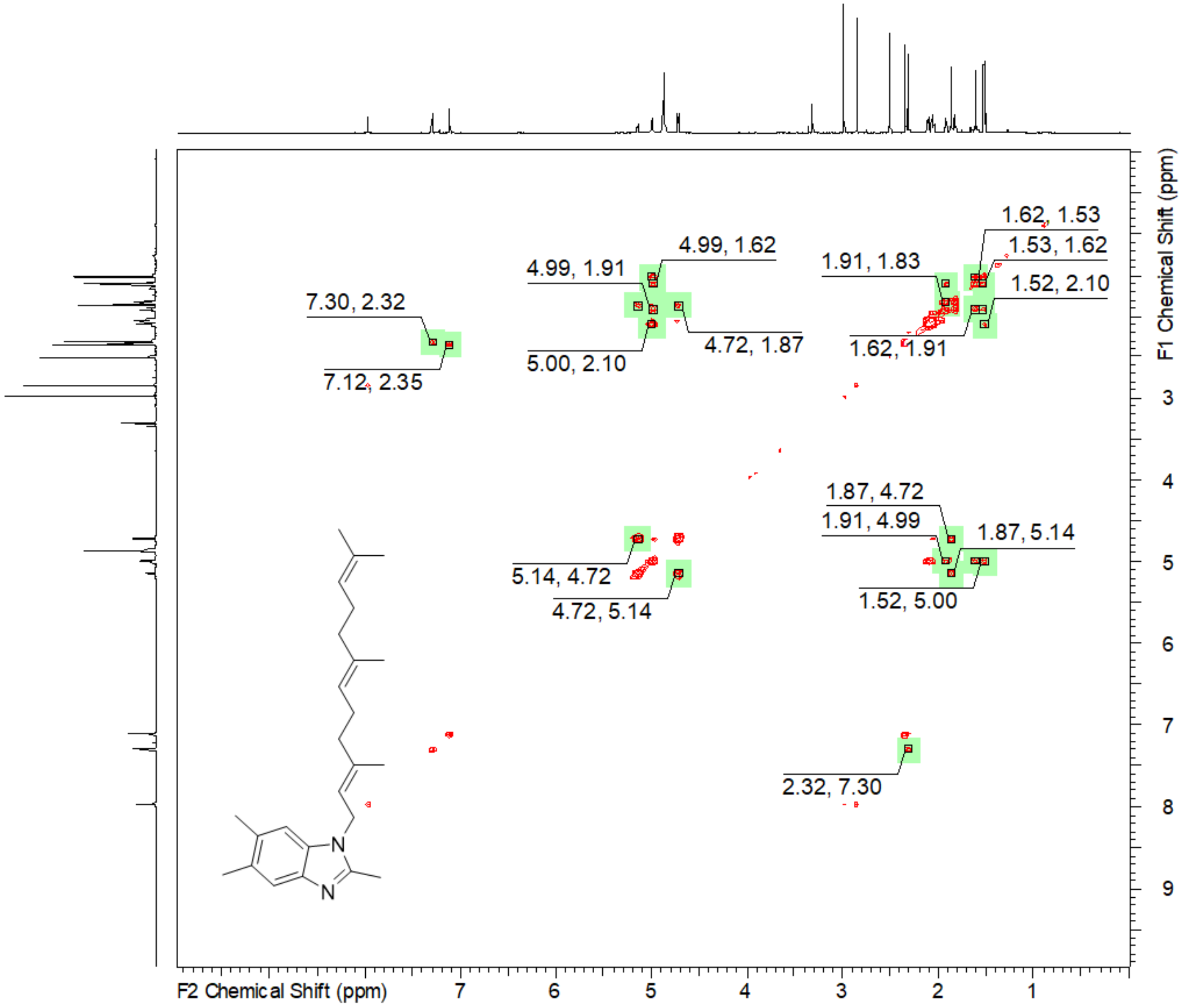

Figure S40 COSY spectrum of **3** in MeOD-d_4 at 700 MHz.

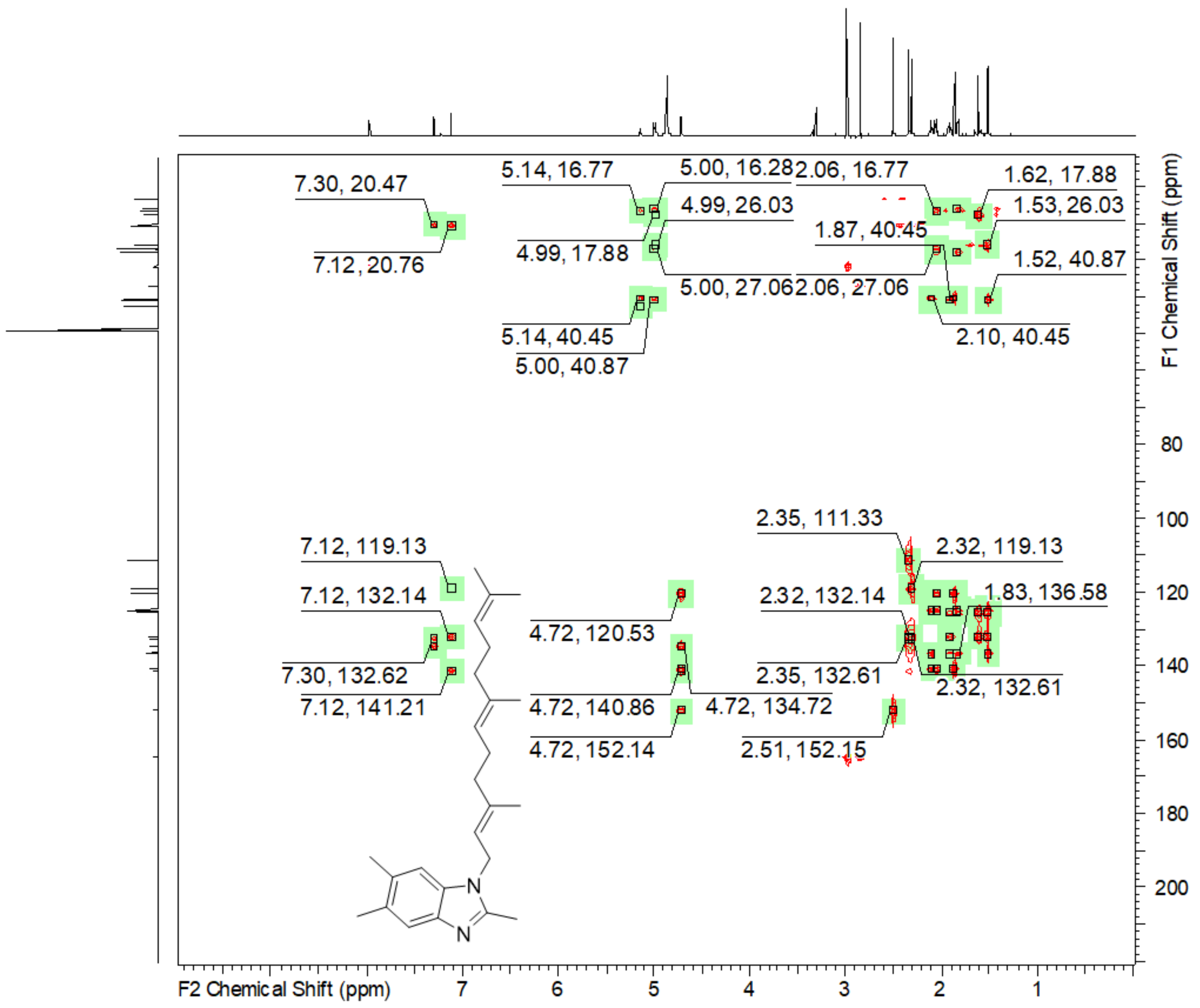

Figure S 41 HMBC spectrum of **3** in MeOD-d_4 at 700/175 MHz.

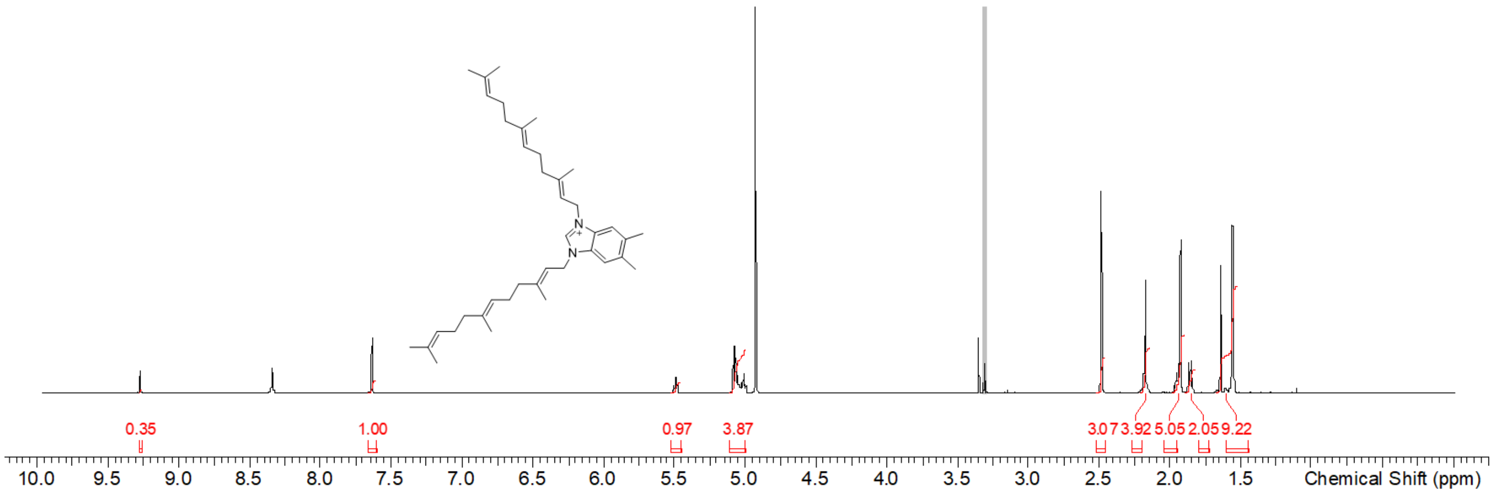

Figure S42 ^{1}H spectrum of **4** in MeOD-d_4 at 500 MHz.

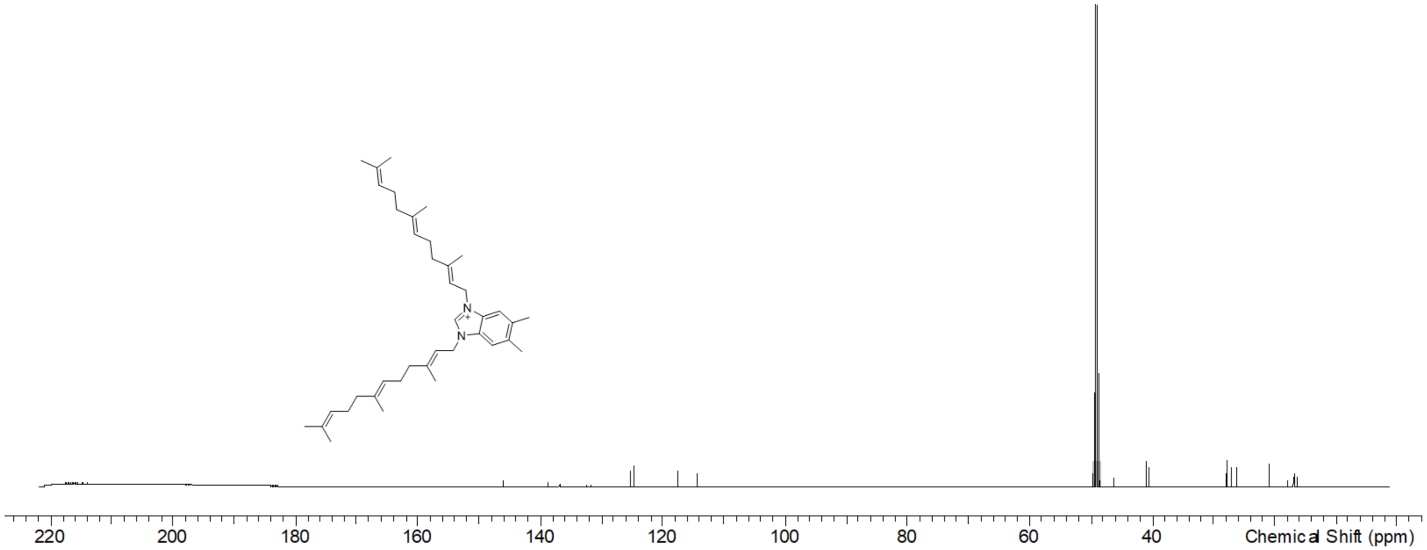

Figure S43 ^{13}C spectrum of **4** in MeOD-d_4 at 125 MHz.

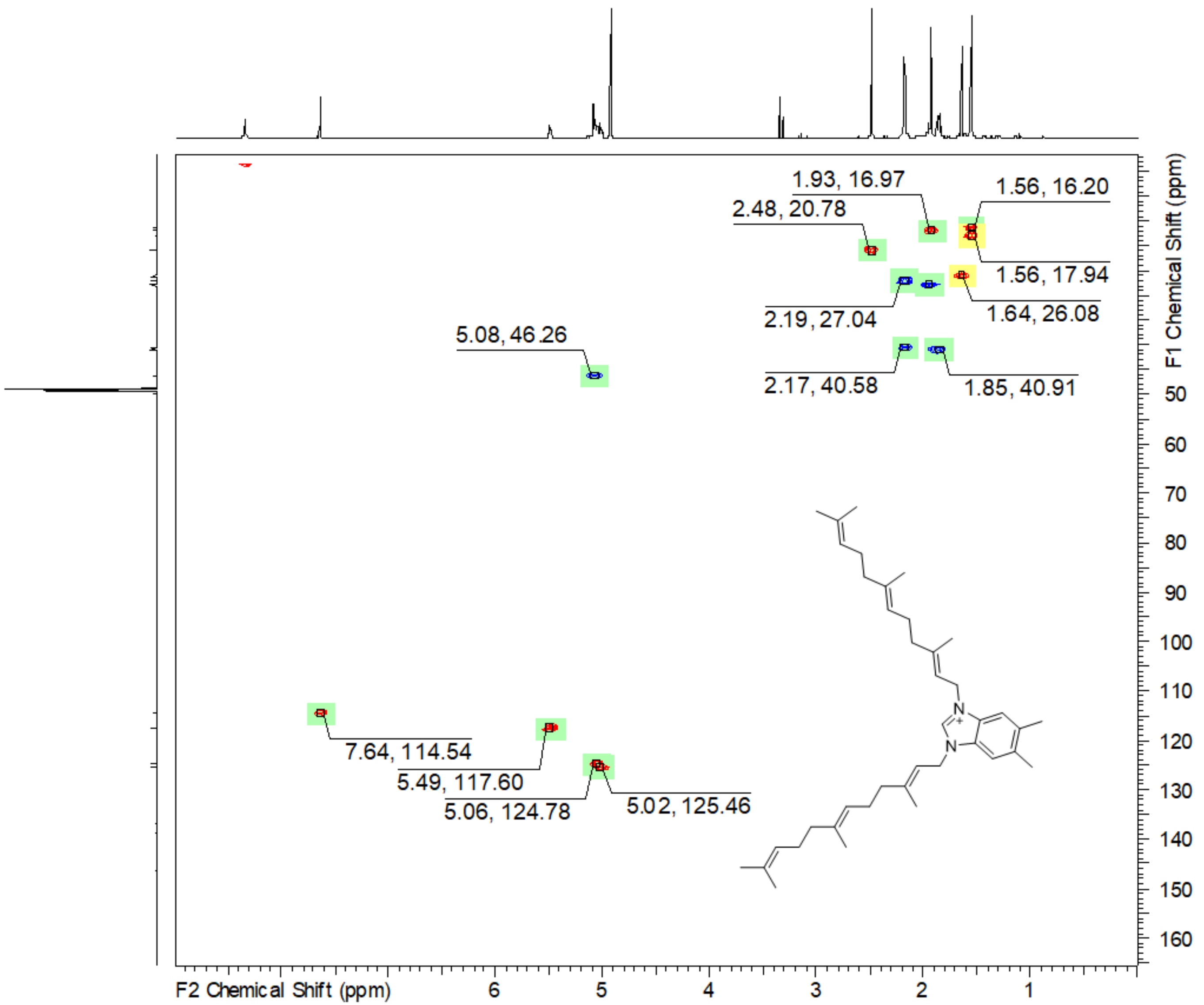

Figure S44 HSQC spectrum of **4** in MeOD-d_4 at 500/125 MHz.

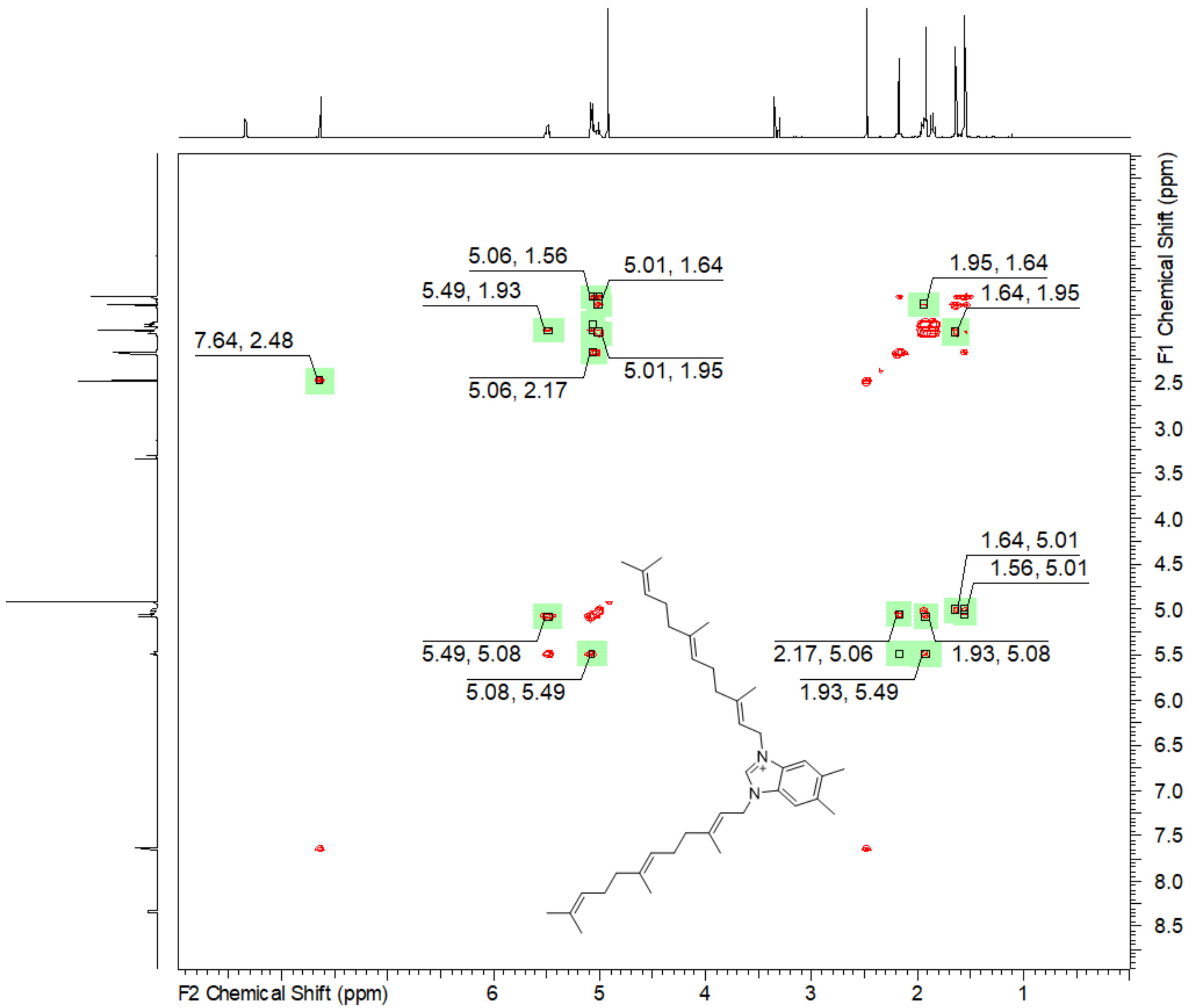

Figure S45 COSY spectrum of **4** in MeOD-d_4 at 500 MHz.

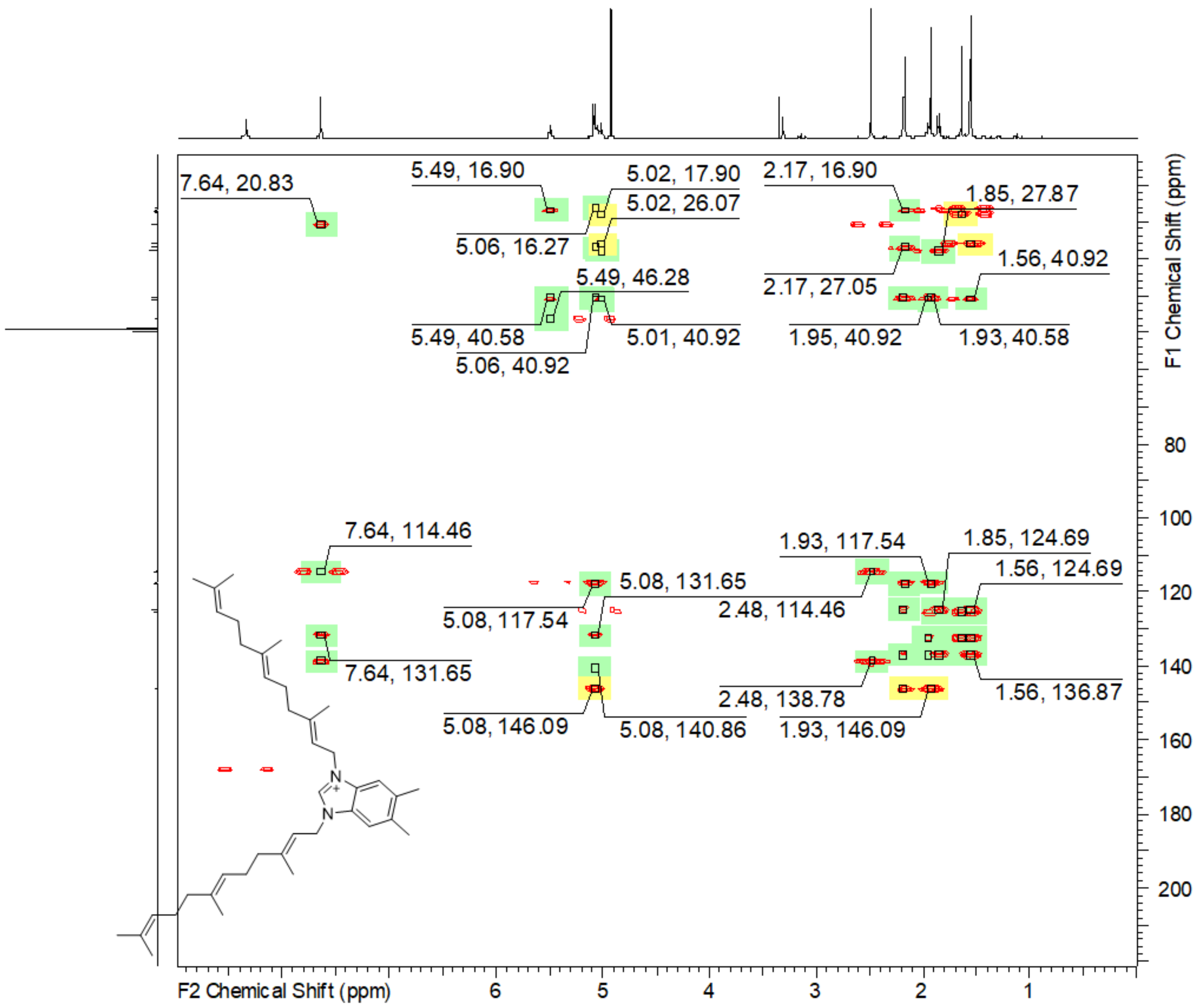

Figure S46 HMBC spectrum of **4** in MeOD-d_4 at 500/125 MHz.

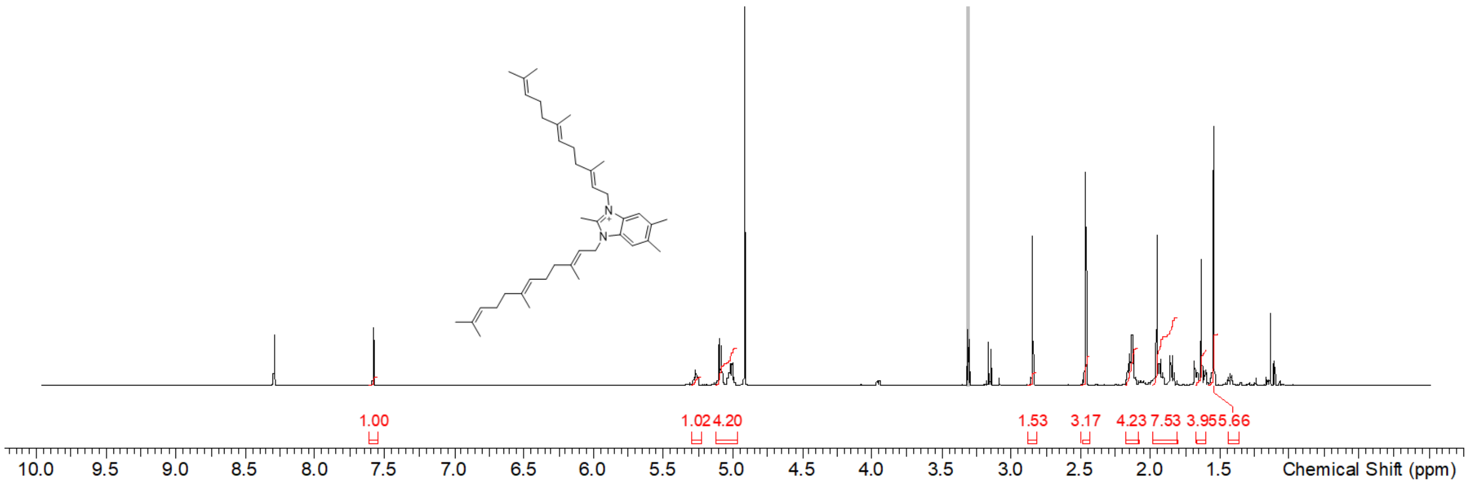

Figure S47 ^{1}H spectrum of **5** in MeOD-d_4 at 500 MHz.

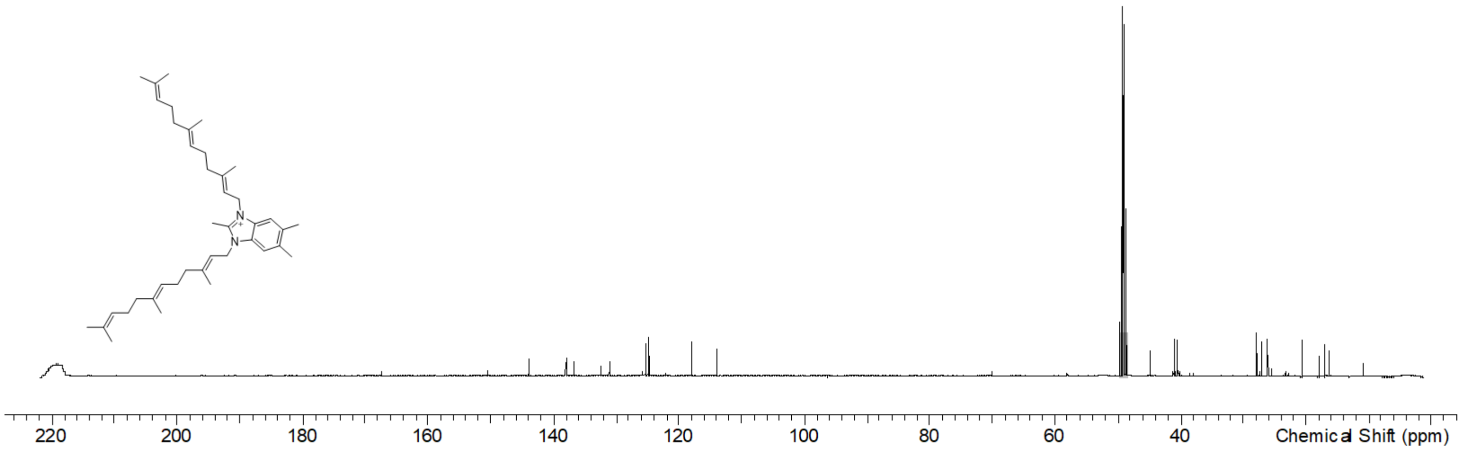

Figure S48 ^{13}C spectrum of **5** in MeOD-d_4 at 125 MHz.

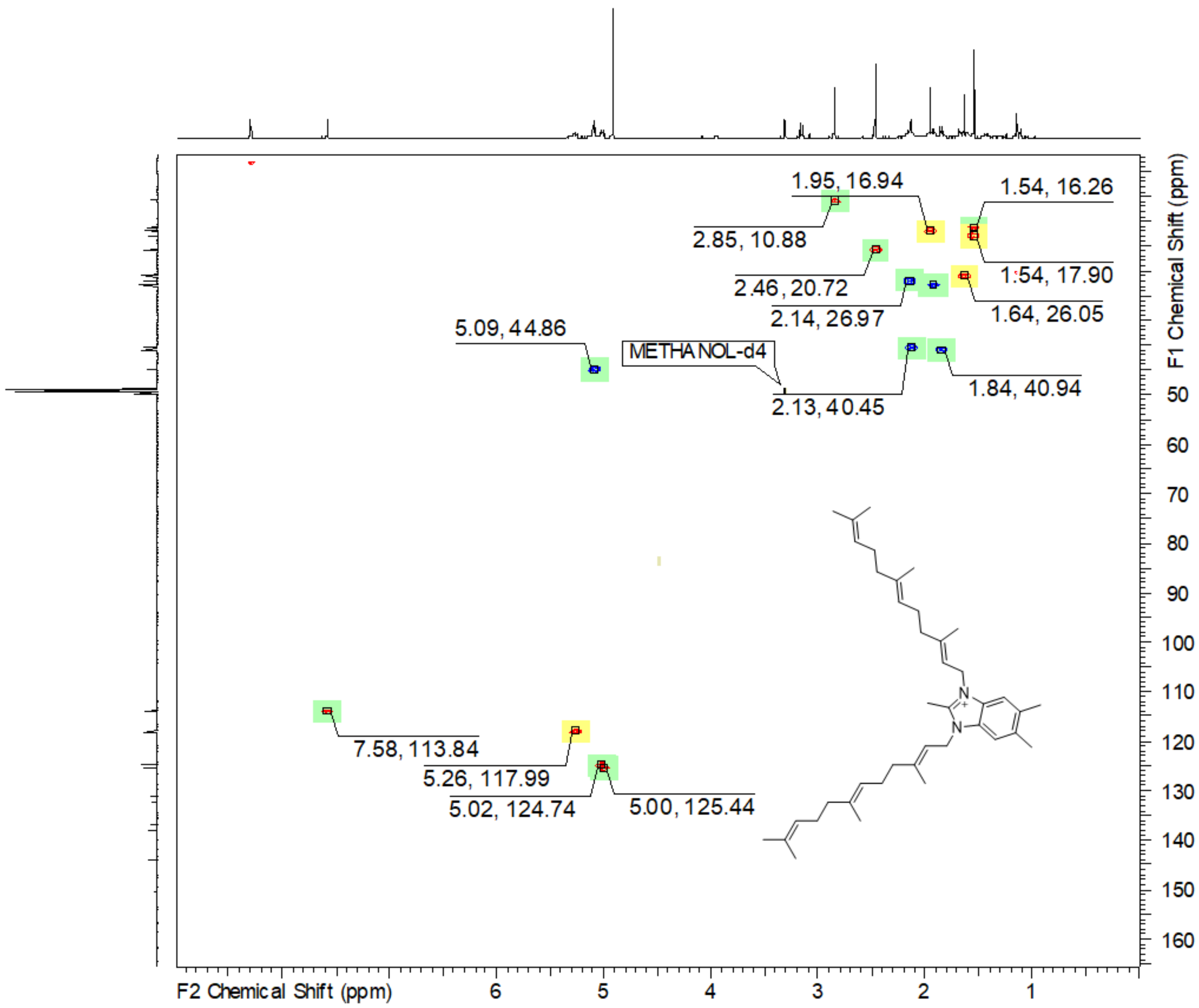

Figure S49 HSQC spectrum of **5** in MeOD-d_4 at 500/125 MHz.

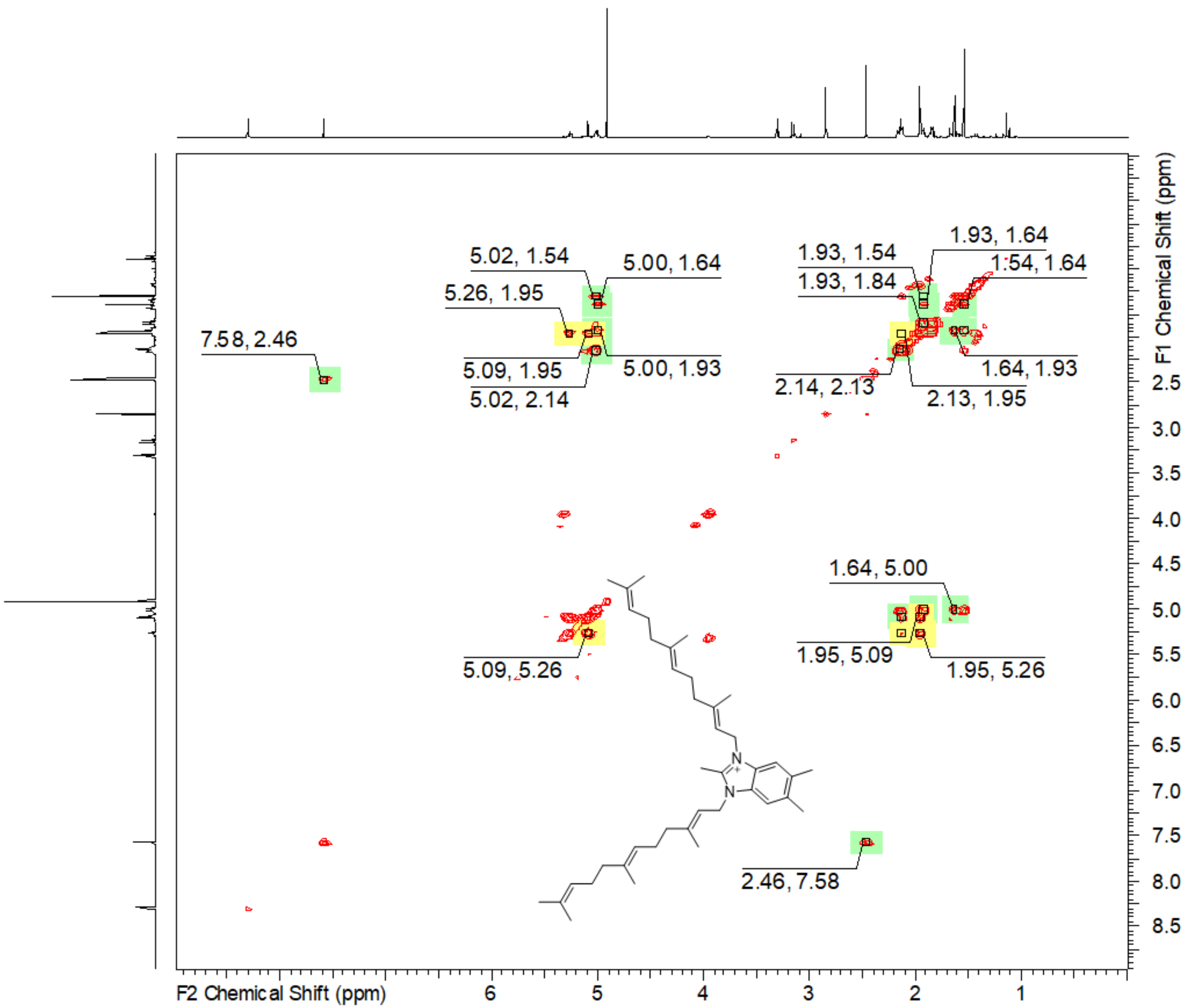

Figure S50 COSY spectrum of **5** in MeOD-d_4 at 500 MHz.

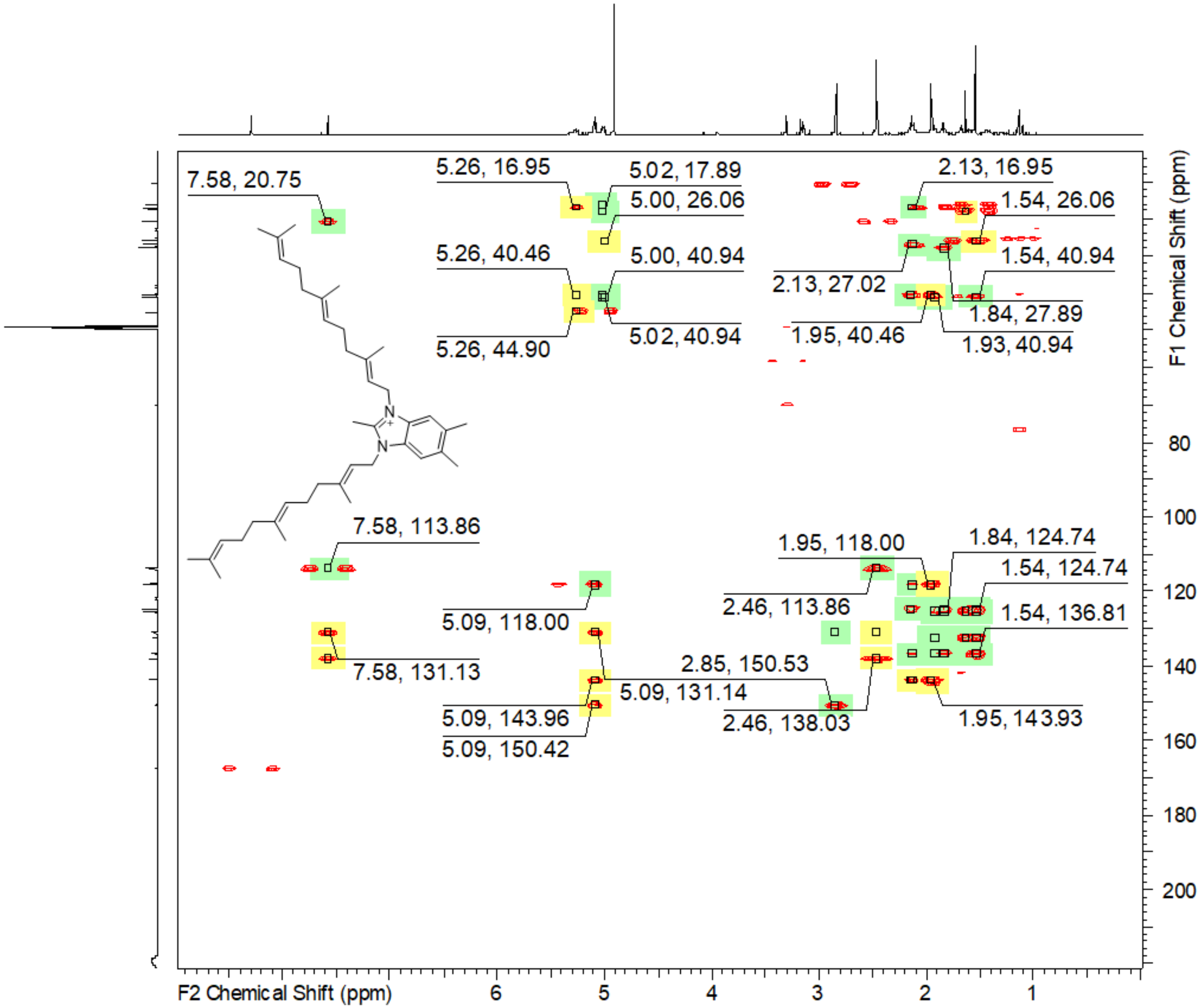

Figure S51 HMBC spectrum of **5** in MeOD-d_4 at 500/125 MHz.

S9 References

[1] J. Goris, K. T. Konstantinidis, J. A. Klappenbach, T. Coenye, P. Vandamme, J. M. Tiedje, *Int. J. Syst. Evol. Microbiol.* **2007**, *57*, 81.

[2] C.-H. Lim, S. Ilic, A. Alherz, B. T. Worrell, S. S. Bacon, J. T. Hynes, K. D. Glusac, C. B. Musgrave, *J. Am. Chem. Soc.* **2019**, *141*, 272.

[3] S. Llona-Minguez, A. Höglund, S. A. Jacques, L. Johansson, J. M. Calderón-Montaño, M. Claesson, O. Loseva, N. C. K. Valerie, T. Lundbäck, J. Piedrafita et al., *J. Med. Chem.* **2016**, *59*, 1140.

[4] Y. Rahbar Saadat, N. Saeidi, S. Zununi Vahed, A. Barzegari, J. Barar, *BioImpacts : BI* **2015**, *5*, 25.

[5] M. I. Ahumada, R. A. Chorbadjian, *Chil. j. agric. res.* **2019**, *79*, 658.

[6] a) E. P. Tchesnokov, C. J. Gordon, E. Woolner, D. Kocinkova, J. K. Perry, J. Y. Feng, D. P. Porter, M. Götte, *J. Biol. Chem.* **2020**, *295*, 16156; b) C. J. Gordon, E. P. Tchesnokov, J. Y. Feng, D. P. Porter, M. Götte, *J. Biol. Chem.* **2020**, *295*, 4773; c) C. J. Gordon, E. P. Tchesnokov, E. Woolner, J. K. Perry, J. Y. Feng, D. P. Porter, M. Götte, *J. Biol. Chem.* **2020**, *295*, 6785.

[7] a) I. Berger, F. Garzoni, M. Chaillet, M. Haffke, K. Gupta, A. Aubert, *J. Vis. Exp.* **2013**, e50159; b) I. Berger, D. J. Fitzgerald, T. J. Richmond, *Nat Biotechnol* **2004**, *22*, 1583.
